# A text mining and ontology-based approach using phenotypes to obtain relevant literature for rare diseases

**DOI:** 10.1016/j.isci.2026.117183

**Published:** 2026-08-13

**Authors:** Jesús Pérez-García, Federico García-Criado, Florencio Pazos, Mónica Chagoyen, Elena Rojano, Pedro Seoane, Juan A.G. Ranea

**Affiliations:** 1 Department of Molecular Biology and Biochemistry, University of Malaga, 29010 Malaga, Spain; 2 Computational Systems Biology, National Center for Biotechnology (CNB-CSIC), 28049 Madrid, Spain; 3 Center for Biomedical Network Research on Rare Diseases (CIBERER), Instituto de Salud Carlos III (ISCIII), 28029 Madrid, Spain; 4 Institute of Biomedical Research in Malaga and Platform of Nanomedicine, IBIMA Plataforma BIONAND, 29590 Malaga, Spain; 5 Spanish National Bioinformatics Institute (INB/ELIXIR-ES), Instituto de Salud Carlos III (ISCIII), 28029 Madrid, Spain

**Keywords:** rare diseases, phenotypic profiling, text mining, language models, human phenotype ontology, semantic similarity

## Abstract

Diagnosing rare diseases remains a major challenge due to limited clinical knowledge and the frequent absence of diagnostic criteria. We present a digital framework that leverages large language models and biomedical text embeddings to bridge this gap. By mapping Human Phenotype Ontology terms to a shared vector space with millions of PubMed abstracts and full-text articles, our method enables phenotype-driven semantic search and ranks literature relevant to patient symptoms, even without explicit disease mentions. Validated on OMIM-derived benchmarks and applied to RASopathies, including NF1, Noonan, and Costello syndromes, our approach retrieved expected findings, supporting differential diagnosis and research. The framework is implemented in an open-source Python package, py-semtools, and it can be integrated into clinical decision support systems or adapted to other ontologies and corpora. This work demonstrates how AI-driven informatics can enhance rare disease diagnosis and exemplifies the role of digital tools in transforming precision medicine and healthcare delivery.

## Highlights

• Phenotype-based semantic search linking patient HPO terms to biomedical literature
• Hybrid AI and ontology-driven approach for knowledge corpus annotation
• Validation in complex RASopathies to accelerate literature-based differential diagnosis
• Release of the *py_semtools* Python library and accessible web portal

## Graphical abstract

**Figure:**
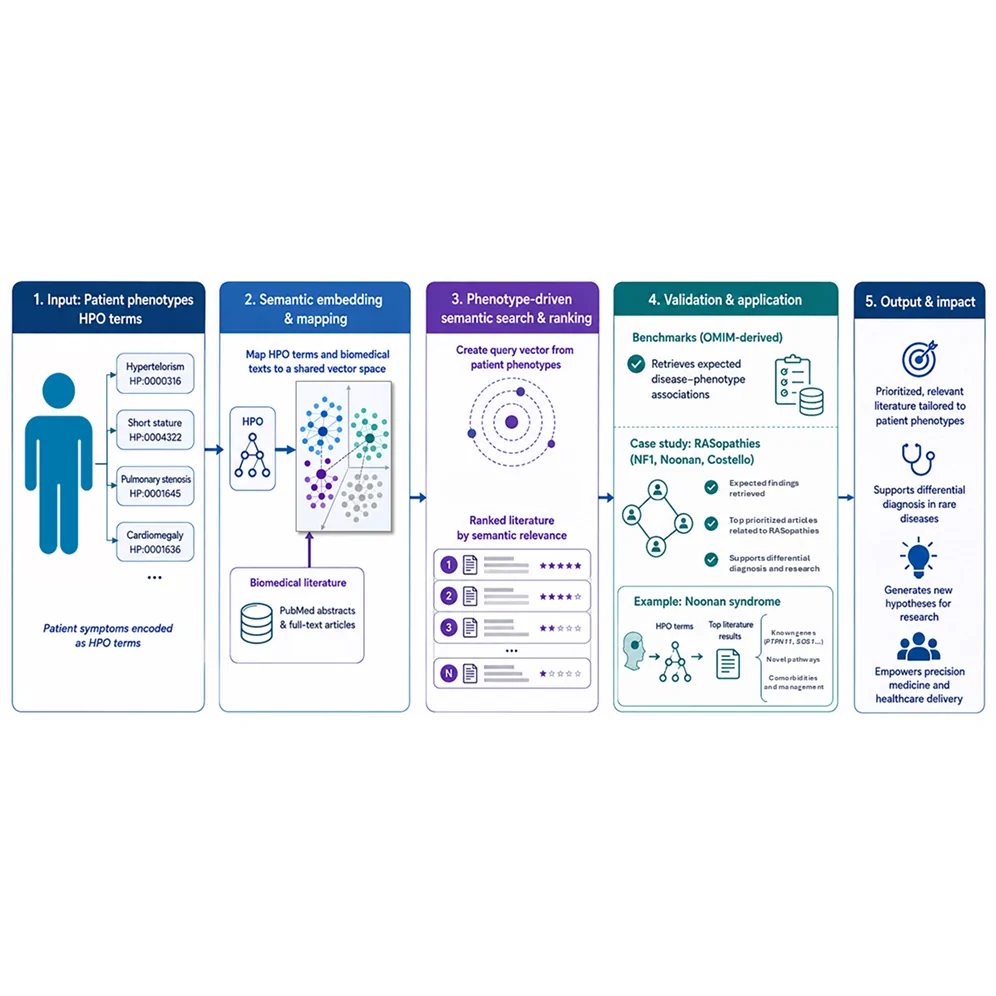

## Teaser

Health sciences; Medicine; Medical specialty; Health informatics

## Introduction

Rare diseases, while individually uncommon, collectively impact a significant proportion of the global population.^1^ With an estimated 7,000 distinct rare diseases affecting over than 400 million people worldwide, approximately 95% currently lack an approved treatment. Despite their diversity, rare diseases share similar clinical challenges: most healthcare professionals have limited or no experience in recognizing or managing most of these conditions, leading to significant delays in diagnosis and frequent misdiagnoses.^2^ These difficulties are further compounded by limited understanding of the underlying biological mechanisms, the complexity of navigating regulatory pathways, and the challenge of establishing appropriate and disease-specific clinical outcome measures.^3^

An essential first step in analyzing a rare disease case is the clinical characterization of the patient. However, the initial healthcare professional often lack the specialized knowledge required to recognize rare conditions.^4^^,^^5^^,^^6^ Typically, they become aware that the patient does not fit any familiar or previously encountered diagnostic pattern, as is the case with other common diseases, such as cancer, cardiovascular conditions or even COVID-19.^7^ At this point, healthcare professionals may turn to the scientific literature in search of comparable cases. Yet, this process is complicated by the large number of known syndromes and the overlapping nature of their clinical features. Additionally, the overwhelming volume of biomedical information can make it difficult to identify relevant and timely data, further complicating the diagnostic process.^8^

The life sciences and biomedical fields have generated a broad body of knowledge over decades of research, forming a critical foundation for future scientific advances.^9^ However, much of this information remains unstructured, dispersed across text, tables, and figures in millions of academic publications. This lack of standardized structure presents substantial barriers, not only for researchers seeking to manually retrieve relevant insights, but also for more advanced applications such as programmatic access, data integration, and large-scale computational analysis.^10^ As a result, valuable knowledge often remains hidden, particularly in time-sensitive or complex contexts such as the diagnosis and study of rare diseases. In these cases, the ability to efficiently extract and interpret relevant information from the literature can be crucial for guiding clinical decisions and identifying potential research directions.^11^ For this, the development of methods that can systematically transform unstructured biomedical literature into structured, computable knowledge is essential.

To facilitate knowledge representation for researchers and to enable programmatic management, various approaches have been developed to represent information in a structured and standardized manner. Among these, standardized vocabularies and ontologies are particularly noteworthy. Ontologies are formal, structured, standardized, and extensional representations of a specific domain of knowledge,^12^ composed of explicitly defined classes, individuals, object and data properties, and the relationships among them.^13^ All elements are formally defined and expressed in a machine-readable format, thereby facilitating computational access, querying, and manipulation of knowledge. Moreover, ontologies are designed to ensure a meaningful correspondence between the formal representation and the real-world domain they aim to model. Their construction is typically a manual and meticulous process performed by domain experts to capture the specific characteristics and requirements of the domain. Additionally, these experts incorporate domain-specific constraints, which are essential for enabling logical inferences through deductive reasoning.^14^

One of the major challenges in leveraging ontologies is that much of the knowledge contained in scientific publications is not expressed using ontology-aligned terminology. Consequently, to fully exploit the potential of ontological frameworks, it is essential to map free-text terms found in the literature to their corresponding concepts within a specific ontology. However, this mapping process is inherently complex due to linguistic ambiguities and contextual variability, often requiring manual curation by domain experts. In recent decades, significant advances in the fields of text mining and natural language processing (NLP), particularly with the emergence of sophisticated language models (LMs), have substantially improved the automation and accuracy of this task.^15^ These models belong to the relatively recent and rapidly evolving field of deep learning, which aims to adapt neural network architectures to uncover complex patterns in sequential data types, such as natural language.^16^ Early efforts in this area were primarily based on recurrent neural networks (RNNs). However, RNNs exhibited significant limitations: their sequential training process limited parallelization, and they struggled to capture long-range dependencies within textual data, often failing to preserve contextual relationships between distant words.^17^^,^^18^ However, the development of architectures, such as Transformers, and the attention mechanism^19^ addressed these limitations by enabling parallelized training and improving the ability to model long-range dependencies. This breakthrough has driven significant advancements in the development of LMs, facilitating a wide array of novel applications in NLP.

In recent years, numerous general-purpose large language models (LLMs) have been developed based on the Bidirectional Encoder Representations from Transformers (BERT).^20^ Several of these models have been pre-trained on domain-specific biomedical corpora, resulting in specialized variants such as BioBERT^21^ and PubMedBERT.^22^ These models have enabled the emergence of a wide range of context-specific NLP tasks in the biomedical domain. One notable example is named entity recognition (NER), in which models are trained to identify and classify entities within unstructured text, including names of individuals, locations, biological entities, and ontological terms.^23^

A representative example of biomedical NLP applications is PubTator 3,^24^ which employs NER to annotate six core biomedical entity types: genes, diseases, chemicals, genetic variants, species, and cell lines, and subsequently applies relation extraction (RE) techniques to identify twelve common types of relations among them. This approach is implemented in a web-based platform that allows users to query pairs of entities and retrieve relevant publications based on their inferred relationships. In parallel, other LMs have been designed to assess semantic similarity between text fragments, enabling the identification of conceptually equivalent expressions despite lexical variation.^25^ These models facilitate tasks such as concept detection and normalization within unstructured biomedical texts, even in the absence of exact matches.

Although LMs effectively learn representations of language and its embedded information, they are not error-free. Notably, LMs are susceptible to hallucinations, generating inaccurate or misleading statements, and often exhibit insensitivity to negations. Moreover, their capacity for logical reasoning remains limited, and accurately recognizing and extracting detailed, domain-specific information continues to be a significant challenge.^12^ In this context, extracting domain knowledge within the framework of established knowledge bases is highly advantageous. Ontologies, as formal and symbolic representations of knowledge, can complement LMs by addressing some of their limitations. They help bridge the semantic gap, enabling models to move beyond superficial pattern recognition and potentially capture the deeper underlying meaning of the data.^13^ Combining the strengths of LMs with the structured properties of ontologies can be a promising approach. There are trained LMs that can compute a semantic similarity measure between two text fragments and can be used to find semantically similar sentences.^25^ These models can be used to map ontological concepts from the phenotypic domain to textual entities within the literature. In this study, domain-specific ontologies such as the Human Phenotype Ontology (HPO)^26^ are used for this mapping process. This approach can be implemented programmatically at large scale to analyze extensive knowledge repositories, such as PubMed, enabling phenotypic-level annotation of articles. When a patient affected by a rare disease is phenotypically characterized using the HPO, the formal structure of the ontology facilitates direct comparison between the patient profile and the annotated literature. This ontology-driven similarity search provides an effective strategy to identify the most relevant publications for the patient case, overcoming limitations inherent to traditional keyword-based search methods. In this way, a large corpus of biomedical knowledge can be efficiently processed using minimal computational resources, yielding annotations based on a structured ontology that facilitates easy curation by interested researchers. Moreover, these ontological annotations enable the use of classical similarity algorithms to rapidly and accurately compare studies, allowing for the assessment of shared and distinct phenotypic terms across them.

This rationale motivated the development of *py-semtools* (Semantic Tools), a Python package designed to (1) manage and interact with ontologies, and (2) use embedding-based LMs to map textual entities to ontology terms within large text corpora. By applying this approach, we aimed to map the entirety of PubMed documents using the HPO and enable an ontology-based similarity search to identify the most relevant publications corresponding to the phenotypic profile of patients with undiagnosed rare diseases. Furthermore, this similarity search quantifies the weight of individual phenotypes, providing guidance to clinicians when selecting pertinent documents for a specific patient, thereby supporting clinical decision-making.

We present a case study applying this procedure to a well-known group of developmental disorders collectively referred to as RASopathies. RASopathies are a family of rare genetic syndromes caused by mutations affecting the Ras/MAPK signaling pathway, which plays a critical role in cell proliferation, differentiation, and survival.^27^ These syndromes share overlapping phenotypic features, including distinctive facial characteristics, cardiac anomalies, cutaneous manifestations, and varying degrees of neurodevelopmental impairment, making their clinical diagnosis challenging.^28^ For this study, we focused on five representative RASopathies: Neurofibromatosis type-1 (NF1),^29^ Neurofibromatosis-Noonan syndrome (NFNS),^30^ Noonan syndrome,^31^ Costello syndrome,^32^ and Cardiofaciocutaneous (CFC) syndrome.^33^ By leveraging our ontology-based similarity search approach, we aimed to enhance the retrieval of relevant literature matching the phenotypic profiles associated with these syndromes, thereby demonstrating the utility of this method in supporting diagnosis and research in rare genetic diseases.

## Results

### New software developed for semantic operations

Based on our previous experience in the computational analysis of biomedical concepts, including the construction of concept networks^34^^,^^35^ and the management of biomedical ontologies,^36^^,^^37^^,^^38^^,^^39^ we developed *py-semtools* (Semantic Tools), a Python library designed to facilitate ontology management and semantic text processing. Its use was fundamental for obtaining the results presented in this study, enabling the systematic analysis and comparison of phenotypic profiles across biomedical texts. The package can be downloaded from the Python Package Index (PyPI) under the name *py-semtools* (https://pypi.org/project/py-semtools/).

### Py-semtools: A python library to build semantically based analysis

In the context of ontologies, *py-semtools* supports a wide range of operations, which can be categorized into four main groups: the first two being specific to individual terms, while the latter two apply to sets of terms associated with a particular entity referred as “phenotypic profiles,” which are composed of HPO terms. First, term-level operations allow users to access specific attributes of an ontology term, such as its name, description, and cross-references. Additionally, these operations facilitate the retrieval of hierarchical relationships, including children, parents, and more distant ancestors or descendants, as well as calculation the information content of a term. Second, term-to-term operations, including determining the last common ancestor (LCA) and the maximum information common ancestor (MICA) for pairs of terms, which are essential for semantic similarity assessments. Third, profile-level operations enable the management and refining of term sets assigned to an entity (i.e., patient, gene and disease, among others). And fourth, full-profile operations that allow users to compute general term frequencies, compare individual profiles against a reference set, perform pairwise comparisons across multiple profiles and their clustering based on their ontological similarity.

Additionally, *py-semtools* provides visualizations that are essential for ontology-based analyses, representing term distributions across ontology and comparing ontology profiles (see STAR Methods section). Furthermore, this library supports loading any ontology that adheres to the Open Biological and Biomedical Ontologies (OBO) format as defined by the OBO Foundry (https://obofoundry.org/), including widely used ontologies: HPO, Monarch Disease Ontology (MONDO), Disease Ontology (DO) and Gene Ontology (GO).

For semantic text analysis, *py-semtools* implements the get_corpus_index module for corpus text retrieval along with the fmEngine, stEngine and llmEngine modules, which allow users to efficiently process and analyze text corpora and perform text semantic analysis.

#### Indexing large text corpora with get_corpus_index

**Figure 1:**
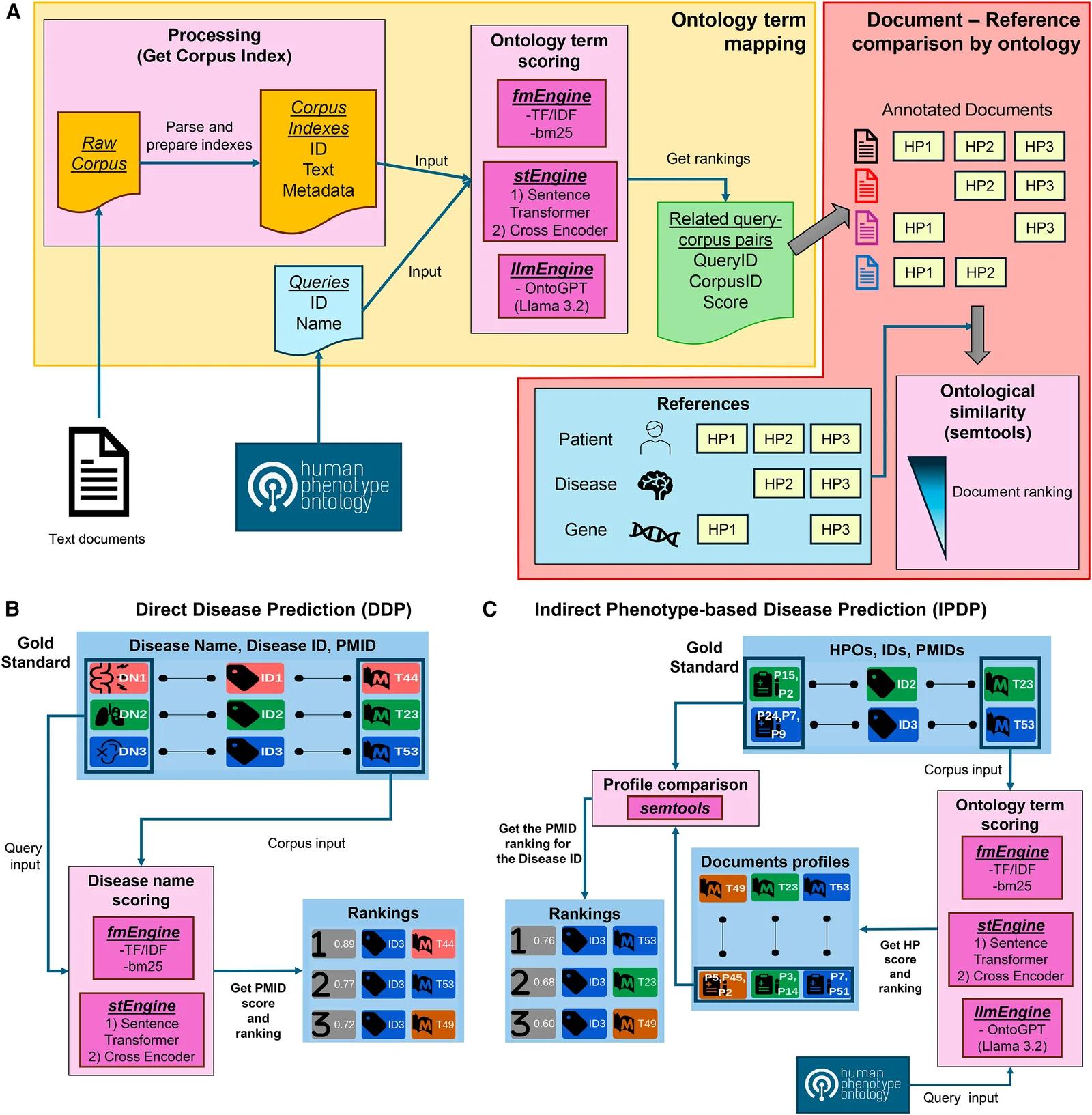
Graphical workflow overview (A) Workflow for obtaining query-corpus pairs (green box) with py-semtools engine modules from queries (blue box) and from the corpus processed with get_corpus_index (orange boxes). In this study, the input is the PubMed documents and the HPO ontology. The HPO-annotated documents are compared to a disease-reference profile (which could also be a patient or gene phenotypic profile) to generate a ranked list of documents, with those showing the highest phenotypic similarity appearing at the top. (B) In the direct disease prediction (DDP) workflow, disease names from the gold-standard dataset are used as queries, while the known associated documents serve as the corpus. The py-semtools engine modules score and rank these query-document pairs, and the rankings are compared against the gold standard for benchmarking purposes. (C) In the indirect phenotype-based disease prediction (IPDP) workflow, the known gold-standard documents are the corpus, while the HPO terms serve as the queries. Once the documents are HPO-annotated, their ontological similarity to the known HPO profiles of the gold-standard diseases is computed. This similarity score is then used to rank the documents, and the resulting rankings are compared against the gold standard for benchmarking purposes.

The text inside the corpus of scientific publications is an unstructured data type that can be found in a variety of formats. A first step to use this data in a machine-readable way, as shown in Figure 1A, is to transform the raw text corpus from different sources into corpus indexes that facilitate the following text operations. The get_corpus_index module, included in the *py-semtools* package, is designed to generate these corpus indexes from documents, regardless of the source format.

Among its functionalities, it extracts and prepares the content of different text corpora for use by the stEngine module. It includes basic raw text parsers and XML parsers for abstracts and full-text articles from compressed PubMed files. For the latter, text extraction is performed by identifying the appropriate XML tags according to the document type, excluding citations, tables, figure captions, and references. In the case of full-text articles, we discarded those documents that were compendiums of conference abstracts, eliminating those whose titles included the following keywords: “posters,” “abstracts,” “annual meeting,” “conference,” “congress” and “oral presentations,” trying to restrict to peer-reviewed relevant literature. Additionally, get_corpus_index can be extended to include parsers for documents from other sources with different structures.

The module also splits the documents into smaller units, first dividing the document hierarchically into paragraphs and then into sentences within each paragraph, using full stops and commas as separators, although other types of separation are supported.

Furthermore, get_corpus_index generates an index compatible with the stEngine module, including the corpus ID, the processed document (with or without splitting), and additional metadata fields such as title, publication year, original filename, total character count, number, and length of split sentences, as well as document type and category, to facilitate further evaluations if required.

This module also ensures the efficient distribution of the documents among the corpus indexes to balance the computational load during the file processing stage. Handling large numbers of PubMed documents can be computationally expensive due to the size and complexity of the data, especially when considering full-text articles and abstracts that require parsing, indexing, and processing. To mitigate this, the module employs parallelization strategies and optimizes the allocation of resources, distributing input files based on their size.

#### Document and ontology processing using py-semtools text analysis engines

In the specific context of this work, we seek to map textual entities in the text corpus to a formal representation of language, such as the terms of an ontology. Therefore, two sets to compare are defined: the set of terms of a given ontology (referred as “queries”) and the set of documents where the ontology terms will be searched (referred as “corpus”). As we can see in Figure 1A, we developed the Engine modules to perform the analysis of the “query” and “corpus” sets and for the subsequent comparison between pairs of elements of both sets.

Once the text corpus has been processed, our *py-semtools* package provides several approaches for performing semantic text analysis and obtaining term annotations from the desired ontologies. Users can load one or more text files as queries and one or more corpus files formatted according to the output of the get_corpus_index module. The corpus text can be divided into smaller units for finer-grained analysis. Depending on the implemented algorithm, we have designed three modules or engines for processing and analyzing texts in semantic similarity tasks: fmEngine, stEngine, and llmEngine.

The fmEngine is focused on algorithms based on full-match search, and the ontology terms are searched in the documents using approaches such as TF-IDF^40^ or BM25.^41^ This engine can deal with large datasets such as full PubMed using only CPU with very low execution times. Also, this engine is used as a baseline in our benchmarks to compare traditional approaches with true semantic ones.

The stEngine module processes and analyzes texts using lightweight computational models based on Sentence Transformers,^25^ which convert text fragments into high-dimensional vector embeddings that capture their semantic meaning. Unlike exact-match methods, which cannot detect semantic similarity between different words or phrases, these models can effectively capture and represent such relationships. Additionally, this module allows users to activate a second-stage reranker using a Cross Encoder model^42^ available within the Sentence Transformer package to refine the ranking results. To handle large text datasets, GPU resources are required; however, this enables the processing of large corpora in very short times. Note that if the reranker is activated, computation times will approximately double.

The llmEngine module includes algorithms based on full LLMs, aiming to achieve the highest possible accuracy, albeit at the expense of longer computational times and substantial hardware requirements. We have implemented the OntoGPT library,^43^^,^^44^ which integrates LLMs with ontology schemes and grounding to target ontology terms, enabling ontological annotations to be generated from any text corpus.

All the *py-semtools* engines are optimized for computational efficiency, supporting both CPU and GPU parallelization according to the algorithm. When possible, the engines also enable parallel processing across multiple GPUs, with features for efficient load balancing and memory management.

### Web portal availability

We developed a Django-based web application to enable user queries of HPO terms and retrieval of relevant PubMed documents. This tool interfaces with our HPO-annotated database, generated using the stEngine module and the HPO (available at https://www.clinsysbiolab.es/). To ensure query feasibility with a response time of under 15 s per document, we implemented a pipeline optimized through a RASopathies case study, exploring different parameters to perform the searches.

The optimization process involves several stages. First, we filter all HPO-annotated abstracts based on a minimum threshold of terms per document. We then construct an annotation matrix (with document IDs and HPO codes, each one in a separate dimension) where intersections indicate the presence of a term in the document. To manage dimensionality, each profile is simplified by mapping terms to the descendants of the “Phenotypic Abnormality” root. This reduces the HPO dimension from approximately 14,000 terms to a few hundred. These simplified matrices are then partitioned using a K-means clustering algorithm. For each cluster, we generate a representative profile composed of its most frequent HPO terms.

During a search, the input profile of the user is first compared against these representative cluster profiles to identify the best matches. The system then retrieves and ranks the associated PubMed documents within the top-performing clusters. We fine-tuned this pipeline by evaluating various parameters: the minimum HPO annotations per document, the hierarchical level for term simplification (e.g., direct children vs. grandchildren of “Phenotypic Abnormality”), the number of K-means clusters, the frequency threshold for representative profiles, and the number of top clusters scanned (see the Table at Data S1).

Performance metrics for these configurations are detailed in Figure S9. We selected a configuration that achieves a PRER-AUC of 0.71 and retrieves approximately 5% of the RASopathies gold-standard set within the 15-s limit. This optimal setup includes abstracts with at least three HPO terms, simplification at the “grandchild” level, 500 clusters, a 0.1 frequency threshold for representative terms, and a search depth of the top ten clusters. While the web portal computational constraints limit the exhaustive analysis provided by the standalone version, it serves as a powerful entry point for non-technical users. This platform offers a streamlined environment to explore its potential and validate its utility before committing the technical resources required for a full-scale local deployment.

### Gold-standard datasets exploratory analysis

To evaluate the performance of our methodology, which maps ontology terms to literature documents, we benchmarked it using datasets linking disease nomenclature to PubMed IDs. Prior to this evaluation, we characterized the disease-document gold-standard datasets: “OMIM-Erhart,” “OMIM-DO” and “MPS” (see STAR Methods), to examine the relationships among documents, diseases, or patients, and the corresponding phenotypic profiles.

The number of diseases/patients and documents considered in each evaluation workflow is summarized in Tables S1 and S2, for both the direct disease prediction (DDP) and indirect phenotype-based disease prediction (IPDP) evaluation workflows, respectively.

For both “OMIM-Erhart” and “OMIM-DO” gold-standard datasets, a very similar number of raw articles is observed, regardless of the prediction method used. It is noteworthy that the number of abstracts and full-text articles is slightly lower in the “OMIM-Erhart” dataset, but the proportion of unique PMIDs remains very similar in both gold-standard datasets and prediction methods. For the “MPS” gold-standard dataset, a contrasting pattern is observed: the DDP benchmark includes fewer documents (abstracts and articles) and fewer diseases, whereas the IPDP benchmark includes more documents. This difference arises because the IPDP benchmark is adapted for “MPS” as multiple patients are associated with each disease. In this configuration, the algorithms annotate documents with phenotypes, which are then ranked according to the phenotypic profiles of each patient sharing the same disease. Consequently, the dataset comprises 207 diseases for the DDP benchmark and 1,420 patients for the IPDP benchmark. On average, each article corresponds to between four and five patients when repetitions of PMIDs are considered.

**Figure 2:**
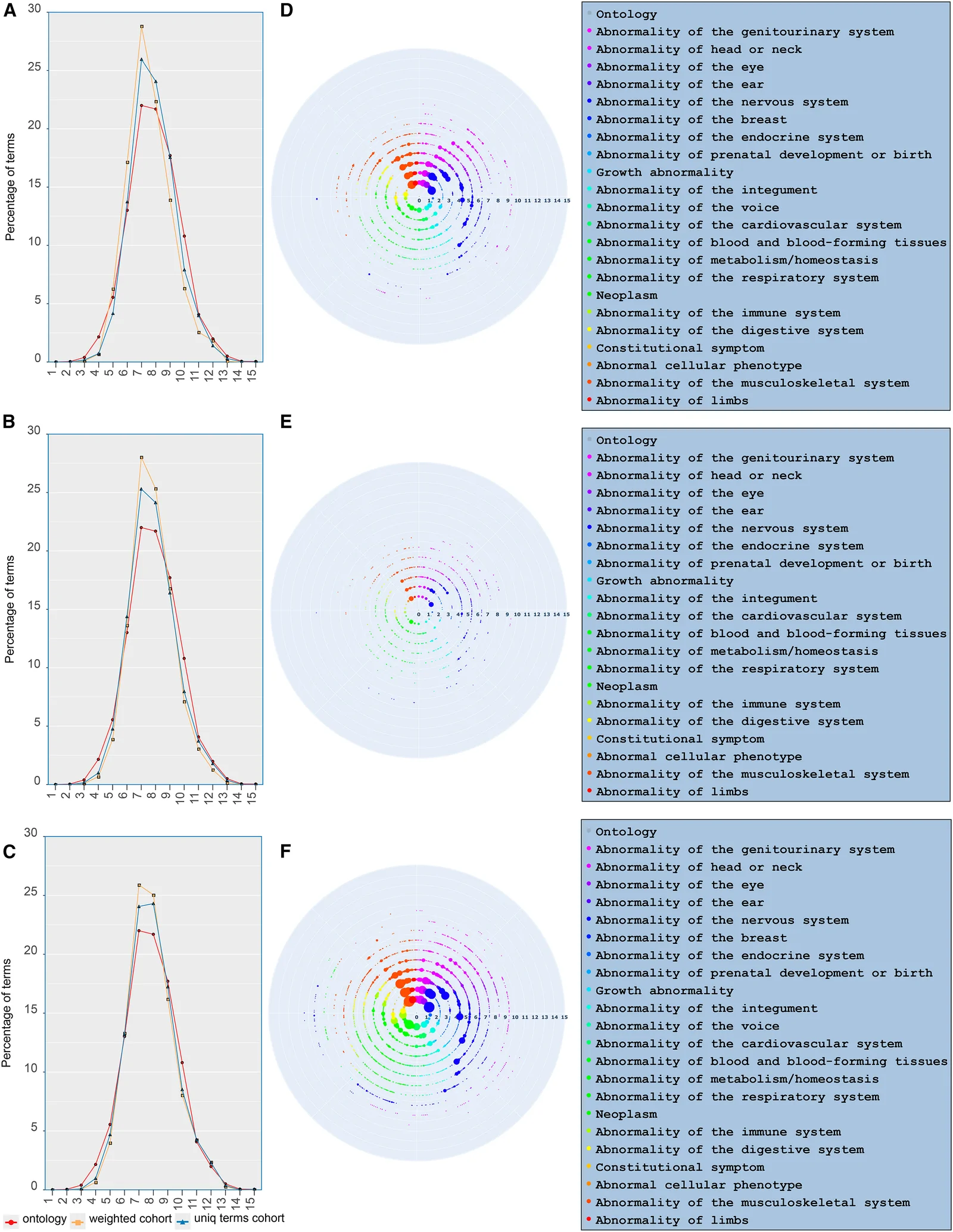
Phenotypic distribution and ontological signatures for “OMIM-Ehrhart” Depth level representation of phenotypes (shortest path from the root) for diseases and annotated documents associated with the “OMIM-Ehrhart” gold-standard dataset generated with py-semtools. (A) Dataset diseases. (B) Annotated abstracts with PubMed identifiers (PMIDs). (C) Annotated full-text articles with PubMed Central identifiers (PMCIDs). Ontological signatures of diseases and annotated documents associated with “OMIM-Ehrhart” gold-standard dataset generated with py-semtools. (D) Ontological signature of dataset diseases. (E) Ontological signature of annotated abstracts with PMIDs. (F) Ontological signature of annotated full-text articles with PMCIDs. The center of the circle represents the phenotype “phenotypic abnormality,” with the HP:0000118 code, which is taken as the root term. Each concentric circle represents one level of the ontology, from the center outward. Each phenotype is represented at the level equivalent to its depth, defined as the shortest path from the root term of the ontology. The colors represent the child phenotypes of the root term, used to categorize at a general level to which branch more specific phenotypes belong. The dot size of each phenotype refers to the frequency with which it is found within the analyzed profiles. For each term present in the profiles, their parents were obtained and also taken into account when calculating their frequencies. A cut-off threshold was applied to eliminate those terms represented in less than 0.5% of the profiles.

To investigate potential differences in the phenotypic annotations of documents compared to disease profiles, we analyzed with *py-semtools* the distribution of HPO terms with a particular focus on their depth within the ontology hierarchy. The HPO used in this study (release 2024-04-19) includes 15 different levels. The depth of each phenotype, defined as the shortest path from the root term of the ontology, was computed for the terms associated with each disease in the “OMIM-Erhart” dataset (Figure 2A), for the HPO terms found in the abstracts (Figure 2C) and those found in the full-text articles (Figure 2E). As shown, the distribution of HPO terms is quite similar across the three cases. However, the original “OMIM-Ehrhart” dataset contains a large number of terms at level 7 of the ontology. When stEngine annotates the documents (here we use this execution as an example, since stEngine provides a good balance between result reliability and computational efficiency), the number of HPO terms at level 7 decreases, while those at level 8 increase. This shift indicates that the annotation process tends to favor more specific phenotypes.

To gain further insight, we conducted a detailed breakdown of the phenotype annotation frequency by depth level and by the main branches of the ontology, using the *py-semtools ontoplot* representation described in the STAR Methods section, which captures the ontological signature of each set of phenotypic HPO profiles (Figures 2B–2D and 2F). We can observe that when abstracts are annotated (Figure 2D), certain ontology branches, such as “Abnormality of the breast,” “Abnormality of the prenatal development of birth” and “Abnormality of the voice,” are not represented. Additionally, many phenotypes are missing. However, when using full-text articles, the ontological signature closely resembles that of the original dataset (Figure 2D) with the addition of HPO terms identified by stEngine that were not present in the original dataset. This trend is also observed in the “OMIM-DO” (Figure S1) and the “MPS” (Figure S2) datasets. In the latter case, the overall phenotypic signature in abstracts closely resembles that of the patient profiles, although frequencies are lower for more specific ontology levels. In full-text articles, higher frequencies are observed for both general and specific phenotypes, as well as for additional phenotypes not present in the patient cohort.

### Benchmarks: Measuring performance for direct and indirect prediction approaches

To evaluate the performance of the methodology, we applied the benchmarking workflows as described in the STAR Methods section: the DDP to identify diseases as OMIM-disease names, and the IPDP to identify diseases through the extraction of HPO terms from documents. Normalized ranking values, which represent the position of the correct PMID-OMIM ID combination according to the gold standard, were computed for the “OMIM-DO,” “OMIM-Ehrhart”, and “MPS” datasets, using PubMed abstracts and full-text articles. To enable comparisons across different conditions, ranking positions were normalized to the total number of documents evaluated in each case. This total corresponds to the sum of the documents in the gold-standard and the randomly added documents, ranging from 560 to 20,220 documents.

#### Evaluation of the optimum thresholds for each implemented algorithm

We have to consider that the DDP workflow evaluates disease entity identification; thus, the algorithms give a score or similarity value that allows ranking the documents directly. In this case, there is no need to select thresholds, but when we used the IPDP workflow, there are two steps: phenotype entity identification for each document and ontological similarity calculation using the retrieved phenotypes, with document ranking based on this similarity value. The second stage is ranked directly but depends on the reliability of the first, which we optimize by applying score thresholds to preserve reliable phenotype annotations. For this reason, we explore the most optimized values for these thresholds. In the case of the stEngine module for both the sentence transformer algorithm and its re-ranker step, can be seen in Figure S3A, for abstracts the best thresholds tie at the 0.7–0.8 values, with the exception of the MPS, where 0.6 is the best. Regarding articles (Figure S3B), all the datasets present the 0.7 threshold as the best. For this reason, we select the 0.7 value as the default threshold for the Sentence Transformer model in this work. For the re-ranker step in the stEngine, there is no homogeneous consensus (Figures S3C and S3D) either by dataset or abstract/full article, with 0.6–0.7 values the most optimized. For this reason, we select 0.6 as the default threshold for re-ranker scores.

In the case of the algorithms used in the fmEngine module, for the TF-IDF, it can be observed that the optimal threshold varies depending on the dataset and the specific document type; however, for articles (Figure S4B), thresholds of 0.7–0.8 yield the best results, and for abstracts (Figure S4A), the threshold of 0.8 appears to hold across the different datasets, so we chose the threshold of 0.8 for the scores obtained for TF-IDF.

For BM25, since there is no defined upper bound, different thresholds were tested in an attempt to identify a maximum within the range of thresholds tested, evaluating thresholds at values of 2, 4, 6, 8, and 10. It can be observed that for articles (Figure S4D), the results improve as the threshold is increased from 2 to 6, and then decline again when it is increased to 10, with the optimal cutoff threshold lying between 6 and 8. However, for abstracts (Figure S4C), differences are observed between the “MPS” dataset, where the optimal threshold is 4, and the “OMIM-DO” and “OMIM-Ehrhart” datasets, where the optimal threshold is 10. Therefore, it appears that the threshold, which is not bounded from above, is specific to the type and size of the corpus and could take different optimal values when using it for new datasets. In this case, considering the results as a whole for the two document types and the three evaluated datasets, a threshold of 6 was chosen.

#### Overall comparison for fmEngine and stEngine

**Figure 3:**
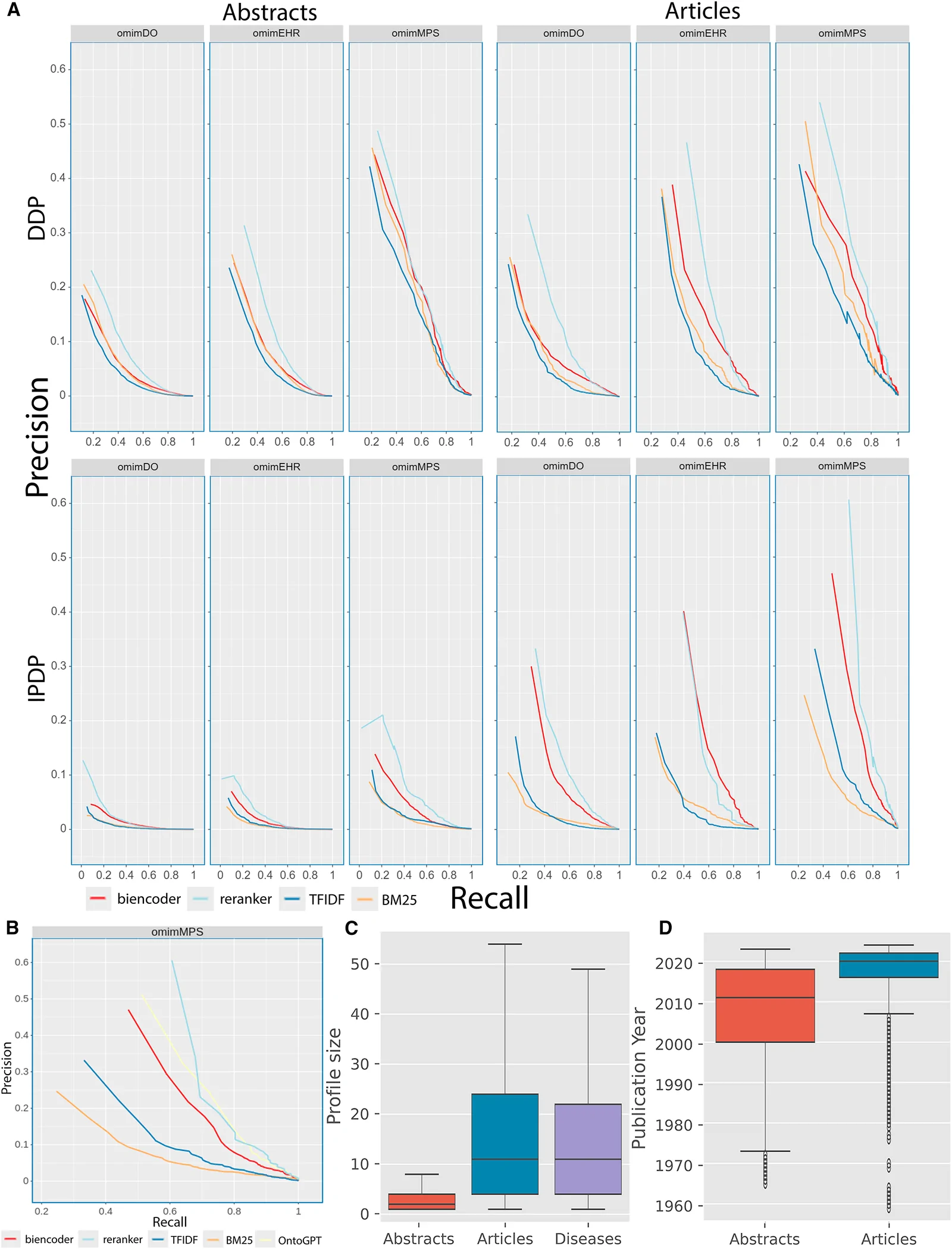
Evaluation curves and dataset metrics for DDP and IPDP (A) Precision and recall curves obtained using the gold-standard datasets and py-semtools text engines in both direct disease prediction (DDP) workflow and the indirect phenotype-based disease prediction (IPDP) workflow. These metrics are presented for abstracts and full text articles. (B) Precision/Recall curves obtained using the “MPS” gold-standard and py-semtools text engines, including OntoGPT for the IPDP workflow in the full-text articles case. (C) Size comparison of the phenotypic profiles of all annotated abstracts and full-text articles downloaded from PubMed and PubMed Central. Phenotypic profile sizes of all available OMIM diseases with at least one HPO term in the HPO annotations (file phenotype.hpoa) are added for reference. Error bars are defined as a 95% confidence interval. Outliers have been removed for better visualization. (D) Year of publication of abstracts and full-text articles downloaded from PubMed and PubMed Central. Error bars are defined as a 95% confidence interval.

Figure 3A presents the precision and recall results for each gold-standard dataset, considering both abstracts and full-text articles, using the fmEngine and stEngine modules with the optimal threshold values discussed in the previous section. Overall, the combination of the bi-encoder model with the re-ranking strategy achieves the best performance in nearly all scenarios, except for the “OMIM-Erhart” dataset during the IPDP benchmark with full-text articles. In this case, using only the bi-encoder model outperforms the re-ranking strategy. The bi-encoder alone is generally the second-best algorithm for most IPDP benchmarks and for some DDP benchmarks applied to full-text articles. Baseline models generally show poor performance, although they perform comparably to the bi-encoder model for abstracts in the DDP benchmark. The performance of these algorithms for top predictions is also presented in the “Measuring ranking performance in tops” section later in discussion.

#### Overall comparison for MPS, including fmEngine with OntoGPT

For the comparison of the presented algorithms using the llmEngine with OntoGPT, we developed a separate benchmark. This approach was necessary because OntoGPT extracts ontology terms from documents without producing any associated score or ranking and because it is a computationally intensive implementation that cannot handle all the listed gold-standard datasets. For this reason, we used the smallest gold-standard dataset, MPS, considering full-text articles for the IPDP benchmark, which generates scores based on ontology similarity values computed by comparing the annotated phenotype terms against the known phenotypes of the patients. In this case (Figure 3B), OntoGPT processing performs very similarly to the bi-encoder model with re-ranking, and in some instances even outperforms it, despite using a more complex pipeline and managing a full LLM model such as Llama 3.2.

#### Measuring ranking performance in tops

To further evaluate the performance of our methodology, we calculated the True Positive Rate (TPR) by determining the proportion of correct PMID-disease associations retrieved within the top 1%, 2%, 5%, and 10% of the ranked predictions and absolute ranking positions for the 1,2,5 and 10 top documents for the different methods proposed.

If we examine the percentage rankings for the DDP flow of the Sentence Transformers methods, with and without reranker, and the TF-IDF and BM25 methods (disease panel in Figures S5A, S6A, S7A, and S8A), we can see that, for each method, the results are consistent across datasets. Furthermore, it can be observed in general that abstracts achieve better TPR values than articles at low top-K thresholds, although the TPR values between the two types of documents tend to converge as the top-K threshold is increased, with the results being most similar when considering the top 10%. We can also observe that higher TPR values are obtained in the cases of Sentence-Transformers with or without reranker (Figures S5A and S6A) than with the TF-IDF or BM25 methods (Figures S7A and S8A).

If we look at the IPDP case (phenotype panel in Figures S5A, S6A, S7A, and S8A), consistent results are also observed across datasets, except in the case of BM25, where the results are more erratic. If we look at the dataset level, the results appear to be better in articles than in abstracts for Sentence Transformers with and without a reranker (Figures S5A and S6A), although this difference is not as notable in the case of TF-IDF (Figure S7A) and is even reversed for BM25 (Figure S8A). The best result is obtained in the IPDP case with Sentence Transformers without a reranker for the MPS dataset, where a TPR of 0.95 is achieved in the top 10% of articles.

If we examine the TPRs obtained using absolute top-K (Figures S5B, S6B, S7B, and S8B), patterns similar to those discussed above can be observed, with the difference that for the DDP case, better TPRs are typically observed for articles than for abstracts. This appears to be a pattern contrary to what we have seen with the percentage tops, which is due to the smaller number of full-text articles available compared to abstracts when prioritizing. We can also observe that in the IPDP case, the TPR rankings are better for Sentence Transformers with and without reranker than for TF-IDF and BM25.

#### Evaluation of computation time

Precision and recall results should be interpreted in the context of the computation times required by each algorithm. To this end, we measured execution times, calculating the average time per document and the relative time increase, using stEngine—bi-encoder only as a reference (Table 1). TF-IDF method is relatively slow, whereas BM25 and bi-encoder only exhibit similar execution times. The top-performing algorithms in the precision-recall curves, bi-encoder with re-ranking and OntoGPT (Ollama + Llama 3.2), show the largest time increases, 5*X* and 2919*X*, respectively. The OntoGPT approach is computationally unfeasible, whereas the re-ranking strategy is more reasonable, though a 5*X* increase is still prohibitive for processing the entire PubMed database, both abstracts and full-text articles, in a timely manner. For these reasons, stEngine—bi-encoder only is used in the following sections.

**Table 1:** Benchmarking of the methods' processing speed

	Time per document (seconds)	Time factor
fmEngine - TF/IDF	0.6	1.5*X*
fmEngine - bm2	0.3	0.76*X*
stEngine - bi-encoder only	0.39	1*X*
stEngine - bi-encoder + re-ranking	1.9	5*X*
llmEngine - OntoGPT (ollama + Llama 3.2)	1167	2919*X*

Computation time execution per document for each algorithm and time consumption factor calculated using the stEngine module only with the bi-encoder model as reference.

#### Analyzing the impact of phenotype characterization in disease identification

**Figure 4:**
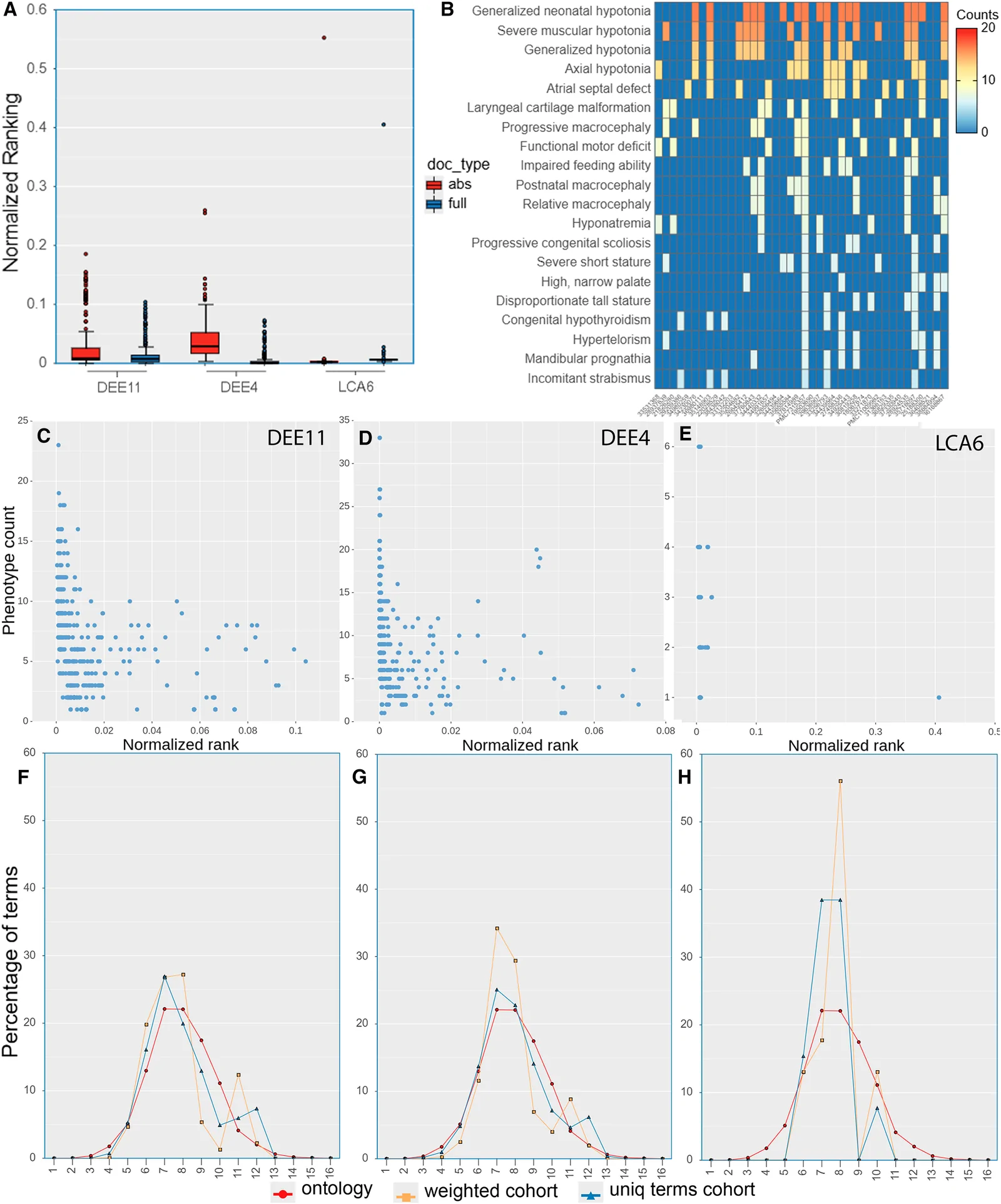
Patient ranking distributions, phenotypic specificity, and negative profile analysis (A) Patient ranking distributions for DEE11, DEE4, and LCA6 diseases using both abstracts and full-text articles. The remainder of the “MPS” dataset, plus 100 times that number of randomly selected articles, were used as negative examples (totaling 46,460 abstracts and 18,079 articles). Error bars are defined as a 95% confidence interval. (B) Negative heatmap shows the phenotypes that are absent and ontologically different from the patient’s profile and appear most frequently in the top 50 articles suggested for patient “pp2173.” (C–E) Comparison of the normalized ranking values obtained for each patient and the size of their phenotypic profile for DEE11 (C), DEE4 (D), and LCA6 (E). (F–H) Frequency distribution of the specificity levels of the phenotypes used to describe the patient cohorts for DEE11 (F), DEE4 (G), and LCA6 (H).

An important factor when searching for a disease or a patient case in the pool of HPO-annotated documents is the depth and breadth of the phenotypic descriptions. For this reason, we selected the three diseases with the largest number of associated patients that were linked to a single open-access PubMed article. Based on this criterion, the diseases “developmental and epileptic encephalopathy 11” (DEE11), with 393 patients, “developmental and epileptic encephalopathy 4” (DEE4), with 462 patients, and “leber congenital amaurosis 6” (LCA6), with 134 patients, were selected. Each patient set was ranked using the “MPS” gold-standard dataset, which includes the original publications used by the Phenopacket Store to extract patient phenotype data, together with 100*x* randomly selected documents. This resulted in a total of 36,600 documents for the abstract-based evaluation and 14,200 documents for the full-text evaluation. Figure 4A shows that there is a noticeable improvement when we use full text documents rather than abstracts, excepting for LCA6 disease. It is interesting to mention that different patients using the same document pool set have very different rankings. When we focus in full text document search and compare the normalized rank against the number of phenotypes associated to the patient, we found that there is a tendency to improve the ranking as the number of phenotypes increases (Figures 4C and 4D) and that above ten or 15 annotations phenotypes gives a clear disease hallmark. In the case of the LCA6 disease, this pattern is not observed (Figure 4E). However, this difference can be explained by another aspect of the phenotypic characterization: the specificity of patient phenotypes. If the distribution of patient phenotypes across HPO levels is examined (Figures 4F and 4G, weighted cohort series) 25%–35% of phenotype annotations for DEE11 and DEE4 are located at level 7, while an additional 25%–30% occur at level 8. In contrast, LCA6 shows 56.6% of phenotype annotations at level 8 (Figure 4H). Consequently, patients with LCA6 present highly informative phenotype annotations. Examination of these annotations shows that most belong to the HPO domain “Abnormality of the eye,” with a smaller representation from the domain “Abnormality of the nervous system.”

The previous results indicate that patient characterization using HPO terms is a key factor for identifying the underlying disease. For this reason, we developed a feature to support this process, termed the phenotype negative heatmap. This feature highlights phenotypes that appear in documents most similar to the patient characterization but are absent from the original patient phenotype profile. To illustrate this approach, we selected a patient from the DEE11 cohort with a low number of phenotypes and a poor ranking, but still with sufficient annotations and without an extremely biased ranking, allowing the system to operate effectively. Specifically, we selected patient pp2173, which has five phenotype annotations: “Autism”, “Global developmental delay”, “Multifocal epileptiform discharges”, “Seizures” and “Delayed speech and language development,” and a normalized ranking of 0.01. These phenotypes are in the positions 3, 5, 7, and 23 of the phenotype prevalence list for DEE11 patient cohort shown in Table S3. Also, this patient lacks of the two more prevalent phenotypes “Epileptic Encephalopathy”, and “Intellectual disability”, the first is very specific an the second is very prevalent if we consider that the sum of all its forms reach the 45% of the cohort. The DEE11 cohort itself is enriched in phenotypes related to epilepsy, intellectual disability, seizures, autism, hypotonia and global developmental delay. In this context, the negative heatmap for pp2173 (Figure 4B) that list very different phenotypes found in the documents but that are not described for the patient is very coherent: the top 4 phenotypes are different forms of hypotonia which is the 9th phenotype in prevalence within the DEE11 cohort. The following phenotypes, “atrial septal defect” and “laryngeal cartilage malformation” are likely suggested due to descriptions of know complex syndromes that have multi-systemic affectation, including neurological system. Then we have several forms of macrocephaly with the 0.76% of the DEE11 cohort presenting this phenotype and 8.9% has its counterpart, microcephaly. Also, there are the suggestions of “Functional motor deficit” and “Impaired feeding ability” that could be related with the patient phenotypes. Finally, there is the case of “Hyponatremy” that is present in the 0.5% of the cohort and it is very related to seizures phenotypes.

### Biomedical text corpora: Exploratory data analysis

Initially, we calculated basic statistics on the documents retrieved from PubMed and PubMed Central. All available abstracts from PubMed and full-text articles from PubMed Central were downloaded via their FTP service, processed using the get_corpus_index module from the *py-semtools* library, and subsequently phenotypically annotated with the stEngine module bi-encoder only. The number of documents processed at each step is summarized in Table 2.

**Table 2:** Summary statistics of downloaded documents

	Abstracts	Articles
Total downloaded documents	36.555.430	6.132.543
With PMID/PMCID and Content	25.279.835	5.848.105
With phenotypic profile (stEngine - bi-encoder only)	10.903.007	5.029.690

Statistics on abstracts and full-text articles retrieved from PubMed and PubMed central. PMID refers to the PubMed identifier, and PMCID to the PubMed central identifier. “Content” indicates that the downloaded documents had digitized text available for analysis (excluding cases where abstracts are scanned images from printed articles). “With phenotypic profile” denotes documents that were successfully annotated with at least one phenotype in a meaningful way according to the proposed methodology.

From the large-scale processing of PubMed abstracts, most (69.2%) included full records with both *PMID* and *AbstractText* XML fields, allowing for their inclusion in downstream analyses. Nearly a third of these abstracts were successfully annotated with at least one phenotypic term, supported by a strong semantic similarity (≥ 0.7) between the text and the ontology. Similarly, for full-text articles retrieved from PubMed Central, most records (95.4%) included comprehensive metadata (with both PMID and PMCID records) and structured content. A substantial proportion of these articles also contained high-confidence phenotypic annotations (82%), indicating the suitability of full-text data for detailed semantic mapping.

To assess the variability and completeness of phenotypic information across different sources, we analyzed the distribution of phenotypic profile sizes for both PubMed abstracts and full-text articles after extracting their phenotypic annotations (Figure 3C). Additionally, we examined the profile size distribution for all OMIM diseases with at least one HPO term in the HPO annotations (phenotype.hpoa file, downloaded from https://hpo.jax.org/).

Results revealed notable differences between abstracts and full-text articles. Abstracts contained smaller phenotypic descriptions, with a median of two phenotypes per document and an interquartile range of three, meaning that half of the abstracts included between one and four phenotypic terms. In contrast, full-text articles provided substantially richer phenotypic information, with a median profile size of 12 phenotypes and an interquartile range of 19, indicating that 50% of these articles contained between five and 24 phenotypic terms. Notably, the distribution of phenotypic annotations in full-text articles showed substantial overlap with disease profiles, likely due to the greater textual content available in full texts, which increases the likelihood of capturing relevant phenotypic information.

To assess the difference in knowledge representation of both abstracts and full-text articles, we examined the publication year of all documents retrieved from PubMed and PubMed Central (Figure 3D). The median publication year was 2011 for abstracts and 2020 for full-text articles. The interquartile range spanned 18 years for abstracts, with 50% published between 2000 and 2018, whereas for full-text articles, it covered six years, with 50% published between 2016 and 2022. This means that although the full-text articles are the best candidates to extract meaningful information, the lack of access makes it necessary to use the abstracts corpora.

### Application to RASopathies

To demonstrate the applicability of our methodology, we performed an analysis centered on RASopathies. We selected these syndromes as part of our involvement in the EURAS (European network for neurodevelopmental RASopathies) consortium project, where we collaborate with leading clinicians specialized in these conditions. This allows us to seek expert feedback when needed and to provide insights that may be of interest to them, contributing to the practical application of our work. To this end, five RASopathies were tested: NF1, NFNS, Noonan, Costello, and CFC syndromes. As described in the Materials and Methods section, phenotypic annotation was applied to a large corpus of biomedical documents without prior disease-specific filtering. We then compared the phenotypic profiles derived from these documents to the established signatures of each RASopathy, assessed individually.

#### Measuring the top enrichment in RASopathies when PubMed is ranked using RASopathy HPO profiles

**Figure 5:**
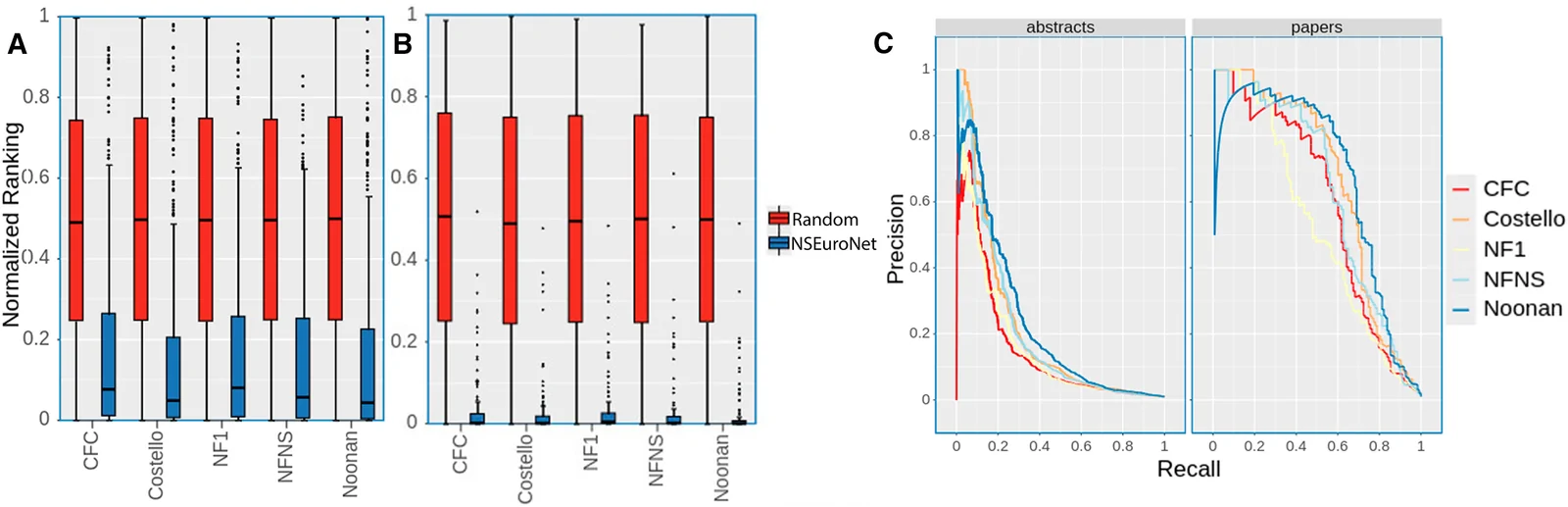
Retrieval performance and metrics for NSEuroNet RASopathies (A) Rankings for 498 abstracts indexed in NSEuroNet for different RASopathies and the addition of 49,800 random PubMed abstracts that were taken for each selected RASopathy. Error bars are defined as a 95% confidence interval. (B) Rankings for 123 open access articles indexed in NSEuroNet for different RASopathies and the addition of 12300 random PubMed articles that were taken for each selected RASopathy. Error bars are defined as a 95% confidence interval. (C) Precision-recall curves for the abstracts and open access articles indexed in NSEuroNet for each selected RASopathy.

To evaluate the enrichment of the top-ranked results for the selected RASopathies, we first compiled a list of expert-curated articles from the European Network on Noonan Syndrome and Related Disorders (NSEuroNet), available at https://nseuronet.com. This resource was created within the European project EJPRD19-210. In addition, we generated a set of randomly selected documents at a 100*x* ratio relative to the number of NSEuroNet articles. As shown in Figure 5A, the median ranking values for RASopathy-related abstracts range from 0.043 to 0.08, whereas the random document sets show median values between 0.49 and 0.499. This indicates that the top positions of the rankings are enriched in RASopathy-related documents. However, if we examine Figure 5B, the RASopathies documents are ranked with a median that ranges from 6.03e−4 to 4.42e−3 for each disease, whereas the median for the randomly selected articles ranges from 0.49 to 0.5. These results show that the top of the rankings is strongly enriched in RASopathy-related publications. This behavior is consistent across all selected RASopathies, including less frequently studied syndromes such as NFNS. The precision-recall curves in Figure 5C summarize the performance differences between abstracts and full-text articles and show that Noonan syndrome achieves stronger enrichment at the top of the rankings than NF1.

#### Phenotypic association of RASopathies across documents

**Figure 6:**
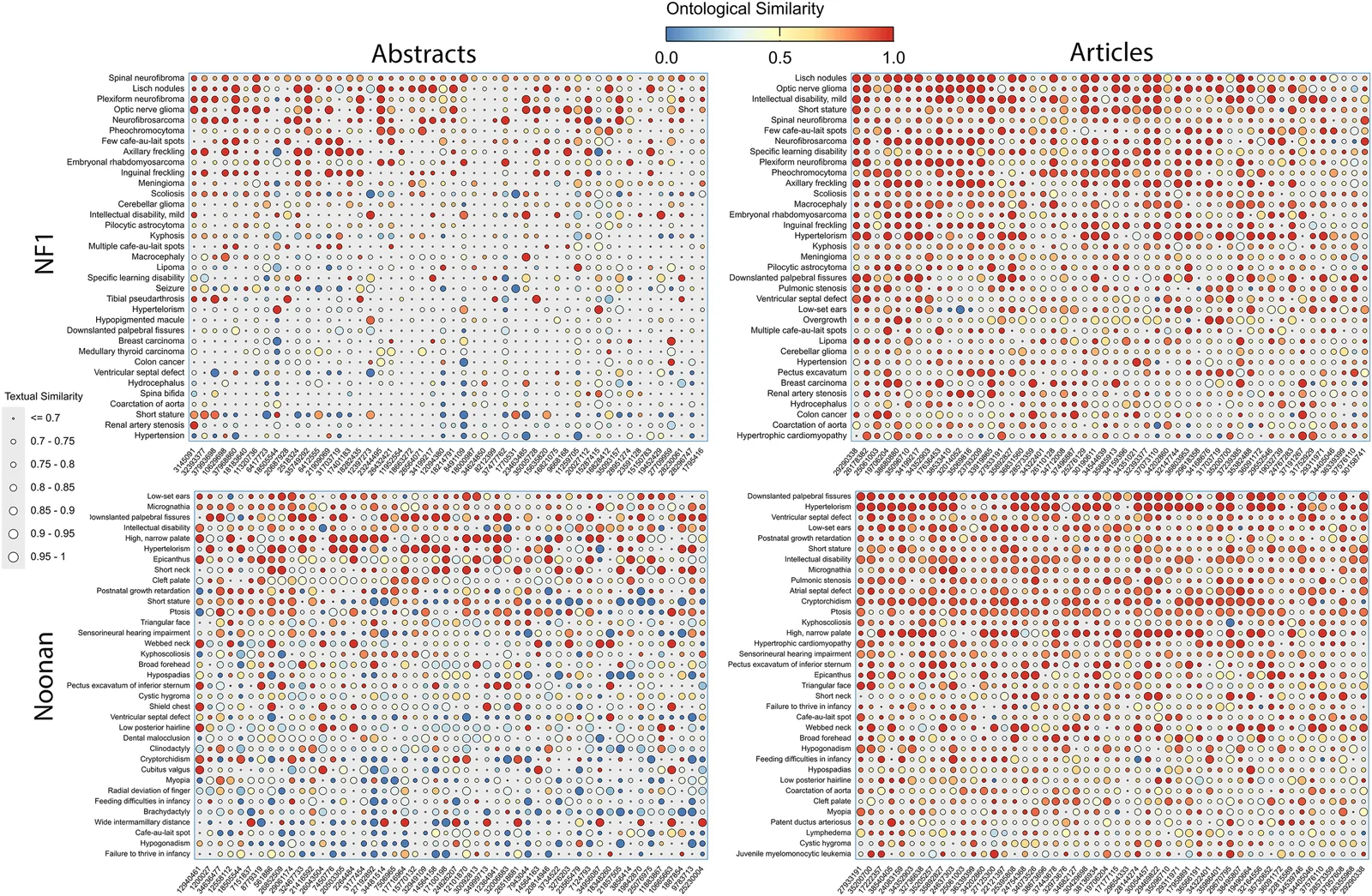
Phenotypic similarity heatmaps for NF1 and Noonan syndromes Ontological comparison between document-derived phenotypic profiles and those of selected RASopathies generated with py-semtools. Heatmaps show similarities between profiles from annotated PubMed abstracts and PubMed central full-text articles and the reference (syndrome) profiles of neurofibromatosis type 1 (NF1) and Noonan syndrome. Columns represent the document type (abstracts in the first column, full-text articles in the second column), while rows depict the syndrome (NF1 in the first row, Noonan in the second row). The PubMed IDs of the first 50 documents most closely related to the disease profile are shown on the X axis, ordered from highest to lowest ontological semantic similarity. The Y axis shows the names of each of the phenotypes described in the disease, ordered from highest to lowest by the mean ontological semantic similarity of the given term against each of the matched terms in the documents. Each cell color represents the ontological semantic similarity value, limited between 0 and 1, obtained by that disease phenotype against the matched term in that document (not shown in this figure). The size of the point reflects the textual semantic similarity, measured by cosine similarity, between the document sentence vector and the vector of the HPO phenotype that was mapped in that sentence of the document (not shown in the figure).

Figure 6 illustrates the phenotypic associations for NF1 and Noonan syndromes, where, for each heatmap, columns (X axis) represent a PubMed article (PMID) and rows (Y axis) correspond to phenotypes from the respective disease profiles. The raw data could be inspected in Tables S17, S18, S19, and S20. The titles of the documents associated with each PMID are included in Tables S7 and S8 for NF1, and in Tables S11 and S12 for Noonan syndrome, corresponding to abstracts and full-text articles, respectively.

These heatmaps reveal interesting patterns when comparing the use of abstracts versus full-text articles. For example, when analyzing NF1, we observe that phenotypes with the highest ontological similarity to the disease profile are annotated more frequently in the full-text articles (Figure 6, NF1 row, Articles column) than in the abstracts (Figure 6, NF1 row, Abstracts column). This is consistent with the previous analysis done to understand the variability and completeness of phenotypic information across different sources, where the distribution of phenotypic profiles778 is greater in full-text articles than abstracts.

Regarding textual similarity, in most of the suggested full-text articles, a large proportion of disease phenotypes match an annotated phenotype from the document with a textual similarity above the 0.7 similarity threshold used. This similarity threshold was chosen as it offers a good balance between accuracy and execution time, considering the large volume of data processed. In contrast, abstracts showed a significantly lower match rate, as many disease phenotypes could not be linked to ontologically similar terms within the documents, mainly because they did not reach the 0.7 similarity threshold. In both cases, “spinal neurofibroma,” “Lisch nodules” and “optic nerve glioma” are shown in the top five phenotypes.

For Noonan syndrome, a similar trend compared to NF1 is observed when using abstracts (Figure 6, Noonan row, Abstracts column) and full-text articles (6, Noonan row, Articles column). The analysis shows that the suggested abstracts for Noonan syndrome contain a slightly higher proportion of terms mapped to HPO terms with a textual similarity ≥ 0.7, even when some of these terms exhibit low ontological similarity to the disease phenotype. Consequently, the abstracts identified for Noonan syndrome display more comprehensive phenotypic profiles that allow a more thorough alignment with the disease characteristics than those retrieved for NF1. In these cases, we inspected the top six phenotypes to find three shared terms: “low-set ears,” “downslanted palpebral fissures” and “hypertelorism.”

Comparisons for NFNS, Costello, and CFC syndromes are shown in Figure S10, with document titles and corresponding PMIDs listed in Tables S9 and S10 for NFNS, S13–S14 for Costello, and S15–S16 for CFC. Overall, some resemblance is observed for suggested annotated articles among the three RASopathies (Figures S10B, S10D and S10F) in terms of textual and ontological similarity values. This pattern seems to hold in terms of the overall ontological similarity values among abstracts for these three RASopathies. However, the abstracts suggested for NFNS, in overall, they have a higher proportion of terms mapped to HPO entries with a textual similarity higher than 0.7, regardless of whether these terms have or not a high ontological similarity with the disease phenotype. The suggested full-text articles for these diseases appear more complete, as they contain a higher proportion of terms with strong textual similarity to disease phenotypes when compared to abstracts. Moreover, as observed in the case of NF1 and Noonan syndrome, these terms also show a closer alignment with the disease profiles, as most exhibit high ontological similarity.

It is noteworthy that a phenotype common to all diseases, “hypertelorism,” is represented in most abstracts and articles with a high value of ontological and textual similarity, except in the case of NF1 abstracts (Figure 6, Abstracts column, NF1 row). A similar case can be found with the term “downslanted palpebral fissures,” that is also found with relative high value of ontological and textual similarity for all syndromes and type of document, except for NF1 abstracts, and tends to co-occur with documents that also have the “hypertelorism” term annotated. The “short stature” term is also present in most cases with ontological similarity, but with variability in terms of textual similarity.

As an example of a phenotype that is specific for Noonan (Figure 6, Noonan row), Costello and CFC (Figures S10C–S10F) syndromes, “Micrognathia,” is represented in the vast majority of suggested abstracts and articles, but with lower values of ontological and textual similarity than in the case of “hypertelorism.”

Finally, an interesting case is found with some of the phenotypes common to NF1 (Figure 6, NF1 row), and NFNS (Figures S10A and S10B) syndromes, such as “neuroblastoma,” “plexiform neurofibroma” or “optical nerve glioma.” These phenotypes are highly prevalent for NF1 syndrome, appearing at the top of the list on the Y axis, in general with high textual and ontological similarity in the abstracts and suggested full-text articles. However, these same phenotypes, for NFNS syndrome, appear at the bottom of the list on the Y axis, with relatively lower textual and ontological similarities in both abstracts and suggested articles. Instead, another set of phenotypes, such as “hypertelorism,” “global developmental delay” or “posteriorly rotated ears,” among others, are more relevant for suggesting documents for this syndrome.

#### RASopathies clustering using full-text articles

**Figure 7:**
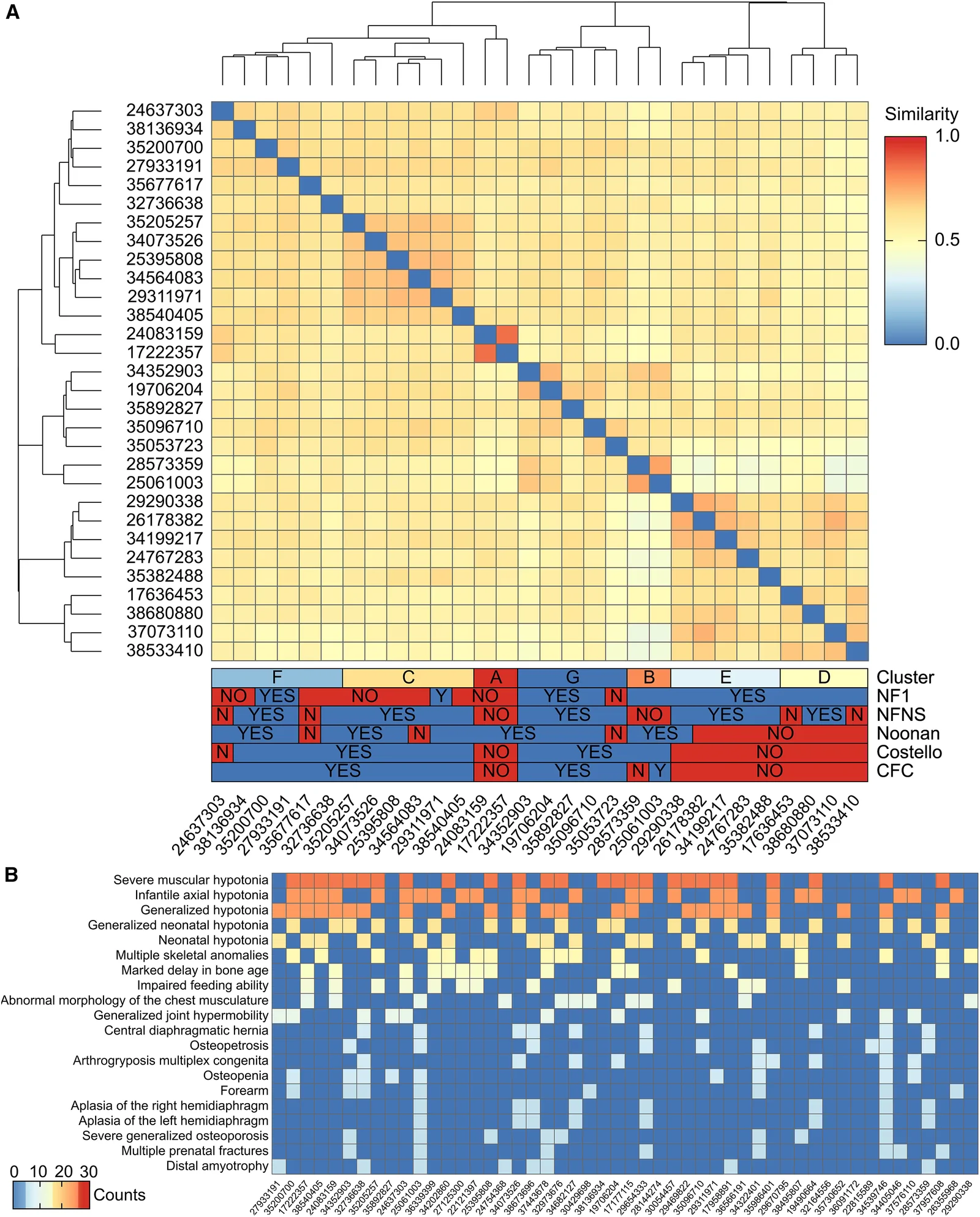
Semantic clustermap and Noonan-specific negative phenotypes (A) Clustermap showing the semantic clustering applied to the top 10 most similar full-text articles for neurofibromatosis type 1 (NF1), Neurofibromatosis-Noonan (NFNS), Noonan, Costello and Cardiofaciocutaneous (CFC) syndromes. The clustering is based on the phenotypic profiles associated with each document, highlighting the semantic relationships and grouping patterns among the articles relevant to each syndrome. The overlays at the bottom show clusters associated with each document (Cluster), and for each syndrome whether a given document was in the top 50 ranked suggestions (NF1, NFNS, Noonan, Costello, and CFC). (B) Representation of the most frequent terms across the top 50 recommended full-text articles that are not similar to Noonan-related phenotypes (called “negative terms”). The X axis represents the PMIDs of the top 50 recommended articles, ranked by their relevance to Noonan syndrome (in the same order of Figure 6, Noonan row, articles column). The Y axis lists the phenotypes annotated in these documents that are not ontologically related to the disease profile, sorted by their frequency within the top 50 articles. For clarity, only the 20 most frequent terms are displayed. The color intensity reflects the number of times each phenotype was annotated across the top 50 articles.

Based on the results obtained for the full-text articles, we performed a clustering procedure with *py-semtools*. For each RASopathy studied, we selected the top ten PMIDs of the articles with the highest similarity score. This resulted in a combined set of 30 unique articles, which were subsequently ranked based on their average position across the five RASopathies. Table S4 lists the titles of these articles, along with their ranking within the top 50 for each syndrome (if applicable). The clustering analysis is based on the document-associated phenotypic profiles using the semantic clustering module of py-semtools, applying hierarchical clustering to a semantic ontology similarity matrix of the selected documents. The compilation and clustering of the most relevant articles identified by the proposed methodology for the different RASopathies enable the delineation of a knowledge domain for the set of diseases studied and can be extended to other disease contexts. A closer examination of the results in Figure 7A illustrates the relationships among the documents based on their phenotypic content, offering insights into how information relevant to the selected syndromes is structured and interconnected.

Figure 7A shows that the 30 articles are grouped into seven distinct clusters (A–G). As shown in its dendrogram, there is a major split separating clusters E and D from the rest. Notably, clusters E and D are predominantly associated with NF1 and NFNS syndromes and barely mention the other RASopathies. In contrast, clusters A, B, C, F, and G include articles relevant to different groups of RASopathies. Specifically, cluster G encompasses documents spanning all syndromes, while most articles in cluster C and F are primarily linked to NFNS, Noonan, Costello, and CFC syndromes, with articles in cluster C having higher internal phenotypic similarity. Finally, cluster B is comprised of two articles common to all RASopathies but NFNS, and cluster A contains two highly similar articles that are unique to Noonan syndrome.

These clustering results are in line with similarities shown from disease phenotypic profile comparisons of the RASopathies in Table S6. NF1 and NFNS syndromes exhibit the highest similarity, which is consistent with the clustering pattern seen in clusters E and D. Both Costello and CFC also exhibit high similarity, and to a lesser extent they also have a good similarity with Noonan, clustering together as the complement to NF1 and NFNS syndromes in clusters E and D. However, NFNS syndrome still exhibits higher similarity with Noonan, Costello, and CFC than NF1, being consistent with the patterns seen in clusters C and F.

#### Assessing clinical relevance using negative heatmaps

To mitigate the risk of false positives due to model over-annotation and to assess the clinical relevance of overlooked phenotypes, we developed a complementary approach referred to as a negative heatmap. This procedure initially includes all annotated phenotypes from the top 50 full-text articles. Each phenotype is compared ontologically to the disease profile, retaining only those with a maximum ontological similarity score of 0.2 or less. This approach captures phenotypes that could represent potentially relevant phenotypes not yet observed in the initial patient characterization.

An example of the negative heatmap specifically for Noonan syndrome is shown in Figure 7B. We observe that certain phenotypes appear in up to 26 of the 50 suggested full-text articles, indicating that they are relatively prevalent despite not being ontologically close to any terms in the disease profile. The five most frequent terms are related to “hypotonia,” mentioning three of them the word “neonatal/infantile.” Moreover, other terms with notable frequency are “multiple skeletal anomalies,” “marked delay in bone age” and “impaired feeding ability.”

For NF1 syndrome (Figure S11A) the three most frequent terms are “multiple skeletal anomalies,” “osteopetrosis” and “generalized joint hypermobility,” all related to the musculoskeletal system. For NFNS syndrome (Figure S11B), the most frequent terms are “microgastria,” “multiple skeletal anomalies” and “reduced circulating growth hormone concentration,” each referring to distinct physiological or anatomical conditions.

Finally, for Costello (Figure S11C) and CFC (Figure S11D) syndromes, “multiple skeletal anomalies” also ranks among the top three phenotypes in both cases, highlighting its high prevalence as a term shared across all five RASopathies. “congenital hypothyroidism” term also ranks among the top for both syndromes. Additionally, as in the case of NFNS syndrome, “reduced circulating growth hormone concentration,” also appears in Costello syndrome, while in CFC syndrome, “impaired feeding ability,” emerges as the most prevalent phenotype. Furthermore, the term “Thrombocytosis” also appears across different RASopathies, except for NFNS and Noonan syndromes.

## Discussion

The correct diagnosis of rare diseases is a challenge for clinicians, not only because of their low prevalence but also owing to their phenotypic heterogeneity and frequent symptom overlap with other common disorders.^6^ In many regions, limited awareness among healthcare professionals and uneven access to genetic medicine specialists exacerbate the problem: some countries lack formal training programs or certified practitioners in clinical genetics.^45^ As a result, patients and families often endure years of diagnostic odysseys, undergoing repeated tests and consultations before arriving at a definitive diagnosis.^46^

Misdiagnosis or delayed diagnosis can have profound consequences: it may delay appropriate therapy, lead to unnecessary or invasive procedures, and impose considerable emotional and financial burdens on families.^47^ Moreover, the scarcity of centralized registries and standardized phenotyping protocols further impedes the recognition of characteristic clinical patterns.^48^ Improving the systematic capture and interpretation of phenotypic features is therefore essential to shorten diagnostic timelines, facilitate early intervention, and connect patients with specialized care and support networks.^48^^,^^49^

In this context, the development of computational tools and frameworks that can mine and interpret large bodies of biomedical literature and harmonize those findings with standardized ontologies offers a powerful means to improve clinical expertise to aid in the diagnosis of human genetic diseases.^50^^,^^51^ Our framework addresses this need by prioritizing scientific articles most relevant to a disease or a combination of phenotypic features annotated using the HPO. Using several different algorithms, we can relate both HPO terms (queries) and biomedical texts from PubMed abstracts and PubMed central full-text articles (corpus). This enables the computation of ontological semantic similarity between patient-specific phenotypic profiles and biomedical literature. The result is a ranked list of scientific publications that are most closely related to the clinical features observed in a given patient, helping clinicians and researchers to identify potential diagnoses, understand disease mechanisms, and explore relevant therapeutic avenues more efficiently.

Biomedical ontologies, such as the HPO,^26^ provide a standardized and hierarchical representation of phenotypic traits, allowing for consistent annotation and comparison across datasets. Their structure not only captures specific symptoms but also their semantic relationships, making them ideal for organizing clinical information and supporting automated reasoning. In our study, the use of HPO terms enabled us to annotate large volumes of text with precise phenotypic labels, which in turn facilitated the comparison between literature-derived profiles and curated disease definitions. This ontology-based framework is particularly valuable for rare diseases, where subtle or compound symptoms often hold the key to correct diagnosis.

To capture the complex and often implicit associations between diseases and phenotypes in biomedical literature, we show that sentence embedding models provide a fast and reliable approach for identifying biomedical concepts. These models enabled us to go beyond keyword-based approaches, allowing the identification of relevant text segments even when phenotypic terms appeared in paraphrased or context-dependent forms. By applying these embeddings to large-scale corpora, comprising both abstracts and full-text articles, we were able to rank phenotype-disease associations with high semantic relevance, as reflected in our “OMIM-Ehrhart,” “OMIM-DO” and “MPS” gold-standard benchmarking results. The observed differences in model performance across workflows (DDP vs. IPDP) and document types underscore the importance of both corpus characteristics and model architecture. The three datasets show that our platform can work with a large variety of diseases as listed in OMIM, and it can retrieve relevant articles independently of the disease prevalence. In fact, the MPS dataset shows that our approach can deal with real patient data and that the patient phenotype characterization is a key factor to get the best results possible without tool.

The use of the “OMIM-Ehrhart” and “OMIM-DO” gold-standard datasets was essential for validating our approach. By comparing our ranked predictions against these independently curated disease-publication associations, we consistently demonstrated high precision across both DDP and IPDP workflows, with correct PMID-disease relationships appearing at the top of the ranked lists, which confirms that our ontology-guided, embedding-based approach can retrieve syndrome-specific publications with good balance between precision and computational time. Additionally, we evaluated several text analysis strategies such as TF/IDF, bm25, sentence transformer only, Sentence Transformer with re-rank, and OntoGPT and found that the pre-trained “all-mpnet-base-v2” model from sentence transformers offered superior performance in retrieval tasks considering both precision and computational time.

The tool developed in this work, *py-semtools*, integrates ontology-guided annotation, document text scoring, and a scalable pipeline for processing biomedical literature. One of the main advantages of this system is its applicability across disease domains without requiring prior filtering or disease-specific training. When applied to a set of RASopathies, including NF1, NFNS, Noonan syndrome, Costello syndrome, and CFC syndrome, our methodology effectively prioritized relevant literature for each condition, as demonstrated by the analysis shown in Figure 5.

In general, we observed a lower coverage of disease phenotypic profiles in abstracts compared to full-text articles, which we expected would negatively affect the relevance of the documents retrieved for a given disease. Surprisingly, this trend did not hold in the case of NF1. Among the top 50 documents retrieved for NF1, 28 abstracts explicitly include the term NF in their titles (Table S7), whereas only 14 full-text articles do so (Table S8). This finding is particularly striking, and a closer examination of the phenotypic annotations in these NF1 abstracts reveals the presence of distinctive phenotypes, such as “spinal neurofibroma,” “plexiform neurofibroma,” and “neurofibrosarcoma,” which are highly specific to NF1 and reflect the traits that define the syndrome. Moreover, they are etymologically embedded in its name (NF). This unique feature allows the model to retrieve highly relevant documents, even from phenotype-scarce abstracts. In this sense, NF1 functions as an “autodescriptive” syndrome, where the disease name encapsulates key phenotypes, simplifying its recognition in the literature.

In contrast, this behavior does not generalize to other RASopathies that lack similarly descriptive naming; the disease phenotypes are needed to describe these diseases. While abstracts can capture common or headline phenotypes, they frequently omit deeper or rarer manifestations that are critical for differentiating closely related syndromes. Consequently, full-text processing provides a more comprehensive basis for phenotype-based literature mining in rare disease contexts. In addition, despite its greater computational demand, the system maintained robust performance, particularly when evaluated over broader ranking thresholds. Importantly, the approach is modular and adaptable: thresholds, algorithms, models, and ontologies can be adjusted to suit different clinical or research needs. For syndromes such as Noonan, Costello, and CFC, the expected pattern holds: a greater number of relevant full-text articles than abstracts mention the disease explicitly in their titles (Tables S11, S12, S13, S14, S15, and S16, respectively). This highlights the added value of full-text analysis for diseases whose phenotypic identity is more dispersed or less clearly reflected in their nomenclature.

Interestingly, despite the lack of explicit disease name mentions in many documents, our approach was still able to effectively prioritize relevant literature for syndromes, such as Noonan. Unlike NF1, whose distinctive phenotypes and self-descriptive name facilitate recognition, Noonan syndrome does not have hallmark terms embedded in its title, nor a set of uniquely defining phenotypes that consistently appear in the text. Nonetheless, our system successfully retrieved top-ranked documents for Noonan based uniquely on the semantic similarity of associated phenotypes. This suggests that our methodology can capture phenotypic patterns in documents even when disease names are absent. Such capacity is especially relevant in rare disease contexts, where terminologies may vary, and direct disease mentions can be sparse, reinforcing the value of phenotype-based semantic search.

Our clustering analysis of the top-ranked articles for the five RASopathies revealed coherent groupings that give both shared and distinct phenotypic themes. One cluster (Cluster G) contained articles highly relevant to all syndromes, reflecting broad discussions of multiple RASopathies within single publications, often in the context of overlapping cardiovascular or developmental features. For example, one of the top articles includes mentions for all RASopathies, except for NFNS, in its body text.^52^

A closely related cluster (Cluster F) comprised articles focused on “cardiocutaneous” and cardiomyopathic manifestations, again spanning several RASopathies but with variable syndrome mentions in the text. For example, this cluster includes two interesting publications that mention Noonan, Costello, and CFC in their body text. One of them is a review about the genetics of inherited cardiocutaneous syndromes,^53^ and another one, more recent, describes the clinical importance of determining the etiology of pediatric cardiomyopathy in rare syndromes.^54^

In contrast, another cluster (Cluster C) grouped articles that, although frequently retrieved for Noonan, NFNS, Costello, and CFC syndromes, centered on oral and craniofacial developmental anomalies without explicit mentions of these RASopathies in the text corpus.^55^^,^^56^ For example, one of these publications describes different chromosomal and multifactorial disorders with oral manifestations, such as the velocardiofacial syndrome, a rare disease characterized by specific craniofacial features.^55^ This fact suggests that our phenotype-based approach can surface relevant literature even when disease names are not directly invoked.

Clusters D and E were more disease-specific, containing articles predominantly associated with NF1 and NFNS. These publications routinely mentioned “neurofibromatosis,” “Noonan,” or the *NF1* gene in their titles and bodies, underscoring genuine NF1-NFNS overlaps in clinical presentation and molecular etiology. For example, one of the articles describes a study about the clinical characteristics of patients with missense mutations in the *NF1* gene, affecting the amino acid residue p.Arg1809, and which findings reveal a high incidence of features commonly associated with Noonan syndrome, particularly short stature and pulmonic stenosis, in this subgroup of patients with NF1.^57^

Finally, the most tightly defined cluster (Cluster A) comprised articles explicitly focused on Noonan syndrome, with direct title mentions and highly similar descriptive passages. The close textual and phenotypic alignment within this cluster highlights the ability of our method to pinpoint literature that not only matches a phenotype of the syndrome, but also discusses it in detail. For example, one of its articles mentions “Noonan” in its title, and it describes what Noonan syndrome is, its clinical features, prevalence, associated genes, and how early diagnosis can improve the life expectancy of these patients.^58^

Together, these clusters demonstrate that phenotype-based semantic clustering can capture both broad, cross-syndrome themes and highly specific disease contexts. This stratification can guide researchers and clinicians to the most pertinent literature for each RASopathy, whether their interest lies in shared pathophysiological mechanisms or in syndrome-specific manifestations.

The method also successfully recovered phenotype profiles that closely matched curated disease signatures, even when the phenotypes were dispersed across diverse articles. This illustrates the potential of our methodology to assist in real-world diagnostic scenarios, where the phenotypic presentation may be atypical or poorly documented.

However, as illustrated in Figure 2, the annotation process is not perfect and may introduce false positives, leading to over-annotation of documents. Although applying more stringent textual semantic similarity thresholds can help mitigate this issue, it simultaneously increases the risk of false negatives. This trade-off, though difficult to address directly, can be accounted for with the “negative heatmap” approach included in the tool. They points out phenotypic terms prevalent in top-ranked documents but unrelated (ontologically distant) to the disease profile. Using the “MPS” dataset, we showed that patient phenotype characterization can be significantly enhanced through the negative heatmap feature, which assists clinicians in completing the characterization process.

In the RASopathy analyses, these heatmaps revealed both shared and syndrome-specific “off-target” phenotypes, some appearing across all RASopathies (e.g., multiple skeletal anomalies), others unique to a subset of the syndromes (e.g., impaired feeding ability in CFC and Noonan syndromes). This visualization aids clinicians and researchers in filtering out spurious associations, yet it is not without limitations. In particular, strict similarity thresholds that minimize false positives can also exclude legitimately rare or unique phenotypes (false negatives). As such, negative heatmaps should be used in conjunction with expert review, and similarity thresholds may require fine-tuning for different disease domains.

Without pretending to be an exhaustive review, we would like to focus on the terms that appeared in the “negative heatmaps” for Noonan syndrome (Figure 7B). We observed that the top five terms were related to “hypotonia,” with three of them also mentioning “infantile/neonatal.” Accessing the main text of the third and fifth articles,^58^^,^^59^ which mention Noonan in their title, and the sentence “the muscular hypotony that is often present in early childhood” is included in both articles. The sentence “She had mild muscular hypotonia” is also included in the main text of one of the articles.^60^ However, there are also other articles that mention “Noonan” in their title that do not have any of the hypotonia-related terms annotated. Nevertheless, it is a possible case of a phenotype observed in patients described in scientific publications that is not considered in the phenotypic profile of Noonan syndrome as is described in OMIM, which reflects the ability of our methodology to suggest phenotypes associated with the disease not included in the profile.

In clinical practice, this framework may be particularly valuable in cases of uncertain or complex diagnoses, where the phenotypic characterization of a patient is incomplete or difficult to establish. Starting from an initial set of HPO terms, the system retrieves and ranks publications associated with similar phenotypic profiles, enabling clinicians to explore potential diagnostic hypotheses. A key feature of the framework is the phenotype negative heatmap, which highlights phenotypic features frequently reported in the retrieved literature but absent from the current patient profile. These features may correspond to clinical signs that were not initially documented or that require specific examinations to detect. By revealing potentially missing phenotypic information, the negative heatmap can guide clinicians in refining the characterization of the patient and in identifying additional clinical evaluations that may be relevant. This capability supports an iterative diagnostic process in which the phenotypic profile can be progressively refined and reanalyzed against the biomedical literature. In this way, the approach not only facilitates systematic exploration of the literature but also promotes more comprehensive and accurate phenotyping in challenging diagnostic scenarios.

Finally, we developed a web portal (https://www.clinsysbiolab.es/) that allows users to input a set of HPO terms derived from a real patient (e.g., from an electronic health record) and perform a document search within the platform. As described previously, the first step involves examining the negative heatmap to identify additional phenotypes associated with the patient's disease. This step may help to identify overlooked characteristics, potentially suggesting new tests for the patient. Next, the user can verify whether there is a strong signal in phenotypes unrelated to the patient's clinical presentation, allowing assessment of whether the tool has ranked publications irrelevant to the case. Once the phenotypic characterization is performed and the negative heatmap verified, the user can inspect the listed documents and the relevance of phenotypes within them to identify publications that best describe the patient profile, providing clues for possible diagnosis and treatment. This approach is particularly valuable during early stages of patient evaluation, when limited clinical information is available, and genomic data may be absent, as it accelerates diagnosis by comparing patient phenotypes against the entire biomedical literature. Furthermore, since the platform is delivered as a Python library, it can be easily integrated into any patient analysis pipeline and is designed to scale from small hospital environments with limited computational resources to large infrastructures such as dedicated computing clusters.

Overall, this study demonstrates how combining ontologies, semantic text search, and large-scale literature mining can support more precise phenotypic profiling, with direct implications for improving rare disease diagnosis. While there are still limitations, such as dependence on the availability and quality of literature, and the potential for noise in semantic predictions, the methodology provides a scalable and effective strategy for integrating textual evidence into clinical research. As biomedical literature continues to grow, tools like *py-semtools* may play an increasingly important role in bridging the gap between unstructured data and structured clinical knowledge, ultimately contributing to better diagnostic outcomes in the field of rare diseases.

### Limitations of the study

The proposed approach yielded favorable results on the benchmarking datasets and in the RASopathies case study. However, as noted earlier, the annotation process may produce false positives. For this reason, the negative heatmap described in the Methods section can be used to differentiate between false positives and phenotypes not previously associated with the patient or disease, facilitating a careful review by an expert. Thus, it remains an open question how to enhance the ability of the tool to reduce false positives without increasing false negatives. Given that the text embedding model used by our tool was trained on a general-purpose text corpus, a potential future approach would be to fine-tune it with biomedical data, thereby improving its robustness.

Another limitation arises when HPO-annotated articles are ranked based on ontological similarity using the full list of annotated phenotypes, as this may also generate false positives. For example, the article PMID: 28895939 appears among the top 50 suggested articles for Noonan syndrome, although it discusses a series of neurogenetic disorders. This article was suggested due to phenotypic similarity to Noonan syndrome, since the annotated phenotypes represent the union of all described syndromes, reflecting the broader phenotypic spectrum of RASopathies. Addressing this will require future work to associate each phenotype with its corresponding disease entity within the article.

In addition to text-based phenotypic descriptions, emerging image-based phenotyping approaches may further enrich rare disease characterization. Tools such as Face2Gene^61^ and Eye2Gene^62^ could be useful to extract phenotypic signals directly from facial or retinal images, enabling the identification of patterns that may be difficult to capture through structured phenotype terms alone. Our current framework focuses on phenotype information encoded using HPO terms and therefore does not yet incorporate imaging-derived phenotypic features. However, integrating such modalities represents a promising direction for future work, as combining structured phenotypes with image-derived features could potentially improve literature retrieval and disease prioritization in complex or partially characterized cases.

## Resource availability

### Lead contact

Requests for further information and resources should be directed to and will be fulfilled by the lead contact, Pedro Seoane Zonjic (seoanezonjic@uma.es).

### Materials availability

This study did not generate new materials.

### Data and code availability

• The Human Phenotype Ontology (HPO), whose terms were used to phenotypically characterize the diseases analyzed in this work and to annotate PubMed documents, is available on its official website (https://hpo.jax.org/) in the data/ontology section, file hpo.obo. All PubMed abstracts used in this work are available through the NCBI FTP service https://ftp.ncbi.nlm.nih.gov/pubmed, and https://ftp.ncbi.nlm.nih.gov/pub/pmc/ for open-access articles. Datasets used as gold standard can be found at Ehrhart et al.^63^ or in the Disease Ontology, downloaded from https://obofoundry.org/ontology/doid.html. Detailed information on how to download and process these data is provided in the STAR Methods section.
• The tool developed in this study is available as an open-source Python package, *py-semtools*, on the Python Package Index (PyPI), and at the Zenodo public repository with https://doi.org/10.5281/zenodo.21534870. The code developed for the analysis of the benchmark datasets and application case with RASopathies is available at the Zenodo public repository with https://doi.org/10.5281/zenodo.21534320 in the GitHub repository https://github.com/seoanezonjic/SemanticAnalysisPubmed.
• Any additional information required to reanalyze the data reported in this article is available from the lead contact upon request.

## Acknowledgments

This work is supported by funds from Spanish Ministry of Economy and Competitiveness [PID2019-108096RB-C21 and PID2022-140047OB-C21, PID2022-140047OB-C22]; the Institute of Health Carlos III (project IMPaCT-Data, exp. IMP/00019), co-funded by the European Union, European Regional Development Fund (ERDF, “A way to make Europe”); and the European Union through the project EURAS (Horizon Europe HORIZON-HLTH-2022-DISEASE-06, Project ID: 101080580) to J.A.G.R. The EURAS project receives funding from the European Union’s Horizon Europe Research and Innovation Programme under grant agreement no. 101080580 (HORIZON-HLTH-2022-DISEASE-06). Funded by the European Union. Views and opinions expressed are however those of the author(s) only and do not necessarily reflect those of the European Union or the Health and Digital Executive Agency. Neither the European Union nor the granting authority can be held responsible for them. Biomedicine research projects [PI-0075-2017 and PEER-0019-2020] are funded by 10.13039/501100015757Fundación Progreso y Salud. E.R. hold a research grant from the Instituto de Investigación Biomédica de Málaga (IBIMA-Plataforma BIONAND) [PI RARE 24-03]. J.P.G. is a predoctoral researcher from “ayudas para contratos predoctorales para la formación de doctores” (PRE2022/000510) supported by the Ministerio de Ciencia, Innovación y Universidades. F.G.C. is a predoctoral researcher from “ayudas para contratos predoctorales para la Formación del Profesorado Universitario” (FPU21/01449) supported by the Ministerio de Ciencia, Innovación y Universidades. The “CIBER de Enfermedades Raras” is an initiative from the ISCIII (Spain). The funders had no role in the study design, data collection and analysis, decision to publish or preparation of the manuscript. The authors thank the Supercomputing and Bioinnovation Center (SCBI) of the University of Málaga for their provision of computational resources and technical support (http://www.scbi.uma.es/site). We thank to Junta de Andalucía its support to PAIDI BIO-267 group.

## Author contributions

Conceptualization, J.A.G.R., J.P.G., and P.S.Z.; methodology, J.A.G.R., J.P.G., F.G.C., and P.S.Z.; software, J.P.G., F.G.C., and P.S.Z.; formal analysis, J.A.G.R., J.P.G., P.S.Z., and E.R.; writing – original draft, P.S.Z., J.P.G., and E.R.; writing – review and editing, all authors; funding acquisition, J.A.G. and P.S.Z.; supervision, J.A.G. and P.S.Z.

## Declaration of interests

The authors declare no competing interests.

## Declaration of generative AI and AI-assisted technologies in the writing process

During the preparation of this work, the authors used DeepL exclusively for the purpose of editing specific sections where there were uncertainties regarding the language. After using this tool/service, the authors reviewed and edited the content as needed and take full responsibility for the content of the publication.

## STAR★Methods

### Key resources table

**Table:** 

REAGENT or RESOURCE	SOURCE	IDENTIFIER
**Deposited data**		

HPO	HPO, 2025/02/25	https://hpo.jax.org/data/ontology (OBO file)
PubMed Abstracts	PubMed, 2024/04/29	https://ftp.ncbi.nlm.nih.gov/pubmed
PubMed Articles	PubMed, 2024/05/21	https://ftp.ncbi.nlm.nih.gov/pub/pmc/
Ehrhart gold-standard dataset	Ehrhart et al.^63^	https://figshare.com/articles/dataset/Gene-RD-Provenance_V2/7718537/1?file=14367506
Disease Ontology (DO) gold-standard dataset	DOID, 2025/02/25	https://obofoundry.org/ontology/doid.html (OBO file)
Monarch Phenopacket Store (MPS) gold-standard dataset	Monarch repository, 2026/02/26	https://github.com/monarch-initiative/phenopacket-store/releases

**Software and algorithms**		

py-semtools	This article	Available as a Python library, called py-semtools, at PyPI or at https://doi.org/10.5281/zenodo.21534870
Benchmarking workflow	This article	https://doi.org/10.5281/zenodo.21534320

### Method details

#### Documents ranking with fmEngine module

To enable the workflow described in this study to be used in environments with limited computational resources or where shorter execution times are required, we implemented the fmEngine module. Unlike the deep-learning-based modules (such as stEngine and llmEngine), fmEngine relies on classical text-mining models based on term frequency, which are typically optimized for multithreading on multi-CPU systems. We have tested this approach and found it particularly well-suited for scenarios with limited computational resources, providing an efficient and practical alternative to more demanding methods such as language models. Specifically, we adapted two widely used classical methods: Term Frequency-Inverse Document Frequency (TF-IDF) and Best Matching 25 (BM25), to operate within a common interface shared with the other modules in the *py-semtools* library, ensuring seamless integration and consistent usage across the framework.

TF-IDF is a foundational statistical measure in information retrieval, used to assess the importance of a word within a document relative to a larger collection (corpus). It combines two components: Term Frequency (TF), which counts how often a word appears in a specific document, and Inverse Document Frequency (IDF), which measures how rare or common that word is across the entire corpus. A word is thus considered highly relevant to a document if it occurs frequently within that document but infrequently across the rest of the corpus.^40^ For its part, BM25 is a more advanced ranking function, widely regarded as the gold-standard for traditional keyword-based search and the default scoring algorithm in major search engines. BM25 introduces TF saturation, where the relevance score increases quickly with the first few occurrences of a word but eventually levels off. In addition, BM25 incorporates document length normalization, ensuring that both shorter and longer documents are fairly evaluated according to their true density of relevant information.^41^

For the implementation of TF-IDF, we used functions from Python’s sklearn library. Specifically, the “TfidfVectorizer” class vectorizes the text fragments from both query and corpus sets, and the linear_kernel function calculates the similarities between the two sets. For BM25, we used the bm25s library,^64^ which provides a fast, low-dependency and low-memory implementation. The “tokenize”, “index” and “retrieve” functions of the “BM25” class were used for tokenization, vectorization and similarity computation.

Since the TfidfVectorizer implementation normalizes the resulting vectors, the “linear_kernel” function we use in this implementation, which computes the dot product of the vectors, is equivalent to a cosine similarity. Therefore, the similarity values obtained for the TF-IDF implementation are bounded between 0 and 1. In the case of BM25, the similarity score differs from that of TF-IDF, since instead of treating texts as vectors in a space and measuring the angle between them, BM25 estimates the probability that a document is relevant to a given query. The score calculated by this algorithm has a lower bound of 0, but no upper bound.

The operation of fmEngine is similar to that of the stEngine and llmEngine modules (see relevant subsections in the “STAR Methods” section). The module accepts a set of queries, a text corpus, a score threshold, a top-K value and the chosen model (currently TF-IDF or BM25, with the possibility of extending to other methods). It calculates the scores between the queries and the corpus, returning, depending on the chosen level of granularity (document, paragraph or sentence) the top-K queries that exceed the score threshold for each unit of text.

#### Documents embedding and textual semantic similarity calculation with the stEngine module

The stEngine module is built upon the Sentence Transformers library (v3.2),^25^ which provides a range of models derived from Bidirectional Encoder Representations from Transformers (BERT), also known as bi-encoder models.^20^ These lightweight computational models transform text fragments into high-dimensional vector embeddings that capture their semantic meaning. By representing text in this way, semantic similarities between different fragments can be quantified and compared,^65^ making this approach a powerful tool for natural language understanding tasks. Furthermore, these models are computationally efficient, allowing the processing of very large text corpora, such as PubMed, on limited resources within a reasonable time-frame.^66^

Sentence Transformers offers various embedding models with differing dimensionalities. Generally, models trained with higher-dimensional embeddings achieve greater accuracy, but this comes at the cost of increased training and inference times. Considering this trade-off and the absence of our own model with labeled data, we have used the pre-trained “all-mpnet-base-v2” model (https://huggingface.co/sentence-transformers/all-mpnet-base-v2), which has 768 embedding dimensions and, according to their authors, provide the best results in embedding and semantic search tasks in 14 and six datasets tested, respectively, of the several models trained by them.

As previously described, these embeddings are trained to preserve the semantic similarity between related text fragments in a given language.^67^ This allows the use of similarity functions to compare pairs of vectors generated by these models, enabling the determination of how similar the text fragments represented by the vectors are.^25^

With stEngine, different embedding models from the Sentence Transformers library can be downloaded and used to embed both query and corpus texts, generating their respective vectors. For each document (or sentence if the document has been fragmented) of the corpus, the module calculates the text semantic similarity with all available queries using cosine similarity. It then identifies the top-K pairs with the highest similarity and filters the results to return only those pairs that exceed a predefined significance threshold.

In addition, another family of models, known as “cross-encoders”^42^ is also designed to assess the similarity between two text fragments. The key difference lies in how the texts are processed: in a bi-encoder model, the query and document are processed independently to generate separate vector embeddings. This allows for faster computations; however, because the texts do not interact during embedding, bi-encoders may overlook deeper contextual relationships and subtle nuances between the two fragments.

In contrast to bi-encoder models, cross-encoder models process the query and the document simultaneously. This design allows the attention mechanism in the model to compare each word in the query directly with each word in the document, capturing deeper contextual relationships and subtle nuances. Although this interaction improves accuracy, it requires higher computational resources and longer processing times.

To balance efficiency and precision, a common approach in the field is the “retrieve-and-rerank” strategy. We have implemented a fast bi-encoder that first searches a large text corpus to retrieve a manageable top-K set of potentially relevant documents. A cross-encoder is then applied to this reduced set to re-rank the results, ensuring more accurate prioritization. The stEngine module implements this “retrieve-and-rerank” strategy to refine document annotation with ontology terms. For these experiments, the “cross-encoder/ms-marco-MiniLM-L6-v2” model (https://huggingface.co/cross-encoder/ms-marco-MiniLM-L6-v2) was used as the cross-encoder, while the “all-mpnet-base-v2” model was retained as the bi-encoder. This configuration provides a practical balance between computational efficiency and ranking accuracy, allowing precise semantic comparisons without excessive resource requirements.

To obtain similarity scores with bounded limits, as in the case of bi-encoder models (which use cosine similarity), we use the sigmoid activation function when employing cross-encoder models, so that the resulting similarity score is constrained to a range between 0 and 1.

#### Ontology term extraction with llmEngine module

The llmEngine module provides an interface for extracting biomedical terms from text corpora using large language models at full capacity. The current implementation integrates the open-source Python package OntoGPT,^44^ which extracts structured semantic information from unstructured text by grounding it in established knowledge bases.

OntoGPT relies on its Structured Prompt Interrogation and Recursive Extraction of Semantics (SPIRES) engine, which recursively generates prompts for a language model based on a user-defined data schema. The extracted information is then mapped to ontology identifiers, reducing ambiguities and hallucinations.^43^ This approach differs methodologically from the embedding-based strategy of stEngine but serves a similar purpose: converting free text into ontology-aligned structured data.

OntoGPT provides a command-line binary that accepts a single document or a folder of documents. To adapt this tool for large-scale processing within *py-semtools*, we created the llmEngine interface. This interface maintains the same design principles as fmEngine and stEngine, allowing large collections of compressed or grouped files to be processed in benchmark workflows. Users provide a text corpus, a grounding template and a reference to a language model (API call or local deployment). The corpus can be split into paragraphs, sentences or left as full documents. Each text unit is passed to the SPIRES engine via the extract_from_text method, and the resulting ontology terms are collected in an output file containing all query-corpus relationships, analogous to the outputs of fmEngine and stEngine (though without a numerical similarity score).

For reproducibility, the open-source “llama3.2:3b” model^68^ through the model manager ollama (https://ollama.com) was used as the underlying language model for OntoGPT queries, allowing local deployment for all experiments.

#### Ontological similarity

To compare the phenotypic profiles composed by Human Phenotype Ontology (HPO) terms in this work, we chose a similarity method based on Emission-Reception Information Content (ERIC), developed by Li and collaborators.^69^ According to their study, ERIC is particularly tolerant of noise and imprecision in clinical phenotypes. The formula (Equation 1) used in their work to calculate the similarity between two terms of an ontology, *t*_1_ and *t*_2_, is as follows:

(Equation 1) $$ERIC\left(t_{1},t_{2}\right)=\max[0,2\times IC\left(t_{MICA}\right)-\min\left(IC\left(t_{1}\right),IC\left(t_{2}\right)\right))$$

Where IC represents the information content (IC) of a phenotype term, and *t*_*MICA*_ refers to the most informative common ancestor of *t*_1_ and *t*_2_. For this study, we developed a modified version of this similarity implemented in *py-semtools*, referred to as Weight Normalized Emission-Reception Information Content (WNERIC), to obtain normalized values for subsequent processing. WNERIC implementation was performed according to the following formula (Equation 2):

(Equation 2) $$WNERIC\left(t_{1},t_{2}\right)=\frac{ERIC\left(t_{1},t_{2}\right)}{\max\left(IC\left(t_{ont}\right)\right)}$$

Where *t*_*ont*_ represents all the terms in the ontology, and the normalization is performed using the maximum IC obtained from any term within the ontology.

#### Ontological similarity clustering

To identify subgroups of related phenotypic profiles *py-semtools* has a dynamic clustering algorithm. This algorithm takes all the WNERIC pairwise similarities to build ontological similarity matrix *S*. This S matrix is converted to a distance *D* matrix performing *D*=1-*S*. Then, a hierarchical clustering is performed using Ward variance minimization method to obtain the linkage matrix. For each branching event (*b*) in the linkage matrix a partition density index (*PDI*) is calculated as described in Equation 3.

(Equation 3) $$PDI_{b}=\left(1+med\left\{{\bar{d}}_{1},{\bar{d}}_{2},...{\bar{d}}_{M}\right\}\right)\times \left(1-\frac{\sum_{i=1}^{M}L\left(C_{i}\right)}{N+1}\right)$$

A branching event (*b*) generates M clusters and for each resulting cluster (*C*_*i*_). The average distance (${\bar{d}}_{i}$) is calculated from the matrix *D* and the median (med) is obtained across all these ${\bar{d}}_{i}$. This first factor of the PDI, captures the internal coherence of the cluster. The other factor to take into account is the number of profiles (*L*(*C*_*i*_)) across the *M* clusters compared with N, total amount of profiles analysed. In this sense, PDI values close to 0 represents small and very coherent clusters while near to 2 means that the clusters are large and with very low similarities between profiles. Once the PDI is obtained for each branching event *b* in the obtained clustering tree, the tree cut is established at the lowest PDI value.

#### Ontological representations

The *py-semtools* package has visualizations that can represent term distributions across ontology or compare ontology profiles. One such visualization is the level distribution plot, which illustrates the distribution of terms across different hierarchical levels within a set of described profiles. By comparing these distributions to the expected frequencies based on the ontology structure, this plot reveals whether the annotations tend to be more general or highly specific. Another key plot is the ontology plot, or *ontoplot*, which employs a concentric ring distribution to display the terms from the root of the ontology outward to its branches. The terms are arranged based on the shortest path from the root, with each term level reflecting its proximity to the root. The plot uses color coding to denote the primary branch to which each term belongs, while the size of each dot represents the frequency of the term’s occurrence within the provided profile collection. This method enables a more nuanced analysis of phenotype specificity and frequency, categorized by major ontology branches, to enhance the characterization of the patient cohort.

Another useful visualization is the profile comparison heatmap, which compares one reference profile against a set of query profiles (i.e., a patient HPO profile against disease HPO profiles). It represents the ontological similarity between each term in the reference profile and its most similar phenotype in the query profiles. The X-axis arranges the query profiles according to their similarity to the reference profile, while the Y-axis ranks the terms in the reference profile based on their relevance, determined by their ontological similarity to the corresponding terms in the query profiles.

#### Biomedical text corpora to identify diseases and phenotypes

The text corpus chosen for this work was composed of all abstracts available in PubMed and all full-text articles available in PubMed Central (a repository with open-access articles). All PubMed abstracts were downloaded via the National Center for Biotechnology Information (NCBI) FTP service (https://ftp.ncbi.nlm.nih.gov/) on April 29, 2024, by retrieving all files from the “pubmed/baseline” directory. Similarly, full-text articles from PubMed Central were obtained on May 21, 2024, using the same FTP service. The files were retrieved from the “/pub/pmc/oa_bulk” directory, including documents from the “oa_comm”, “oa_noncomm”, and “oa_other” folders, encompassing both baseline and incremental files. A predefined set of terms was used as a blacklist to exclude conference abstracts during the analysis of full-text articles, resulting in the removal of 14,722 elements.

#### Benchmarking datasets

One of the modules developed in *py-semtools*, stEngine, was designed for embedding documents and calculating textual semantic similarity. To assess its performance, we selected well-established gold-standard resources. They needed to meet specific criteria, including the association of a set of diseases, either manually or automatically (with high accuracy), to at least one scientific publication per disease, in which a comprehensive investigation or review of the disease had been conducted.

One such gold-standard dataset was obtained from Ehrhart et al.^63^, which described a manual search and curation process to compile a dataset of 4 166 rare monogenic diseases linked to 3 163 causative genes, along with the PubMed identifiers (PMIDs) of the scientific publications that first described these diseases. The data from this study was downloaded, and the fields corresponding to the Online Mendelian Inheritance in Man (OMIM) identifier of each disease and the PMID of the related document were extracted. This gold-standard dataset will be referred to as “OMIM-Ehrhart”.

Another resource was derived from the Disease Ontology (DO).^70^ This ontology, available on the Open Biological and Biomedical Ontologies (OBO) Foundry website (https://obofoundry.org/ontology/doid.html), was processed using *py-semtools*. We searched for those disease terms in the ontology that had cross-references, represented by the “xref” field, with OMIM identifiers. For the terms selected from the previous step, we searched in the “def” field for additional information about links to documents with an identifiable PMID, searching the pattern “url:https//www.ncbi.nlm.nih.gov/pubmed/[PMID]”. This data was programmatically obtained so that a table with OMIM diseases and the PMIDs of the corresponding documents was obtained. This gold-standard dataset is referred to as “OMIM-DO”.

To assess the capability of *py-semtools* engines for phenotypic annotation of documents, we retrieved scientific publications referenced in both gold-standard datasets. Since each piece of evidence was associated with a PMID, we constructed the text corpus from two sources: the collection of all abstracts available in the PubMed repository and the collection of full-text articles hosted in PubMed Central.

Additionally, for each of the diseases associated with a PMID for any of the gold-standard datasets (“OMIM-Ehrhart” and “OMIM-DO”), the disease-associated HPO terms were obtained from the OBO Library (http://purl.obolibrary.org/obo/hp/hpoa/phenotype.hpoa). These HPO terms were used to annotate the OMIM diseases in each gold-standard dataset, providing an enriched layer of phenotypic information. This annotation will be referred to as “disease-profiles”.

Furthermore, we obtained a dataset focused exclusively on real patients from the Monarch Phenopacket Store, an open-source repository and data collection maintained by the Monarch Initiative that provides cohorts of GA4GH Phenopackets describing individuals with Mendelian diseases.^71^ The latest version of the resource includes 6,668 phenopackets representing 475 Mendelian and chromosomal diseases, associated with 423 genes and 3,834 unique pathogenic alleles curated from 959 publications.

From this resource, we downloaded information on patient identifiers, OMIM disease identifiers, phenotypic profiles and the PMID of the publication from which the phenotypic data were derived. Records with insufficient phenotypic descriptions were filtered out by removing those containing fewer than seven phenotypes. To reduce the potential overrepresentation of certain diseases due to multiple cases reported in the same publication, the number of patients was limited to ten per article, retaining those with the most detailed phenotypic profiles. After applying these filtering criteria, the final dataset comprised 1,420 individuals corresponding to 226 unique diseases and 450 PMIDs.

In this dataset, we focus on identifying the document originally associated with each case using the corresponding phenotypic profile, as the articles contain detailed descriptions of the clinical manifestations of the studied disease. This design allows different phenotypic profiles to be evaluated independently to determine whether they can retrieve the correct disease-related publication. This gold-standard dataset is referred to as “MPS”.

We additionally increased the challenge of distinguishing between relevant and irrelevant information to evaluate the precision of our methodology. For this, we expanded the gold-standard datasets by adding unrelated documents to the text corpus. For each gold-standard record, a total of three random documents were added for each existing document within it.

#### Benchmarking using different disease prediction approaches

In this benchmark, queries were categorized into two distinct groups depending on the biomedical concept used (diseases or phenotypes): for Direct Disease Prediction (DDP, Figure 1B) and for Indirect Phenotype-based Disease Prediction (IPDP, Figure 1C). DDP involves identifying explicit mentions of diseases in the corpus, while IPDP prediction leverages phenotypic terms to infer relevant diseases. The assessment was performed in both types of text corpora (abstracts and full-text articles), for all documents available in the gold-standard datasets (“OMIM-Ehrhart”, “OMIM-DO” and “MPS”).

#### Direct Disease Prediction (DDP)

Using this approach, we aimed to confirm the ability of our methodology to associate each OMIM identifier from the gold-standard datasets with its corresponding PMID by identifying the disease name within the text corpus. This step was intended to establish a baseline for comparison with the IPDP method.

In this prediction approach, implemented through the DDP workflow (Figure 1B), the file “mimTitles.txt” with the disease identifiers and their names was obtained from the OMIM download section (https://omim.org).^72^ From this file, we selected all records with OMIM codes classified with a number (#) or a percentage (%) symbols, forming the set of diseases used as queries. This set will henceforth be referred to as the “OMIM-list”.

For the corpus set, both all abstracts available in PubMed and all full-text articles available in PubMed Central were filtered according to each gold-standard (“OMIM-Ehrhart”, “OMIM-DO” and “MPS”,) and processed with the get_corpus_index module of *py-semtools*. This module indexes large text corpora, extracting and preparing their content for use with both fmEngine and stEgine, ensuring efficient retrieval and text analysis.

Next, both the “OMIM-list” and the extracted text fragments from the documents of the corpus were processed using fmEngine and stEngine. Once the comparisons between both sets were applied, the diseases included in the “OMIM-list” were searched in the corpus to identify the document that exhibited the highest score to that disease. Since each document was divided into multiple sentences, the overall score of a document was defined as the highest score among its individual sentences. We referred to this metric as “textual semantic similarity” in the case of the stEngine module.

#### Indirect Phenotype-based Disease Prediction (IPDP)

We evaluated the IPDP approach (Figure 1C) to confirm the ability of the engines to accurately associate each gold-standard disease with its corresponding document. This association was based on the similarity in HPO terms between the phenotypic profile of the disease and the phenotypes described in the document. The document whose phenotypic profile has the highest ontological similarity to the disease profile is likely to be the one that is describing the disease.

In the IPDP workflow (Figure 1C), the texts used as queries were the phenotype names and their corresponding HPO identifiers obtained from the HPO.^26^ To achieve this, the ontology was processed using *py-semtools* and the HP.obo file available on the HPO website (https://hpo.jax.org/data/ontology). All terms that are descendants of the phenotype “Phenotypic abnormality” (HP:0000118) were extracted. This set of terms will henceforth be referred to as the “HPO-list”.

In this workflow, we identified for each disease the documents in the corpus with the highest similarity by performing a phenotypic profile comparison. To achieve this, we first generated what we refer to as the “phenotypic profiles of documents”, a process based on phenotypic annotation.

The corpus used in this approach consists of the subset of abstracts and articles included in the gold-standards (“OMIM-Ehrhart”, “OMIM-DO” and “MPS”). We began by using the get_corpus_index module to create corpus indexes from this raw text corpora. Next, we process the phenotypes from the “HPO-list” and text fragments from the corpus indexes using the fmEngine and stEngine modules. Once both the set of phenotypes and the set of document fragments were prcessed, we identified text fragments that might mention phenotypic descriptions and map them to HPO-defined phenotypes.

To accomplish this, we performed all possible comparisons between the sets of phenotypes and corpus document fragments, and only kept those pairs that had a score equal to or greater than the optimum determined for each algorithm (see results). Using the document identifier, the phenotypes finally related to any of the sentences in that document were aggregated to form the phenotypic profile of that document.

Finally, we conducted an ontological comparison of phenotypic profiles. For each disease in the gold-standard datasets, we compared its phenotypic profile against all the phenotypic profiles present within the documents, using the WNERIC similarity method defined before, implemented in *py-semtools*. We referred to this metric as “ontological semantic similarity”.

#### Measuring performance in benchmarks

The results obtained in the DDP and IPDP workflows were evaluated based on the similarity values obtained for the documents contained in each gold-standard. In the DDP workflow evaluation, for each disease, the resulting list of documents and their scores were sorted in descending order. In the IPDP workflow evaluation, for each disease, the documents were sorted from highest to lowest ontological semantic similarity values.

The evaluation of DDP and IPDP workflows was performed in a similar manner. For each disease or patient, we assigned ranking positions for every document based on the score sorting. Only the document-disease relationships found in both gold-standard datasets were considered. The evaluation metric we defined was, for a given disease or patient, as the ranking position of the correct document-disease or document-patient pair.

To make all conditions comparable, in each condition the ranking positions of the documents was scaled by the total number of documents. This was defined as the number of gold-standard documents plus the random documents added. The total number differs according to the workflow (DDP or IPDP), the gold-standard (“OMIM-DO”, “OMIM-Ehrhart” or “MPS”) and the type of document (abstract or article). The resulting scaled ranking values range between 0 and 1, with values closer to 0 meaning better ranks.

#### Case study: Workflow application with RASopathies

To demonstrate the application of our methodology to suggest relevant literature using a phenotype-based approach in a real-life scenario, we analyzed different RASopathies as a case of study, including Neurofibromatosis type-1 (NF1),^29^ Neurofibromatosis-Noonan (NFNS),^30^ Noonan,^31^ Costello^32^ and Cardiofaciocutaneous (CFC) syndromes.^33^ As described in,^73^ the RASopathies are “a clinically defined group of medical genetic syndromes caused by germline mutations in genes encoding components or regulators of the Ras/mitogen-activated protein kinase (MAPK) pathway”. They are characterized by dysregulations of the Ras/MAPK pathway, which results in a shared set of overlapping phenotypic features.^74^ However, each syndrome also presents unique phenotypic characteristics that distinguish it from the others,^33^^,^^75^^,^^76^ highlighting both the commonalities and the diversity within this group of disorders.

For these diseases, we searched for their OMIM identifiers (162200, 601321, 163950, 218040 and 115150, respectively) and obtained their phenotypic profile using the same annotation strategy used for both the gold-standard datasets described in this study.

The annotation of documents was carried out following the workflow depicted in Figure 1A, with only the stEngine module using the whole set of abstracts and full-text articles available in PubMed.

Then, using *py-semtools*, we performed a comparison of the phenotypic profiles of each of the RASopathies against the phenotypic profiles of all annotated PubMed documents. For each disease, we selected the 50 documents with the highest ontological semantic similarity index to analyze them and confirm whether their content is related to the selected RASopathies.

#### Computational resources

The analysis in this study were carried out on the Picasso computing cluster at the Supercomputing and Bioinnovation Centre (SCBI) of the University of Malaga (see acknowledgments), a resource shared by the institution’s researchers. It has several node types for CPU computing and four DGX-A100 nodes with eight GPUs (Nvidia A100) and 1 TB of RAM for GPU computing. For tools capable of taking advantage of GPU acceleration, such as the bi-encoder and cross-encoder models from Sentence Transformers and OntoGPT, similarity computations were performed on one of the DGX-A100 nodes, assigning each model to an available GPU. For similarity calculations with models that do not support GPU acceleration but allow CPU multithreading, such as TF-IDF and BM25, jobs were executed specifically on SD530 nodes with 52 cores (Intel Xeon Gold 6230R @ 2.10GHz) and 192 GB of RAM.

### Quantification and statistical analysis

All analyses were performed using Python version 3.12.0, the Python libraries *scipy* v1.13.1, *sentence transformers* v3.2.0, and the Python library developed for this work, *py-semtools*. For the figures available in this work, the libraries *seaborn* v0.13.2, *plotly* v6.1.2, and the JavaScript library *CanvasXpress* v57.2 were used.

All other statistical results are presented in the results section, with detailed metrics provided in figure legends and supplementary tables.

## Supplemental information

Document S1. Figures S1–S11 and Tables S1–S20

Data S1. Clustering parameters An Excel file containing the parameter combination and metric performance result for the web portal documents search implementation, used for finding the optimal clustering parameters.
