# Supplementary material for: A text mining and ontology-based approach using phenotypes to obtain relevant literature for rare diseases
Source: iScience. 2026 Aug 13;29(9):117183. doi: 10.1016/j.isci.2026.117183 (PMC13495350; doi:10.1016/j.isci.2026.117183)
Supplement: Supplementary file 1 [file mmc1.pdf]

iScience, Volume *29*

Supplemental information

A text mining and ontology-based approach using phenotypes to obtain relevant literature for rare diseases

Jesús Pérez-García, Federico García-Criado, Florencio Pazos, Mónica Chagoyen, Elena Rojano, Pedro Seoane, and Juan A.G. Ranea

Supplementary figures

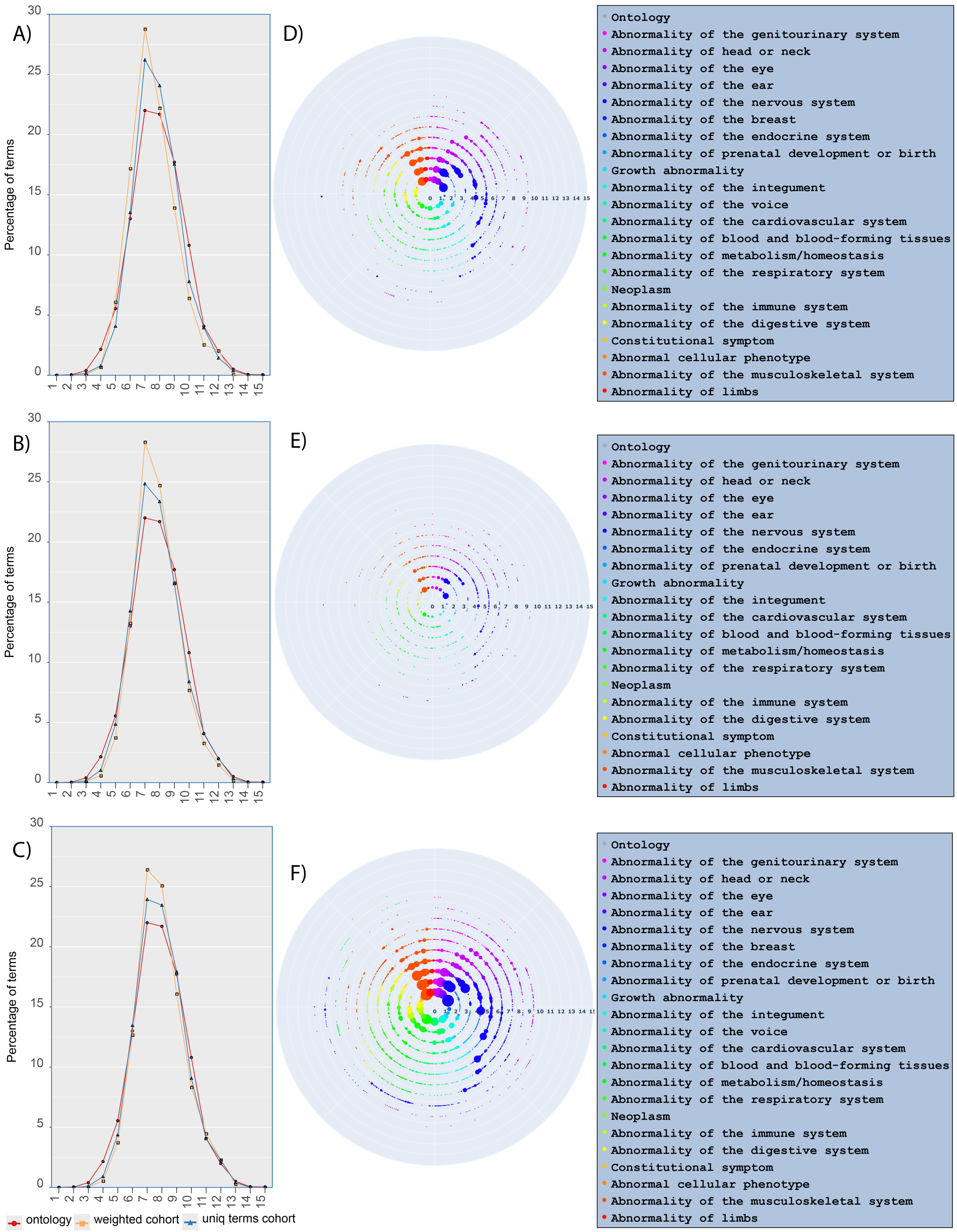

Figure S1: Depth level of phenotypes (shortest path from the root) for diseases and annotated documents associated with “OMIM-DO” gold-standard dataset. (A) Dataset diseases. (B) Annotated abstracts with PubMed identifiers (PMIDs). (C) Annotated full-text articles with PubMed Central identifiers (PMCIDs). Ontological signatures of diseases and annotated documents associated with “OMIM-DO” gold-standard dataset. (D) Ontological signature of dataset diseases. (E) Ontological signature of annotated abstracts with PMIDs. (F) Ontological signature of annotated full-text articles with PMCIDs. The center of the circle represents the phenotype “Phenotypic abnormality”, with code HP:0000118, which is taken as the root term. Each concentric circle represents one level of the ontology, from the center outward. Each phenotype in represented at the level equivalent to its depth, defined as the shortest path from the root term of the ontology. The colors represent the child phenotypes of the root term, used to categorize at a general level to which branch more specific phenotypes belong. The dot size of each phenotype refers to the frequency with which it is found within the analyzed profiles.quency with which it is found within the analyzed profiles.

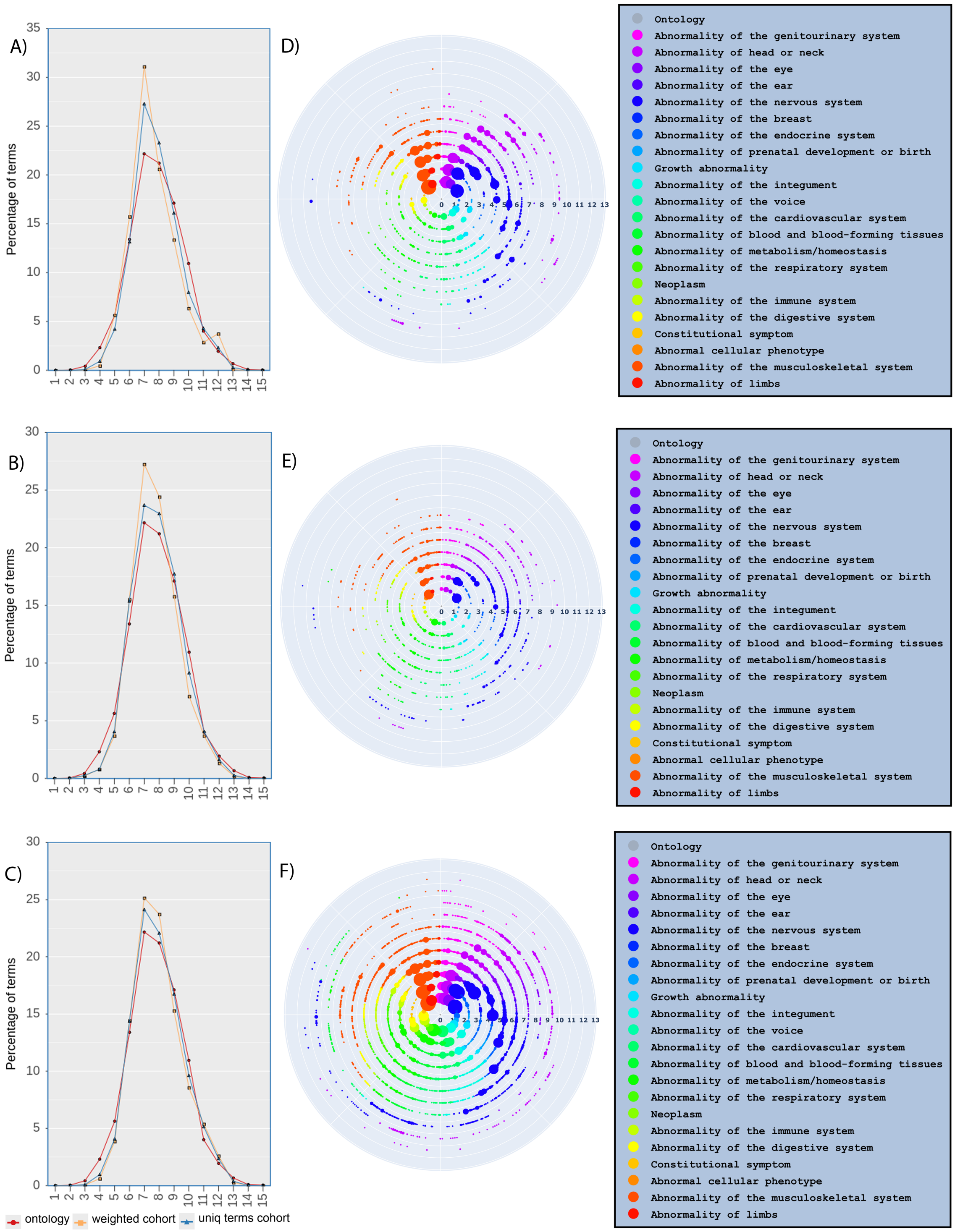

Figure S2: Depth level of phenotypes (shortest path from the root) for patients and annotated documents associated with "MPS" gold-standard dataset. (A) Patient profiles. (B) Annotated abstracts with PubMed identifiers (PMIDs). (C) Annotated full-text articles with PubMed Central identifiers (PMCIDs). Ontological signatures of patients and annotated documents associated with "MPS" gold-standard dataset. (D) Ontological signature of patients profiles. (E) Ontological signature of annotated abstracts with PMIDs. (F) Ontological signature of annotated full-text articles with PMCIDs. The center of the circle represents the phenotype "Phenotypic abnormality", with code HP:0000118, which is taken as the root term. Each concentric circle represents one level of the ontology, from the center outward. Each phenotype in represented at the level equivalent to its depth, defined as the shortest path from the root term of the ontology. The colors represent the child phenotypes of the root term, used to categorize at a general level to which branch more specific phenotypes belong. The dot size of each phenotype refers to the frequency with which it is found within the analyzed profiles.quency with which it is found within the analyzed profiles.

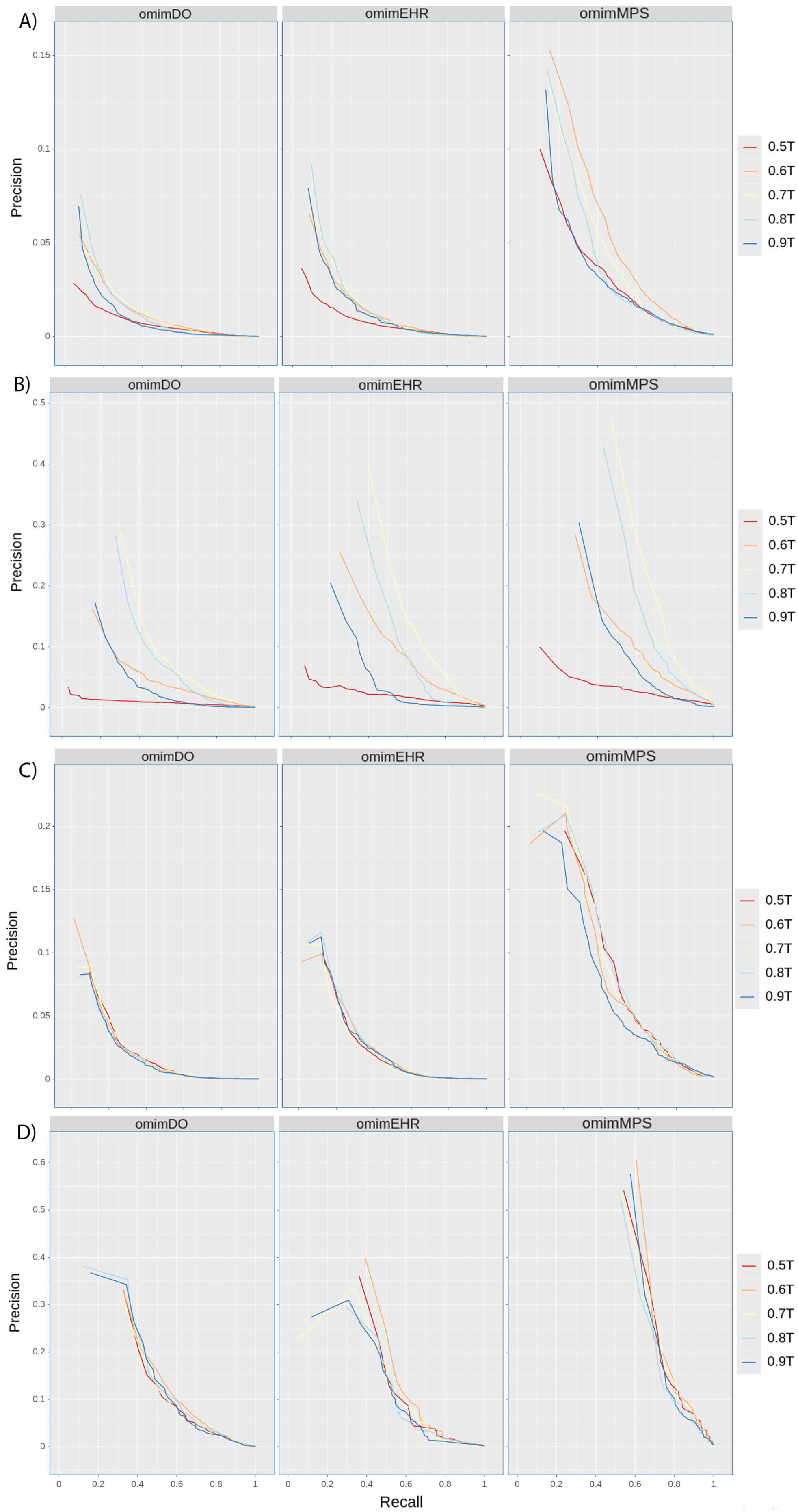

Figure S3: Precision/Recall curves obtained for the stEngine module methods: Sentence Transformer (A, B) and re-rank Crossencoder (C,D), using different similarity thresholds for the "OMIM-DO", "OMIM-Ehrhart" and MPS datasets. A) Sentence Transformer using only abstracts. B) Sentence Transformer using only full articles. C) Re-rank Crossencoder using only abstracts. D) Re-rank Crossencoder using only full articles.

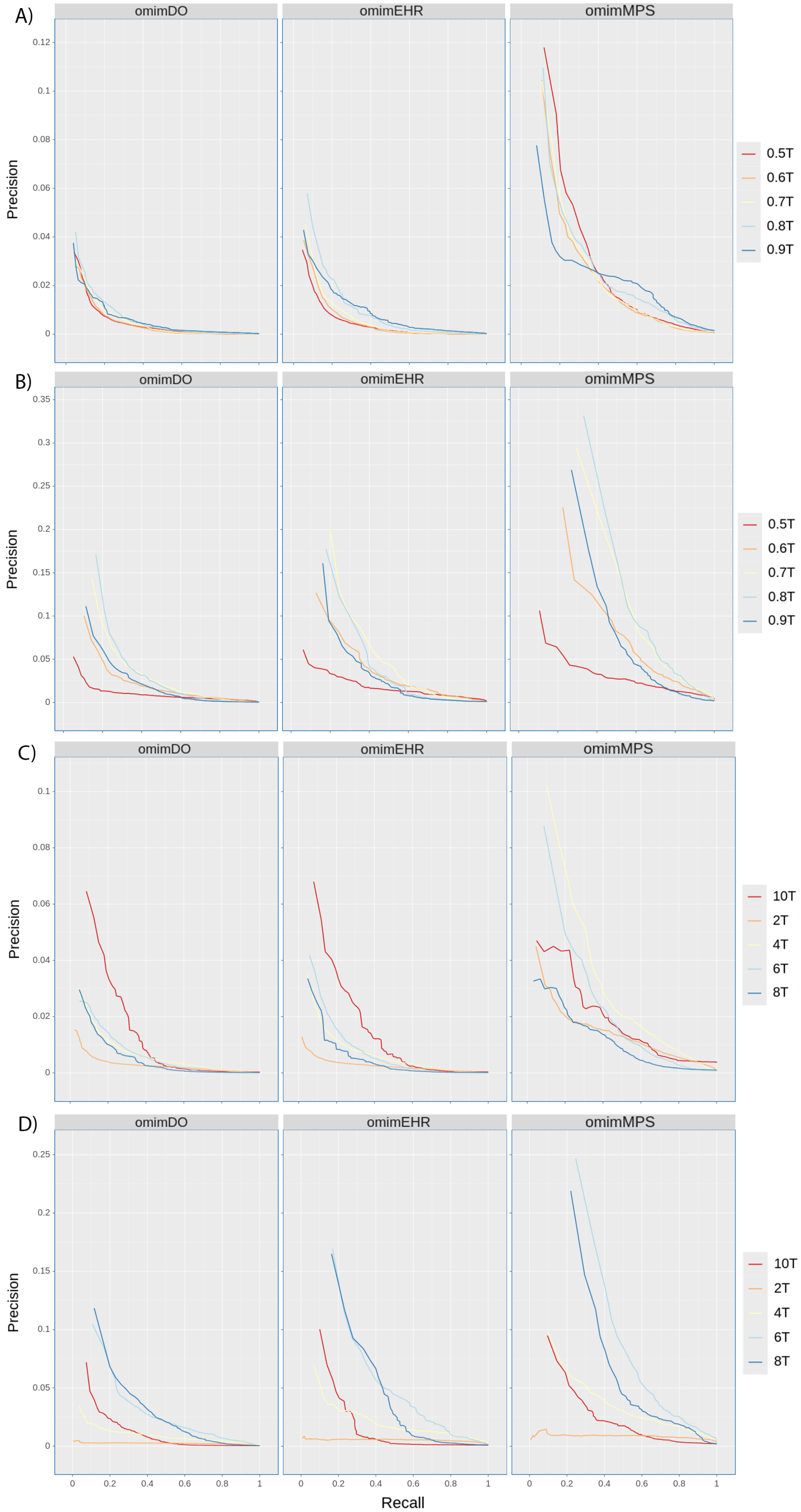

Figure S4: Precision/Recall curves obtained for the fmEngine module methods: TF-IDF (A,B) and BM25 (C,D), using different score thresholds for the “OMIM-DO”, “OMIM-Ehrhart” and MPS datasets. A) TF-IDF Using only abstracts. B) TF-IDF using only full articles. C) BM25 Using only abstracts. D) BM25 using only full articles.

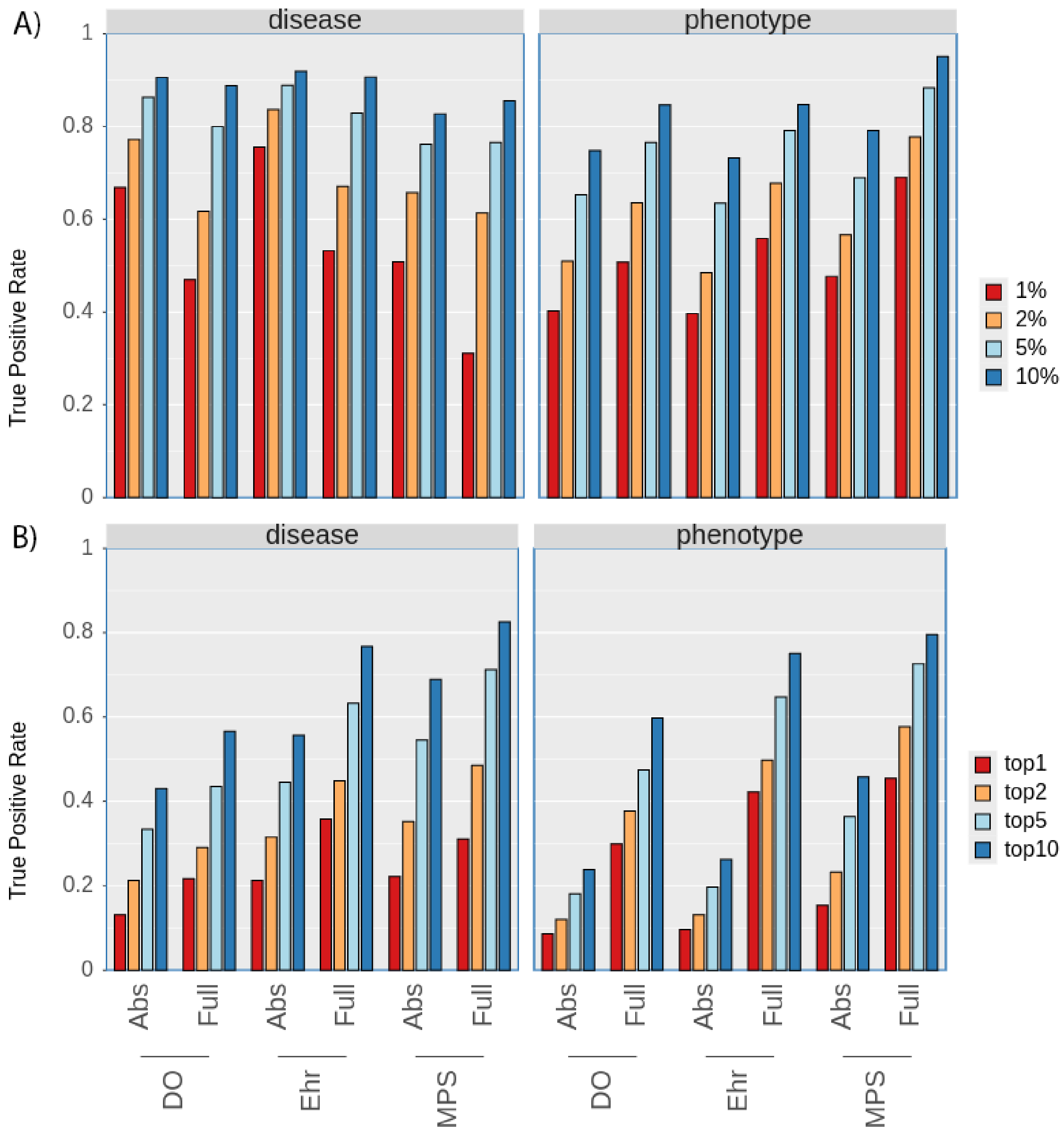

Figure S5: Sentence Transformers True Positive Rate (TPR) calculated using different cut-off thresholds to the ranking of similarity values. A) Percentage thresholds: 1%, 2%, 5% and 10% . B) Absolute thresholds: 1, 2, 5 and 10. "Disease" corresponds to the DDP workflow, while "Phenotype" refers to the IPDP workflow. Labels: Abs (abstracts), Full (full-text articles), DO ("OMIM-DO" dataset), Ehr ("OMIM-Ehrhart" dataset).

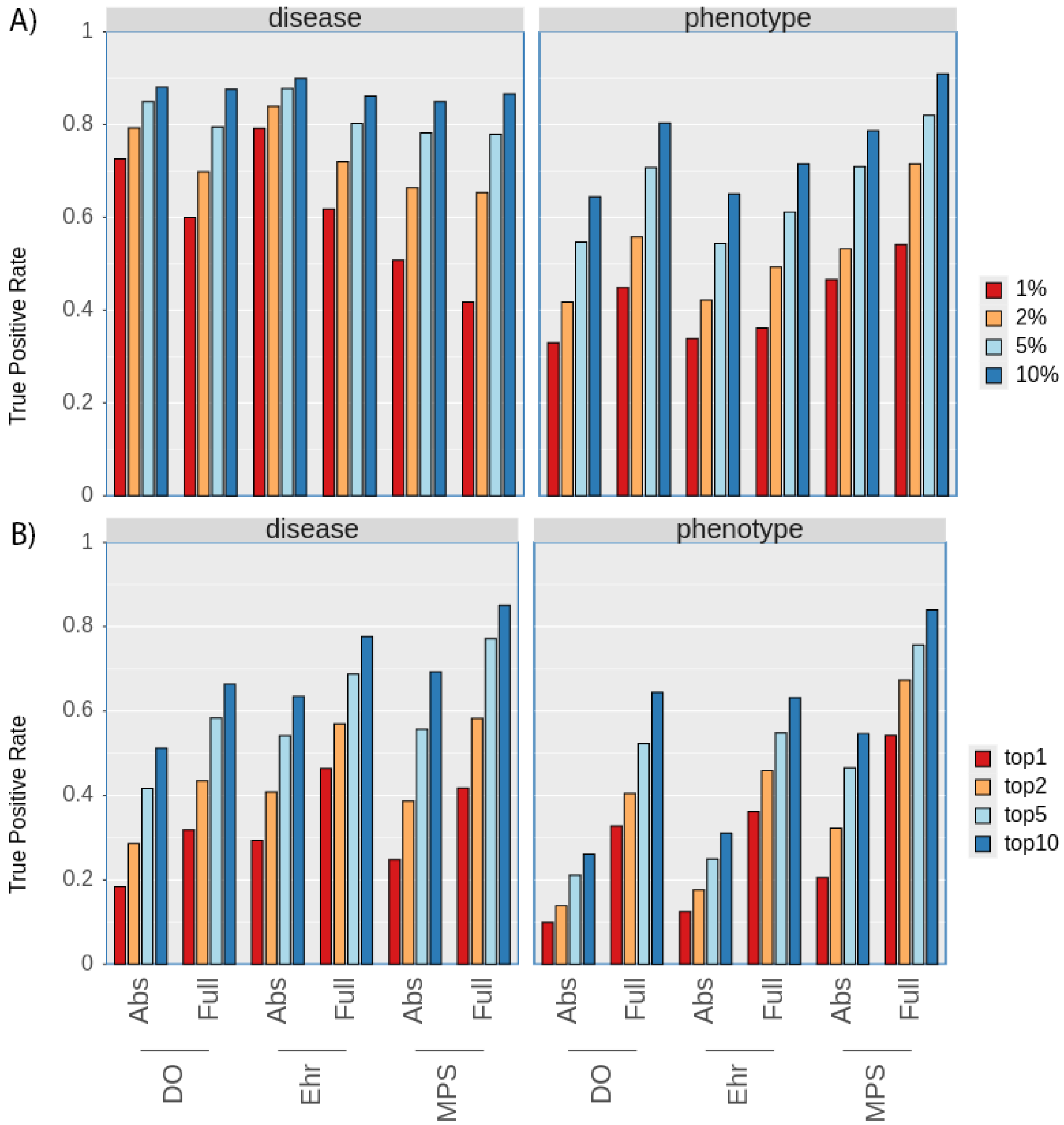

Figure S6: Re-rank Crossencoder True Positive Rate (TPR) calculated using different cut-off thresholds to the ranking of similarity values. A) Percentage thresholds: 1%, 2%, 5% and 10% . B) Absolute thresholds: 1, 2, 5 and 10. “Disease” corresponds to the DDP workflow, while “Phenotype” refers to the IPDP workflow. Labels: Abs (abstracts), Full (full-text articles), DO (“OMIM-DO” dataset), Ehr (“OMIM-Ehrhart” dataset).

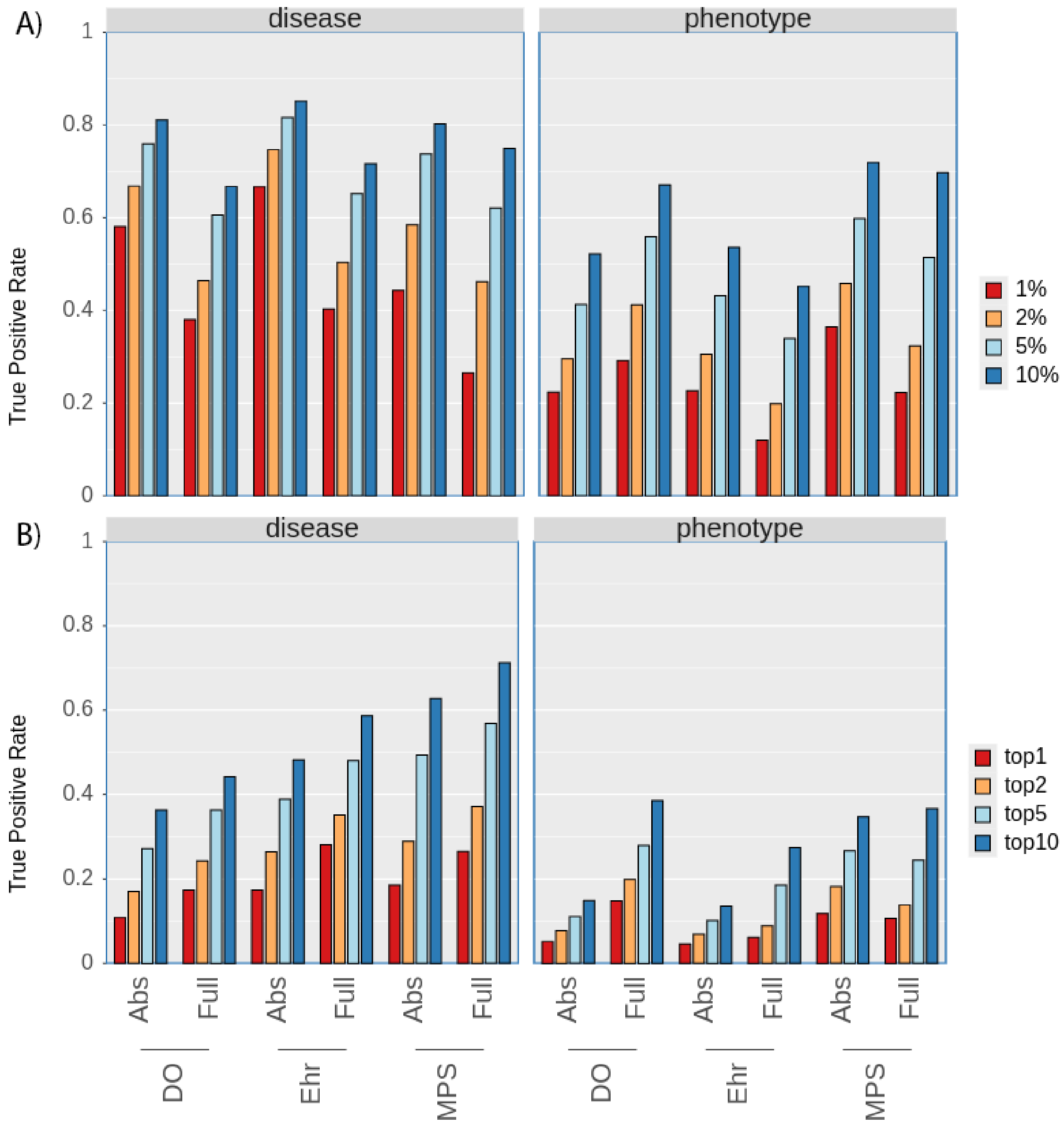

Figure S7: TF-IDF True Positive Rate (TPR) calculated using different cut-off thresholds to the ranking of similarity values. A) Percentage thresholds: 1%, 2%, 5% and 10% . B) Absolute thresholds: 1, 2, 5 and 10. "Disease" corresponds to the DDP workflow, while "Phenotype" refers to the IPDP workflow. Labels: Abs (abstracts), Full (full-text articles), DO ("OMIM-DO" dataset), Ehr ("OMIM-Ehrhart" dataset).

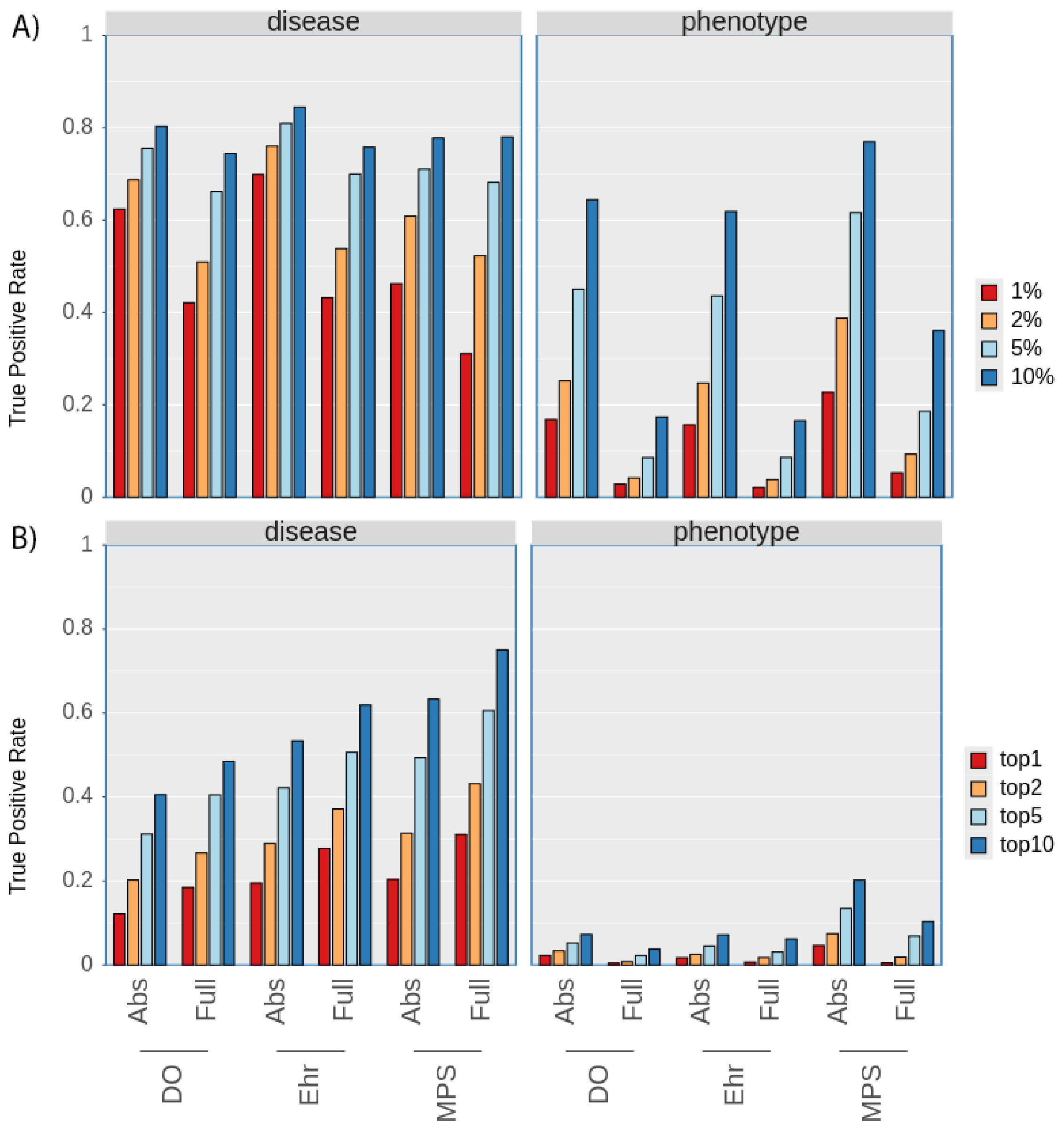

Figure S8: BM25 True Positive Rate (TPR) calculated using different cut-off thresholds to the ranking of similarity values. A) Percentage thresholds: 1%, 2%, 5% and 10% . B) Absolute thresholds: 1, 2, 5 and 10. “Disease” corresponds to the DDP workflow, while “Phenotype” refers to the IPDP workflow. Labels: Abs (abstracts), Full (full-text articles), DO (“OMIM-DO” dataset), Ehr (“OMIM-Ehrhart” dataset).

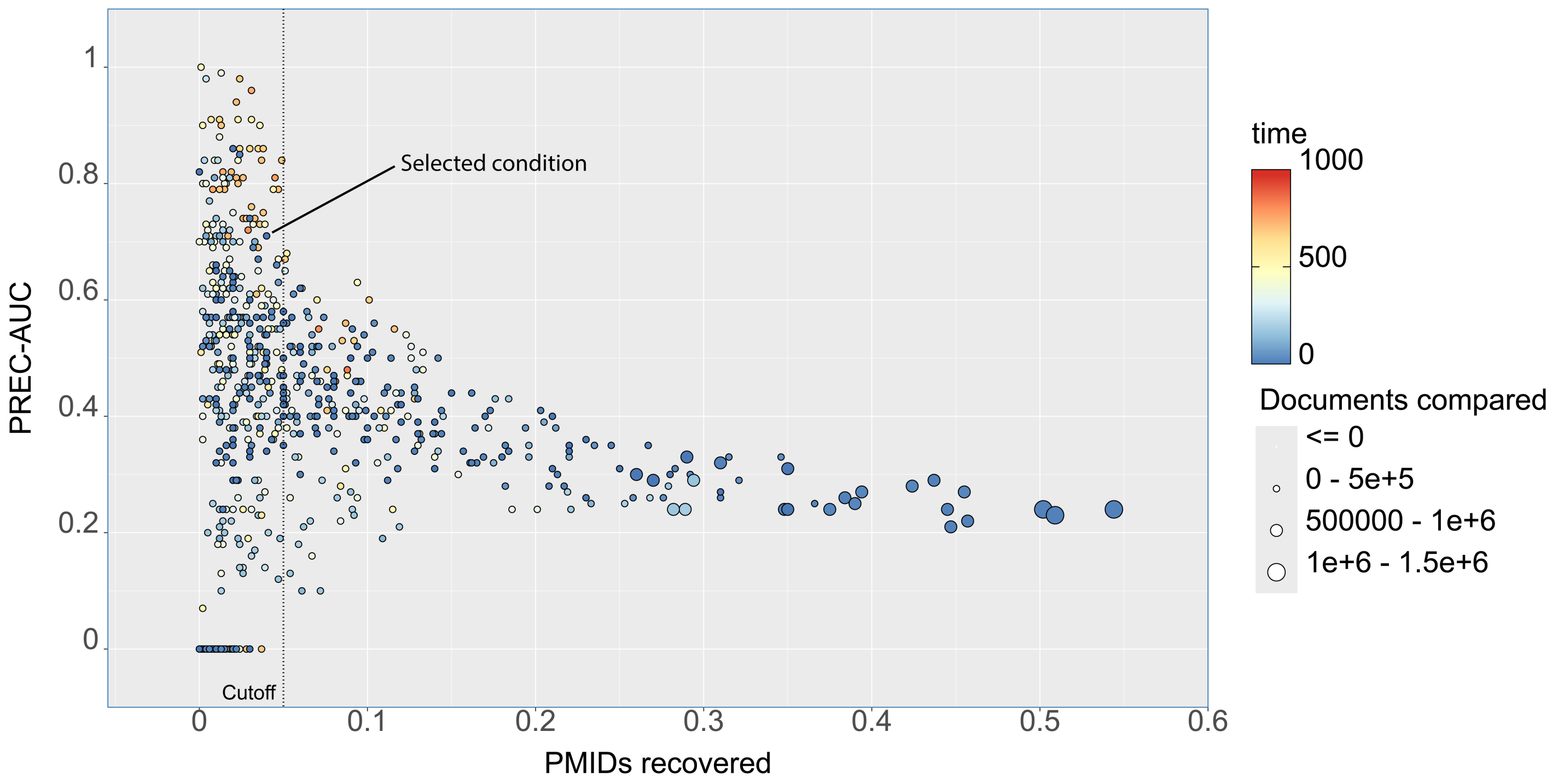

Figure S9: Parameter optimization for the web-based retrieval pipeline using the RASopathies case study. The x-axis denotes the proportion of gold-standard PMIDs successfully retrieved within the top-K clusters, while the y-axis represents the Area Under the Precision-Recall Curve (PR-AUC) for the identified documents (PMIDs). Each data point corresponds to a unique combination of five parameters: the HPO ontological level for phenotype simplification, the minimum document profile size, the total number of K-means clusters, the frequency threshold for cluster representative profiles, and the number of top-K clusters evaluated. Point color indicates the execution time in seconds, and point size reflects the total document volume processed within the selected top-K clusters for that configuration.

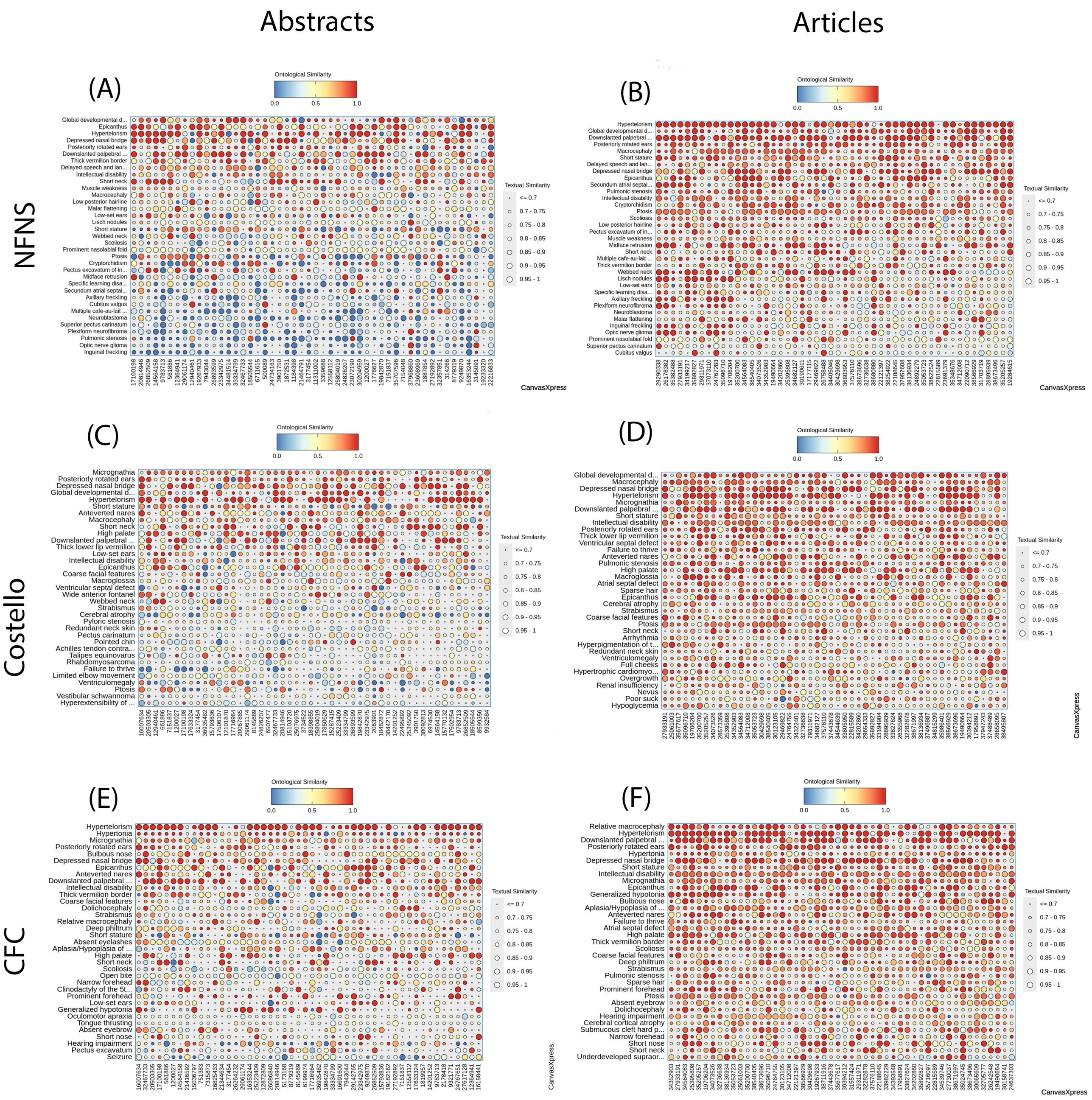

Figure S10: Ontological comparison between document-derived phenotypic profiles and those of selected RASopathies. Heatmaps show similarities between profiles from annotated PubMed abstracts and PubMed Central full-text articles and the reference (syndrome) profiles of Neurofibromatosis-Noonan (NFNS), Costello and Cardiofaciocutaneous(CFC) syndromes: (A) Abstracts vs. NFNS syndrome, (B) Articles vs. NFNS syndrome, (C) Abstracts vs. Costello syndrome, (D) Articles vs. Costello syndrome, (E) Abstracts vs. CFC syndrome, (F) Articles vs. CFC syndrome. The PubMed IDs of the first 50 documents most closely related to the disease profile are shown on the X axis, ordered from highest to lowest ontological semantic similarity. The Y axis shows the names of each of the phenotypes described in the disease, ordered from highest to lowest by the mean ontological semantic similarity of the given term against each of the matched terms in the documents. Each cell color represents the ontological semantic similarity value, limited between 0 and 1, obtained by that disease phenotype against the matched term in that document (not shown in this figure). The size of the point reflects the textual semantic similarity, measured by cosine similarity, between the document sentence vector and the vector of the HPO phenotype that was mapped in that sentence of the document (not shown in the figure).

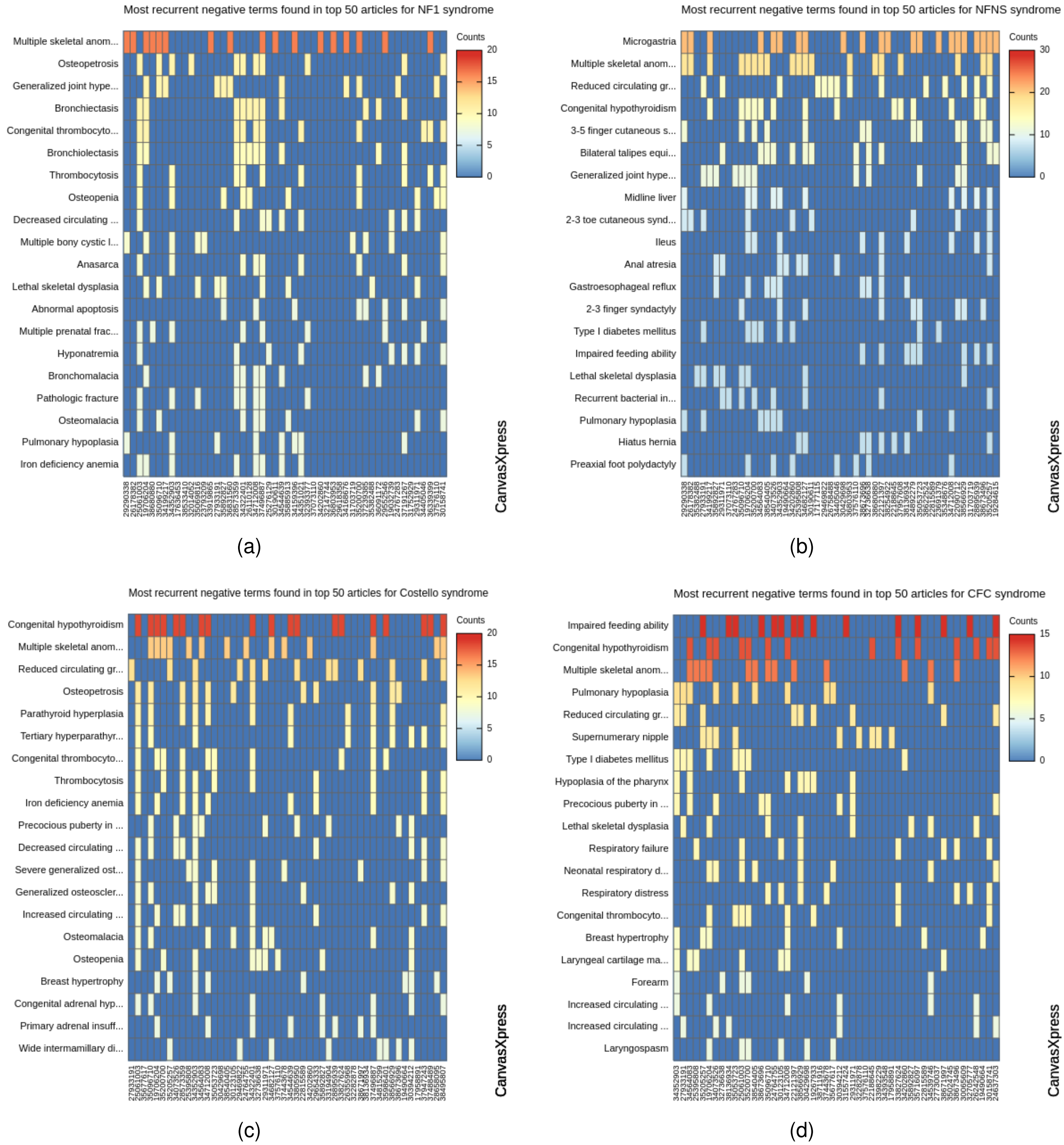

Figure S11: Heatmap representation of the most abundant negative terms (terms that are not closely related, in terms of ontological similarity, to the disease profile, but are prevalent in the annotated documents) of the top 50 full-text articles recommended for (A) Neurofibromatosis type-1 (NF1) (B) Neurofibromatosis-Noonan (NFNS) (C) Costello (D) Cardiofaciocutaneous (CFC) syndromes. On the X axis the PMIDs are shown in the same order as the top 50 recommended articles for the syndrome in question. The Y-axis shows the phenotypes annotated in the documents but not related to the disease profile in question, ordered according to the number of times they appear in the top 50 articles. For visualization purposes, only the top 20 terms are shown. The color represents in how many of the top 50 documents that given phenotype has been annotated.

Supplementary Tables

Table S1: Summary of key statistics on PubMed IDs (PMIDs) and diseases from the gold standards used to evaluate the tool in the Direct Disease Prediction (DDP) workflow. Each column represents a specific combination of gold standard and corpus. The selected gold standards were “OMIM-DO”, “OMIM-Ehrhart” and “MPS”, while the analyzed corpora included abstracts and full-text articles, further segmented into paragraphs and sentences. ”Abs” means abstracts and ”Art” means articles.

**Stat**	**OMIM-DO**			**OMIM-Ehrhart**			**MPS**		
	**Raw**	**Abs**	**Art**	**Raw**	**Abs**	**Art**	**Raw**	**Abs**	**Art**
**Diseases tested**	4046	4046	591	4005	4005	332	207	207	99
**Total PMIDs**	5055	5055	615	4235	4235	336	411	411	132
**Unique PMIDs**	4578	4578	542	3990	3990	299	398	398	129
**Unique PMIDs proportion**	0.9	0.9	0.88	0.94	0.94	0.88	0.96	0.96	0.97
**Average PMIDs per disease**	1.24	1.24	1.04	1.05	1.05	1.01	1.98	1.98	1.33
**PMIDs in only one disease**	4313	4313	497	3799	3799	274	386	386	127
**PMIDs in >1 disease**	265	265	45	191	191	25	12	12	2
**Mean repetitions of repeated PMIDs**	2.8	2.8	2.62	2.28	2.28	2.48	2.08	2.08	2.5

Table S2: Summary of key statistics on PubMed IDs (PMIDs) and diseases from the gold standards used to evaluate the tool in the Indirect Phenotype-based Disease Prediction (IPDP) workflow. Each column represents a specific combination of gold standard and corpus. The selected gold standards were “OMIM-DO”, “OMIM-Ehrhart” and “MPS”, while the analyzed corpora included abstracts and full-text articles, further segmented into paragraphs and sentences. ”Abs” means abstracts and ”Art” means articles.

**Stat**	**OMIM-DO**			**OMIM-Ehrhart**			**MPS**		
	**Raw**	**Abs**	**Art**	**Raw**	**Abs**	**Art**	**Raw**	**Abs**	**Art**
**Diseases/Patients tested**	4038	3288	575	4093	3179	328	1420	828	387
**Total PMIDs**	4904	4012	599	4350	3322	332	1383	828	387
**Unique PMIDs**	4467	3681	534	3963	3150	295	421	261	131
**Unique PMIDs proportion**	0.91	0.91	0.89	0.91	0.94	0.88	0.3	0.31	0.33
**Average PMIDs per entity**	1.21	1.22	1.04	1.06	1.04	1.01	0.97	1.0	1.0
**PMIDs in only one entity**	4218	3479	492	3657	3003	270	174	115	54
**PMIDs in >1 entity**	249	202	42	306	147	25	247	146	77
**Mean repetitions of repeated PMIDs**	2.76	2.64	2.55	2.26	2.17	2.48	4.89	4.88	4.32

Table S3: Top 25 most prevalent phenotypes for patients from DEE11 cohort extracted from Monarch Phenopacket Store.

**HP term**	**Patient percentage**
Epileptic encephalopathy	32.31
Intellectual disability, severe	24.68
Autism	23.40
Bilateral tonic-clonic seizure	21.88
Global developmental delay	21.37
Multifocal epileptiform discharges	18.82
Seizure	18.82
Generalized tonic seizure	18.32
Hypotonia	17.55
Infantile spasms	16.28
Focal-onset seizure	15.52
EEG with burst suppression	14.75
Hypsarrhythmia	11.95
Focal tonic seizure	11.95
EEG with abnormally slow frequencies	9.92
Microcephaly	8.9
Generalized myoclonic seizure	7.63
Intellectual disability	6.8
Intellectual disability, moderate	6.87
Abnormal repetitive mannerisms	6.87
Ataxia	6.1
Developmental regression	5.85
Delayed speech and language development	5.34
Brain atrophy	5.08

Table S4: Top 10 most similar articles to Neurofibromatosis type-1 (NF1), Neurofibromatosis-Noonan (NFNS), Noonan, Costello and Cardiofaciocutaneous (CFC) syndromes. The table presents the top 10 most similar full-text articles for each of the four RASopathies, ranked by their mean position across all syndromes. For each article, the ranking index (I), PubMed identifier (PMID), mean rank (Rank), title, and its ranking position within the top 50 for each syndrome (NF1, NFNS, Noonan, Costello and CFC) are shown. If an article was not within the top 50 for a given syndrome, a "-" symbol is used, and a value of 50 was assigned for mean rank calculations. In the title field, bold text highlights key terms such as "RASopathy", "rare disease", the name of one of the five syndromes, or the *NF1* gene. In the ranking columns, bold values indicate that name of the syndrome is mentioned within the body text of the article.

I	PMID	Rank	Title	NF1	NFNS	Noonan	Costello	CFC
1	27933191	4.6	genetics of inherited cardiocutaneous syndromes: a review	15	4	**1**	**1**	**2**
2	34352903	8.4	**rare disease**s of the orbit	**8**	16	**6**	11	1
3	19706204	10.2	encyclopaedia of tumour-associated familial disorders. part i: from aimah to chime syndrome	**4**	11	**25**	**5**	**6**
4	35096710	13.2	diagnostic approach to macrocephaly in children	**6**	10	**31**	**4**	**15**
5	35200700	13.8	cardiomyopathies in children and systemic disorders when is it useful to look beyond the heart?	37	12	**2**	**6**	12
6	25061003	15.4	question sets and answers	**3**	-	11	2	11
7	38540405	19.4	trisomy 22 mosaicism from prenatal to postnatal findings: a case series and systematic review of the literature	-	14	4	16	13
8	34073526	19.6	pallister–killian syndrome versus trisomy 12p—a clinical study of 5 new cases and a literature review	-	15	18	8	7
9	35892827	19.8	genetic disorders with predisposition to paediatric haematopoietic malignancies—a review	**16**	6	**9**	31	37
10	25395808	19.8	chromosomal and multifactorial genetic disorders with oral manifestations	-	19	16	10	4
11	32736638	23.2	multisystemic manifestations in a cohort of 75 classical ehlers-danlos syndrome patients: natural history and nosological perspectives	-	30	7	21	8
12	35205257	23.8	expanding the clinical phenotype of 19q interstitial deletions: a new case with 19q13.32-q13.33 deletion and short review of the literature	-	49	8	7	5
13	34564083	25.6	the role of rna-binding proteins in vertebrate neural crest and craniofacial development	-	13	-	12	3
14	29311971	27.0	update on 13 syndromes affecting craniofacial and dental structures	**46**	7	32	22	28
15	29290338	30.4	genotype-phenotype correlation in **nf1**: evidence for a more severe phenotype associated with missense mutations affecting **nf1** codons 844–848	**1**	1	**50**	-	-
16	26178382	30.81	high incidence of **noonan** syndrome features including short stature and pulmonic stenosis in patients carrying **nf1** missense mutations affecting p.arg1809: genotype–phenotype correlation	**2**	**2**	**-**	-	-
17	38136934	31.4	the **cardiofaciocutaneous** syndrome: from genetics to prognostic–therapeutic implications	**-**	36	**24**	**38**	**9**
18	35053723	32.4	microcephaly in neurometabolic diseases	-	38	-	14	10
19	34199217	32.41	severe phenotype in patients with large deletions of **nf1**	**7**	5	**-**	**-**	-
20	28573359	34.8	proceedings of the rami intern section meeting	18	-	**47**	**9**	-
21	35677617	35.61	multidisciplinary management of **costello** syndrome: current perspectives	**-**	-	**-**	**3**	**25**
22	38680880	37.21	**neurofibromatosis** type 1: optimizing management with a multidisciplinary approach	**5**	31	**-**	-	-
23	37073110	37.61	mutational spectrum and genotype-phenotype correlations in **neurofibromatosis** type 1 patients from north macedonia: identification of ten novel **nf1** pathogenic variants	**30**	8	**-**	-	-
24	35382488	38.41	giant cell lesions of the jaws involving **rasopathy** syndromes	**39**	3	**-**	**-**	**-**
25	24767283	40.41	**leopard** syndrome: clinical dilemmas in differential diagnosis of **rasopathies**	**43**	**9**	**-**	**-**	**-**
26	17222357	40.61	**noonan** syndrome	**-**	-	**3**	**-**	**-**
27	24083159	41.01	the other side of turner's: **noonan**'s syndrome	**-**	-	**5**	**-**	**-**
28	17636453	41.81	**neurofibromatosis** type 1 in genetic counseling practice: recommendations of the national society of genetic counselors	**9**	**-**	**-**	-	-
29	24637303	42.01	syndromic disorders with short stature	**-**	-	**10**	**-**	**50**
30	38533410	42.01	epidemiology and outcomes of **neurofibromatosis** type 1 (**nf-1**): multicenter tertiary experience	**10**	-	-	-	-

Table S5: Overview of the representation of each tested RASopathy in the scientific literature as of March 6, 2025. Total number of articles mentioning the syndrome name in the title were quantified for both PubMed (abstracts) and PubMed Central (articles), for each of the tested RASopathies: Neurofibromatosis type-1 (NF1), Neurofibromatosis-Noonan (NFNS), Noonan, Costello and Cardiofaciocutaneous (CFC) syndromes.

**Mentions in title**	**NF1**	**NFNS**	**Noonan**	**Costello**	**CFC**
**PubMed**	10272	31	1738	370	214
**PubMed Central**	2744	10	436	95	32

Table S6: Ontological semantic similarities obtained from phenotypic profiles comparison between the five analyzed RASopathies: Neurofibromatosis type-1 (NF1), Neurofibromatosis-Noonan, Noonan (NFNS), Costello and Cardiofaciocutaneous (CFC) syndromes. Similarity values range from 0 to 1, with higher values indicating greater similarity.

**Syndrome**	**NF1**	**NFNS**	**Noonan**	**Costello**	**CFC**
**NF1**	1.0	0.73	0.61	0.58	0.56
**NFNS**	0.73	1.0	0.65	0.61	0.62
**Noonan**	0.61	0.65	1.0	0.61	0.64
**Costello**	0.58	0.61	0.61	1.0	0.7
**CFC**	0.56	0.62	0.64	0.7	1.0

Table S7: Top 50 most similar abstracts to Neurofibromatosis type-1 (NF1) syndrome. For each document, the rank(R), the PubMed identifier (PMID), the ontological semantic similarity (Sim) and the title of the document are shown. Bold type in the title field indicates that the word is the disease name or any of the four other syndromes tested in the work or a related RASopathy (as Legius or Leopard), the words RASopathy or rare disease, or the nf1 gene. Long titles that do not refer to some of the words highlighted in bold have been trimmed. When a downloaded document lacked the title field, none is shown.

R	PMID	Sim	Title
1	3145091	0.36	von recklinghausen **neurofibromatosis**. a clinical and population study in south-east wales.
2	32393377	0.32	jaffe-campanacci syndrome or **neurofibromatosis** type 1: a case report of phenotypic overlap with detection of **nf1** gene mutation in non-ossifying fibroma.
3	37993698	0.31	pathogenesis and treatment of a giant occipital bone defect with meningoencephalocele in an **nf1** child: case report and review of the literature.
4	10629698	0.27	[clinical diagnosis of **neurofibromatosis** type 1].
5	37968860	0.27	neurofibroma: case series with clinical features and recommendations.
6	18183640	0.27	[**neurofibromatosis** type 1 and associated clinical abnormalities in 27 children].
7	11320736	0.26	[von recklinghausen's **neurofibromatosis** (**neurofibromatosis** type i)–a familial case report].
8	6417723	0.26	[radiological findings in recklinghausen's **neurofibromatosis**].
9	18505544	0.26	**leopard** syndrome.
10	20687928	0.25	monozygotic twins with **neurofibromatosis** type 1, concordant phenotype and synchronous development of mpnst and metastasis.
11	2518324	0.25	incidence, types, and management of cancer in patients with **neurofibromatosis**.
12	35749292	0.25	**neurofibromatosis** from head to toe: what the radiologist needs to know.
13	6412555	0.25	**neurofibromatosis** and secondary acoustic neuroma.
14	21909069	0.24	[**neurofibromatosis** type 1 and hypertension in pediatrics: case report].
15	31703719	0.24	**nf1** microdeletion syndrome: case report of two new patients.
16	17401183	0.24	[**neurofibromatosis**–an inborn genetic disorder with susceptibility to neoplasia].
17	18282435	0.23	[managing children with **neurofibromatosis** type 1: what should we look for?].
18	12397273	0.23	molecular analysis of diminutive, flat, depressed colorectal lesions: are they precursors of(...)
19	23244495	0.23	genotype-phenotype associations in **neurofibromatosis** type 1 (**nf1**): an increased risk of tumor complications in patients with **nf1** splice-site mutations?
20	26432421	0.23	whole-exome sequencing of breast cancer, malignant peripheral nerve sheath tumor and neurofibroma from a patient with **neurofibromatosis** type 1.
21	11952554	0.23	epidermodysplasia verruciformis associated with **neurofibromatosis** type 1: coincidental association or model for understanding the underlying mechanism of the disease?
22	18663272	0.23	[**neurofibromatosis** type 1 in children. experiences of the gdansk paediatric oncohaematology centre. preliminary results].
23	26564071	0.23	**neurofibromatosis** type 1.
24	34199217	0.23	severe phenotype in patients with large deletions of **nf1** .
25	12094380	0.22	nup88 (karyoporin) in human malignant neoplasms and dysplasias: correlations of immunostaining(...)
26	18214791	0.22	**neurofibromatosis** type i: genetics and clinical manifestations.
27	8491109	0.22	visual diagnosis of hematologic and oncologic diseases.
28	18000987	0.22	brain anomalies in encephalocraniocutaneous lipomatosis.
29	34624850	0.22	factors associated with early shunt revision within 30 days: analyses from the national surgical(...)
30	8212399	0.22	[recklinghausen **neurofibromatosis** in children].
31	37477762	0.22	prame immunohistochemistry in soft tissue tumors and mimics: a study of 350 cases highlighting(...)
32	1770531	0.22	**watson** syndrome: is it a subtype of type 1 **neurofibromatosis**?
33	23463485	0.22	evaluation of tibial osteopathy occurrence in **neurofibromatosis** type 1 italian patients.
34	35005728	0.22	**neurofibromatosis** type 1 in children: a single-center experience.
35	15635820	0.21	autoimmune haemolysis as an unusual cause of anaemia in von recklinghausen's disease.
36	16821075	0.21	hemimegalencephaly: clinical implications and surgical treatment.
37	9668168	0.21	selective disactivation of neurofibromin gap activity in **neurofibromatosis** type 1.
38	11259705	0.21	unusual lesions of the cerebellopontine angle: a segmental approach.
39	20027112	0.21	clinical and genetic aspects of **neurofibromatosis** 1.
40	15287415	0.21	a review of 35 cases of asymmetric crying facies.
41	16828412	0.21	rare syndromes.
42	35093157	0.21	whole-body mri evaluation in **neurofibromatosis** type 1 patients younger than 3 years old and the genetic contribution to disease progression.
43	28951274	0.21	spinal metastasis of unknown primary accompanied by neurologic deficit or vertebral instability.
44	23591128	0.21	cockayne syndrome pathogenesis: lessons from mouse models.
45	15150783	0.21	unusual features in a patient with **neurofibromatosis** type 1: multiple subcutaneous lipomas, a juvenile polyp in ascending colon, congenital intrahepatic portosystemic venous shunt, and horseshoe kidney.
46	19452429	0.21	perinatal (fetal and neonatal) tuberous sclerosis: a review.
47	27709959	0.21	none
48	28230061	0.21	**neurofibromatosis** type 1.
49	28296747	0.21	adult-onset still's disease with atypical cutaneous manifestations.
50	1795416	0.21	huge plexiform neurofibroma of the head and liver–case report.

Table S8: Top 50 most similar articles to Neurofibromatosis type-1 (NF1) syndrome. For each document, the rank(R), the PubMed identifier (PMID), the ontological semantic similarity (Sim) and the title of the document are shown. Bold type in the title field indicates that the word is the disease name or any of the four other syndromes tested in the work or a related RASopathy (as Legius or Leopard), the words RASopathy or rare disease, or the nf1 gene. Long titles that do not refer to some of the words highlighted in bold have been trimmed. When a downloaded document lacked the title field, none is shown.

R	PMID	Sim	Title
1	29290338	0.64	genotype-phenotype correlation in **nf1**: evidence for a more severe phenotype associated with missense mutations affecting **nf1** codons 844–848
2	26178382	0.63	high incidence of **noonan** syndrome features including short stature and pulmonic stenosis in patients carrying **nf1** missense mutations affecting p.arg1809: genotype–phenotype correlation
3	25061003	0.63	question sets and answers
4	19706204	0.62	encyclopaedia of tumour-associated familial disorders. part i: from aimah to chime syndrome
5	38680880	0.62	**neurofibromatosis** type 1: optimizing management with a multidisciplinary approach
6	35096710	0.61	diagnostic approach to macrocephaly in children
7	34199217	0.6	severe phenotype in patients with large deletions of **nf1**
8	34352903	0.6	**rare disease**s of the orbit
9	17636453	0.57	**neurofibromatosis** type 1 in genetic counseling practice: recommendations of the national society of genetic counselors
10	38533410	0.57	epidemiology and outcomes of **neurofibromatosis** type 1 (**nf-1**): multicenter tertiary experience
11	32014052	0.56	**neurofibromatosis** 1 french national guidelines based on an extensive literature review since 1966
12	35069816	0.56	orthopaedic manifestations of **neurofibromatosis** type 1: a case report
13	23793209	0.56	**neurofibromatosis**: chronological history and current issues
14	33919865	0.55	genotype-phenotype correlations in **neurofibromatosis** type 1: a single-center cohort study
15	27933191	0.55	genetics of inherited cardiocutaneous syndromes: a review
16	35892827	0.54	genetic disorders with predisposition to paediatric haematopoietic malignancies—a review
17	36831560	0.53	**neurofibromatosis** type 1: pediatric aspects and review of genotype–phenotype correlations
18	28573359	0.53	proceedings of the rami intern section meeting
19	34322401	0.52	curriculum for masters in general practice – bhutan
20	26110128	0.51	tumor-suppressor genes, cell cycle regulatory checkpoints, and the skin
21	34712008	0.51	international harmonization of nomenclature and diagnostic criteria (inhand): non-proliferative(...)
22	37496887	0.51	pathology competencies in medical education and educational cases: update 2023
23	25276129	0.5	adrenal disorders and the paediatric brain: pathophysiological considerations and clinical implications
24	30190611	0.5	expanding the clinical phenotype of individuals with a 3-bp in-frame deletion of the **nf1** gene (c.2970_2972del): an update of genotype–phenotype correlation
25	34544639	0.49	skin manifestations associated with systemic diseases – part ii
26	35885913	0.49	genotype-phenotype correlations in **neurofibromatosis** type 1: identification of novel and recurrent **nf1** gene variants and correlations with neurocognitive phenotype
27	34159396	0.49	cutaneous expression of familial cancer syndromes
28	34351021	0.49	2021 acvim forum research abstract program
29	32393377	0.49	jaffe-campanacci syndrome or **neurofibromatosis** type 1: a case report of phenotypic overlap with detection of **nf1** gene mutation in non-ossifying fibroma
30	37073110	0.49	mutational spectrum and genotype-phenotype correlations in **neurofibromatosis** type 1 patients from north macedonia: identification of ten novel **nf1** pathogenic variants
31	34202860	0.49	rubinstein-taybi syndrome: a model of epigenetic disorder
32	32147744	0.48	**legius** syndrome and its relationship with **neurofibromatosis** type 1
33	36803953	0.48	**neurofibromatosis**-**noonan** syndrome and growth deficiency in an iranian girl due to a pathogenic variant in **nf1** gene
34	29618358	0.48	clinical and molecular characterization of 112 single-center patients with **neurofibromatosis** type 1
35	34168676	0.48	genotype-phenotype associations in patients with type-1, type-2, and atypical **nf1** microdeletions
36	31703719	0.48	none
37	35200700	0.48	cardiomyopathies in children and systemic disorders when is it useful to look beyond the heart?
38	37239385	0.47	hereditary cancer syndromes: a comprehensive review with a visual tool
39	35382488	0.47	giant cell lesions of the jaws involving **rasopathy** syndromes
40	36091172	0.47	fanconi anemia and dyskeratosis congenita/telomere biology disorders: two inherited bone marrow(...)
41	29552546	0.47	update on genetic conditions affecting the skin and the kidneys
42	19032739	0.47	nevoid basal cell carcinoma syndrome (gorlin syndrome)
43	24767283	0.46	**leopard** syndrome: clinical dilemmas in differential diagnosis of **rasopathies**
44	37191267	0.46	isev2023 abstract book
45	31752929	0.46	the impact of **rasopathy**-associated mutations on cns development in mice and humans
46	29311971	0.46	update on 13 syndromes affecting craniofacial and dental structures
47	34405046	0.45	an update on fanconi anemia: clinical, cytogenetic and molecular approaches (review)
48	36339399	0.45	genetic conditions of short stature: a review of three classic examples
49	37576110	0.45	biallelic truncating variants in children with titinopathy represent a recognizable condition(...)
50	30158741	0.45	nonproliferative and proliferative lesions of the ratand mouse special sense organs(ocular [eye(...)

Table S9: Top 50 most similar abstracts to Neurofibromatosis-Noonan syndrome (NFNS). For each document, the rank(R), the PubMed identifier (PMID), the ontological semantic similarity (Sim) and the title of the document are shown. Bold type in the title field indicates that the word is the disease name or any of the four other syndromes tested in the work or a related RASopathy (as Legius or Leopard), the words RASopathy or rare disease, or the nf1 gene. Long titles that do not refer to some of the words highlighted in bold have been trimmed. When a downloaded document lacked the title field, none is shown.

R	PMID	Sim	Title
1	17100198	0.38	partial trisomy 1p (1p36.22–¿pter) and partial monosomy 9p (9p22.2–¿pter) associated with(...)
2	20814946	0.37	tissue-limited mosaicism for monosomy 13.
3	26852509	0.37	clinical report of a patient with de novo trisomy 12q23.1q24.33.
4	14564158	0.36	teebi hypertelorism syndrome.
5	9783713	0.34	further evidence for the involvement of human chromosome 6p24 in the aetiology of orofacial(...)
6	561886	0.33	[the 18p-syndrome (author's transl)].
7	12364941	0.33	king-denborough syndrome: report of two brazilian cases.
8	29061174	0.33	de novo chromosome 7q36.1q36.2 triplication in a child with developmental delay, growth failure,(...)
9	12940461	0.33	lymphstasis in a boy with **noonan** syndrome: implication for the development of skeletal features.
10	19267933	0.32	jacobsen syndrome.
11	7943044	0.32	new x-linked mental retardation (xlmr) syndrome with distinct facial appearance and growth retardation.
12	26893599	0.31	identification of 1p36 deletion syndrome in patients with facial dysmorphism and developmental(...)
13	23342975	0.31	bilateral radial agenesis with absent thumbs, complex heart defect, short stature, and facial(...)
14	34481514	0.31	cytogenomic characterization of a de novo 4q34.1 deletion in a girl with mild dysmorphic features(...)
15	33334799	0.31	the first korean case of baraitser-winter cerebro-fronto-facial syndrome with a novel mutation(...)
16	32467733	0.31	report of trisomy 2q34-qter and monosomy 4q35.2-qter in a child with mild dysmorphic syndrome(...)
17	18505544	0.31	**leopard** syndrome.
18	6711615	0.31	brief clinical report: duplication of distal 17q: report of an observation.
19	500089	0.3	partial duplication of the long arm of chromosome 5: a case due to balanced paternal translocation(...)
20	24734163	0.3	mosaic and partial monosomy of chromosome 21 in a case with low platelets count.
21	3901750	0.3	deficiency of chromosome 8p21.1—-8pter: case report and review of the literature.
22	1872531	0.29	[4p trisomy secondary to paternal translocation t(4p-;15q+)].
23	1184398	0.29	trisomy of the distal third of the long arm of chromosome 10. report of a new case due to a(...)
24	21464791	0.29	orbital manifestations of **noonan** syndrome.
25	3177454	0.29	a new x-linked dysplasia gigantism syndrome: follow up in the first family and report on a second(...)
26	11311002	0.29	a new syndrome with craniofacial and skeletal dysmorphisms and developmental delay.
27	3359688	0.29	distal trisomy of chromosome 17q due to inverted tandem duplication.
28	12558112	0.29	small terminal 10q26 deletion in a male patient with **noonan**-like stigmata: diagnosis by cytogenetic and fish analysis.
29	25804019	0.29	a patient with duplication (7)(p15.3p22.3) and deletion (7)(p22.3pter) characterized by array-cgh.
30	24826207	0.29	pallister-killian syndrome.
31	23072190	0.28	partial monosomy 3p (3p26.2 –¿ pter) and partial trisomy 5q (5q34 –¿ qter) in a girl with(...)
32	30204959	0.28	partial trisomy 5p12-q 11.2 resulting from a marker chromosome: a new case report with attention(...)
33	1200027	0.28	chromosome 3 duplication q21 leads to qter deletion p25 leads to pter syndrome in children of(...)
34	1776627	0.28	new syndrome: mother and son with hypertelorism, downslanting palpebral fissures, malar hypoplasia,(...)
35	19842870	0.28	neuroblastoma in a patient with coffin-siris syndrome.
36	7151837	0.28	two chromosomal syndromes in the same family: monosomy and trisomy for part of the short arm(...)
37	35707596	0.28	a new case of rare microdeletion 10q22.3q23 along with mosaic klinefelter syndrome associated(...)
38	7154048	0.28	the phenotype of ring chromosome 3.
39	37968860	0.28	neurofibroma: case series with clinical features and recommendations.
40	23608969	0.28	combined dup(7)(q22.1q32.2), inv(7)(q31.31q31.33), and ins(7;19)(q22.1;p13.2p13.2) in a 12-year-old(...)
41	1887854	0.28	trisomy 14 mosaicism in a 5-year-old boy.
42	27192892	0.28	a molecularly characterized interstitial deletion encompassing the 11q14.1-q23.3 region in a(...)
43	32357851	0.28	chinese patient with **neurofibromatosis**-**noonan** syndrome caused by novel heterozygous **nf1** exons 1-58 deletion: a case report.
44	314266	0.28	[trisomy 13qter by tandem duplication 46, xx, dir dup 13 (q21 qter), 9qh+].
45	8779319	0.28	new syndrome?: mca/mr syndrome with multiple circumferential skin creases.
46	9249870	0.28	prenatal diagnosis of partial trisomy 12 and partial trisomy 21 due to a 3:1 segregation of(...)
47	16353244	0.28	familial complex 3q;10q rearrangement unraveled by subtelomeric fish analysis.
48	3145091	0.28	von recklinghausen **neurofibromatosis**. a clinical and population study in south-east wales.
49	19233320	0.28	early detection of chromosome 9q22.32q31.1 microdeletion and the nevoid basal cell carcinoma(...)
50	22216833	0.27	chromosome 15q24 microdeletion syndrome.

Table S10: Top 50 most similar articles to Neurofibromatosis-Noonan syndrome (NFNS). For each document, the rank(R), the PubMed identifier (PMID), the ontological semantic similarity (Sim) and the title of the document are shown. Bold type in the title field indicates that the word is the disease name or any of the four other syndromes tested in the work or a related RASopathy (as Legius or Leopard), the words RASopathy or rare disease, or the nf1 gene. Long titles that do not refer to some of the words highlighted in bold have been trimmed. When a downloaded document lacked the title field, none is shown.

R	PMID	Sim	Title
1	29290338	0.72	genotype-phenotype correlation in **nf1**: evidence for a more severe phenotype associated with missense mutations affecting **nf1** codons 844–848
2	26178382	0.69	high incidence of **noonan** syndrome features including short stature and pulmonic stenosis in patients carrying **nf1** missense mutations affecting p.arg1809: genotype–phenotype correlation
3	35382488	0.69	giant cell lesions of the jaws involving **rasopathy** syndromes
4	27933191	0.69	genetics of inherited cardiocutaneous syndromes: a review
5	34199217	0.68	severe phenotype in patients with large deletions of **nf1**
6	35892827	0.66	genetic disorders with predisposition to paediatric haematopoietic malignancies—a review
7	29311971	0.65	update on 13 syndromes affecting craniofacial and dental structures
8	37073110	0.64	mutational spectrum and genotype-phenotype correlations in **neurofibromatosis** type 1 patients from north macedonia: identification of ten novel **nf1** pathogenic variants
9	24767283	0.63	**leopard** syndrome: clinical dilemmas in differential diagnosis of **rasopathies**
10	35096710	0.63	diagnostic approach to macrocephaly in children
11	19706204	0.63	encyclopaedia of tumour-associated familial disorders. part i: from aimah to chime syndrome
12	35200700	0.63	cardiomyopathies in children and systemic disorders when is it useful to look beyond the heart?
13	34564083	0.63	the role of rna-binding proteins in vertebrate neural crest and craniofacial development
14	38540405	0.62	trisomy 22 mosaicism from prenatal to postnatal findings: a case series and systematic review(...)
15	34073526	0.62	pallister–killian syndrome versus trisomy 12p—a clinical study of 5 new cases and a literature(...)
16	34352903	0.62	**rare disease**s of the orbit
17	19490664	0.6	subtelomeric fish analysis in 76 patients with syndromic developmental delay/intellectual disability
18	34202860	0.6	rubinstein-taybi syndrome: a model of epigenetic disorder
19	25395808	0.59	chromosomal and multifactorial genetic disorders with oral manifestations
20	34682127	0.59	evaluation and risk assessment of congenital anomalies in neonates
21	30190611	0.59	expanding the clinical phenotype of individuals with a 3-bp in-frame deletion of the **nf1** gene (c.2970_2972del): an update of genotype–phenotype correlation
22	17177115	0.59	**noonan** syndrome and related disorders: alterations in growth and puberty
23	29469822	0.58	autosomal recessive **noonan** syndrome associated with biallelic lztr1 variants
24	26758488	0.58	growth hormone deficiency in a child with **neurofibromatosis**-**noonan** syndrome
25	34405046	0.58	an update on fanconi anemia: clinical, cytogenetic and molecular approaches (review)
26	30429698	0.57	craniofacial and cutaneous findings in **noonan**, **costello** and **leopard** syndromes
27	36803953	0.56	**neurofibromatosis**-**noonan** syndrome and growth deficiency in an iranian girl due to a pathogenic variant in **nf1** gene
28	37576110	0.56	biallelic truncating variants in children with titinopathy represent a recognizable condition(...)
29	38673696	0.56	advancing the clinical and molecular understanding of cornelia de lange syndrome: a multidisciplinary(...)
30	32736638	0.55	multisystemic manifestations in a cohort of 75 classical ehlers-danlos syndrome patients: natural(...)
31	38680880	0.55	**neurofibromatosis** type 1: optimizing management with a multidisciplinary approach
32	22121397	0.55	dubowitz syndrome: a review and implications for cognitive, behavioral, and psychological features
33	38254922	0.54	a novel homozygous loss-of-function variant in spred2 causes autosomal recessive **noonan**-like syndrome
34	22188645	0.54	mechanisms of ring chromosome formation, ring instability and clinical consequences
35	37957608	0.54	occurrence of mosaic trisomy 22 and pericentric inversion of chromosome 9 in a patient with(...)
36	38136934	0.54	the **cardiofaciocutaneous** syndrome: from genetics to prognostic–therapeutic implications
37	24892279	0.54	dubowitz syndrome is a complex comprised of multiple, genetically distinct and phenotypically(...)
38	35053723	0.53	microcephaly in neurometabolic diseases
39	38622524	0.53	prenatal diagnosis of 18p deletion and 8p trisomy syndrome: literature review and report of(...)
40	22815589	0.53	the genus cladosporium
41	23691379	0.53	**neurofibromatosis** type 1 associated with hashimoto's thyroiditis: coincidence or possible link
42	35348676	0.53	expanding the molecular spectrum of pathogenic shoc2 variants underlying mazzanti syndrome
43	34712008	0.52	international harmonization of nomenclature and diagnostic criteria (inhand): non-proliferative(...)
44	22090712	0.52	craniosynostosis genetics: the mystery unfolds
45	38566929	0.52	craniofacial disorders and dysplasias: molecular, clinical, and management perspectives
46	31703719	0.52	none
47	28895939	0.52	sleep disorders in childhood neurogenetic disorders
48	38673496	0.52	clinical and genetic correlation in neurocristopathies: bridging a precision medicine gap
49	35205257	0.52	expanding the clinical phenotype of 19q interstitial deletions: a new case with 19q13.32-q13.33(...)
50	19284615	0.52	identification of subtelomeric genomic imbalances and breakpoint mapping with quantitative pcr(...)

Table S11: Top 50 most similar abstracts to Noonan syndrome. For each document, the rank(R), the PubMed identifier (PMID), the ontological semantic similarity (Sim) and the title of the document are shown. Bold type in the title field indicates that the word is the disease name or any of the four other syndromes tested in the work or a related RASopathy (as Legius or Leopard), the words RASopathy or rare disease, or the nf1 gene. Long titles that do not refer to some of the words highlighted in bold have been trimmed. When a downloaded document lacked the title field, none is shown.

R	PMID	Sim	Title
1	12940461	0.33	lymphstasis in a boy with **noonan** syndrome: implication for the development of skeletal features.
2	1200027	0.28	chromosome 3 duplication q21 leads to qter deletion p25 leads to pter syndrome in children of(...)
3	34636477	0.27	whole-exome sequencing identified first homozygous frameshift variant in the colec10 gene in(...)
4	12558112	0.27	small terminal 10q26 deletion in a male patient with **noonan**-like stigmata: diagnosis by cytogenetic and fish analysis.
5	18505544	0.26	**leopard** syndrome.
6	7151837	0.26	two chromosomal syndromes in the same family: monosomy and trisomy for part of the short arm(...)
7	8779319	0.26	new syndrome?: mca/mr syndrome with multiple circumferential skin creases.
8	561886	0.25	[the 18p-syndrome (author's transl)].
9	26852509	0.24	clinical report of a patient with de novo trisomy 12q23.1q24.33.
10	29061174	0.24	de novo chromosome 7q36.1q36.2 triplication in a child with developmental delay, growth failure,(...)
11	32467733	0.24	report of trisomy 2q34-qter and monosomy 4q35.2-qter in a child with mild dysmorphic syndrome(...)
12	21416592	0.23	how many entities exist for the spectrum of disorders associated with brachydactyly, syndactyly,(...)
13	26043504	0.23	a fertile patient with 45x/47xxx mosaicism.
14	7450776	0.23	the saethre-chotzen syndrome with partial bifid of the distal phalanges of the great toes. observations(...)
15	20503305	0.23	a new ehlers-danlos syndrome with craniofacial characteristics, multiple congenital contractures,(...)
16	2264484	0.23	45,xo/46,xy in a newborn with the stigmata of turner syndrome: report of one case.
17	3177454	0.22	a new x-linked dysplasia gigantism syndrome: follow up in the first family and report on a second(...)
18	27192892	0.22	a molecularly characterized interstitial deletion encompassing the 11q14.1-q23.3 region in a(...)
19	34481514	0.22	cytogenomic characterization of a de novo 4q34.1 deletion in a girl with mild dysmorphic features(...)
20	7506965	0.22	marden-walker syndrome: a case report and a critical review of the literature.
21	17716964	0.22	cytogenetic investigation of a child with a mosaic isochromosome 18q and ring 18q.
22	15770132	0.22	case report: y;6 translocation with deletion of 6p.
23	32949109	0.22	blepharophimosis-ptosis-intellectual disability syndrome: a report of nine egyptian patients(...)
24	14564158	0.22	teebi hypertelorism syndrome.
25	17100198	0.21	partial trisomy 1p (1p36.22–¿pter) and partial monosomy 9p (9p22.2–¿pter) associated with(...)
26	24826207	0.21	pallister-killian syndrome.
27	12101870	0.21	multiple congenital anomalies in association with supernumerary chromosome 47 inherited from(...)
28	30092813	0.21	smith-lemli-opitz syndrome presenting as acute adrenal crisis in a child: a case report.
29	34999713	0.21	**noonan** syndrome presenting with stunted growth: a case report.
30	12364941	0.21	king-denborough syndrome: report of two brazilian cases.
31	32006683	0.21	recessive marfanoid syndrome with herniation associated with a homozygous mutation in fibulin-3.
32	26518681	0.21	a novel heterozygous rit1 mutation in a patient with **noonan** syndrome, leukopenia, and transient myeloproliferation-a review of the literature.
33	7943044	0.21	new x-linked mental retardation (xlmr) syndrome with distinct facial appearance and growth retardation.
34	14564163	0.21	a patient with extensive blue rubber bleb nevi associated with multiple congenital anomalies:(...)
35	20814946	0.21	tissue-limited mosaicism for monosomy 13.
36	3734522	0.21	partial trisomy for the distal part of the long arm of chromosome 15–a new syndrome?
37	3276203	0.21	the lenz microphthalmia syndrome.
38	23565479	0.2	charge association.
39	7124793	0.2	limb pterygium syndromes: a review and report of eleven patients.
40	34956087	0.2	clinical characteristics and long-term recombinant human growth hormone treatment of 18q- syndrome:(...)
41	18348260	0.2	duplication of chromosome band 12q24.11q24.23 results in apparent **noonan** syndrome.
42	21857505	0.2	trisomy 14 mosaicism: clinical and cytogenetic findings in an adult.
43	3856414	0.2	cat's eye syndrome with cleft soft palate.
44	19842870	0.2	neuroblastoma in a patient with coffin-siris syndrome.
45	25076975	0.2	a chromosome 1q44 deletion in a 4-month-old girl; the first report in korea.
46	11883867	0.2	abnormalities of gh secretion in a young girl with floating-harbor syndrome.
47	10665663	0.2	molecular genetics of turner syndrome: correlation with clinical phenotype and response to growth(...)
48	1887854	0.2	trisomy 14 mosaicism in a 5-year-old boy.
49	9783713	0.2	further evidence for the involvement of human chromosome 6p24 in the aetiology of orofacial(...)
50	25230004	0.2	19q13.32 microdeletion syndrome: three new cases.

Table S12: Top 50 most similar articles to Noonan syndrome. For each document, the rank(R), the PubMed identifier (PMID), the ontological semantic similarity (Sim) and the title of the document are shown. Bold type in the title field indicates that the word is the disease name or any of the four other syndromes tested in the work or a related RASopathy (as Legius or Leopard), the words RASopathy or rare disease, or the nf1 gene. Long titles that do not refer to some of the words highlighted in bold have been trimmed. When a downloaded document lacked the title field, none is shown.

R	PMID	Sim	Title
1	27933191	0.64	genetics of inherited cardiocutaneous syndromes: a review
2	35200700	0.58	cardiomyopathies in children and systemic disorders when is it useful to look beyond the heart?
3	17222357	0.57	**noonan** syndrome
4	38540405	0.56	trisomy 22 mosaicism from prenatal to postnatal findings: a case series and systematic review(...)
5	24083159	0.56	the other side of turner's: **noonan**'s syndrome
6	34352903	0.54	**rare disease**s of the orbit
7	32736638	0.53	multisystemic manifestations in a cohort of 75 classical ehlers-danlos syndrome patients: natural(...)
8	35205257	0.51	expanding the clinical phenotype of 19q interstitial deletions: a new case with 19q13.32-q13.33(...)
9	35892827	0.51	genetic disorders with predisposition to paediatric haematopoietic malignancies—a review
10	24637303	0.51	syndromic disorders with short stature
11	25061003	0.5	question sets and answers
12	36339399	0.5	genetic conditions of short stature: a review of three classic examples
13	34202860	0.49	rubinstein-taybi syndrome: a model of epigenetic disorder
14	27125300	0.49	the growth characteristics of patients with **noonan** syndrome: results of three years of growth hormone treatment: a nationwide multicenter study
15	22121397	0.49	dubowitz syndrome: a review and implications for cognitive, behavioral, and psychological features
16	25395808	0.49	chromosomal and multifactorial genetic disorders with oral manifestations
17	24754368	0.48	twin infant with lymphatic dysplasia diagnosed with **noonan** syndrome by molecular genetic testing
18	34073526	0.48	pallister–killian syndrome versus trisomy 12p—a clinical study of 5 new cases and a literature(...)
19	38673696	0.48	advancing the clinical and molecular understanding of cornelia de lange syndrome: a multidisciplinary(...)
20	37443678	0.48	marfan syndrome: enhanced diagnostic tools and follow-up management strategies
21	32973676	0.48	autoimmune thyroid disease in specific genetic syndromes in childhood and adolescence
22	34682127	0.48	evaluation and risk assessment of congenital anomalies in neonates
23	30429698	0.47	craniofacial and cutaneous findings in **noonan**, **costello** and **leopard** syndromes
24	38136934	0.47	the **cardiofaciocutaneous** syndrome: from genetics to prognostic–therapeutic implications
25	19706204	0.47	encyclopaedia of tumour-associated familial disorders. part i: from aimah to chime syndrome
26	17177115	0.47	**noonan** syndrome and related disorders: alterations in growth and puberty
27	29654333	0.46	level 3 guideline on the treatment of patients with severe/multiple injuries
28	28144274	0.46	**noonan** syndrome – a new survey
29	30054457	0.46	none
30	29469822	0.46	autosomal recessive **noonan** syndrome associated with biallelic lztr1 variants
31	35096710	0.46	diagnostic approach to macrocephaly in children
32	29311971	0.46	update on 13 syndromes affecting craniofacial and dental structures
33	17958891	0.45	mowat-wilson syndrome
34	36566191	0.45	new insights on **noonan** syndrome's clinical phenotype: a single center retrospective study
35	34322401	0.45	curriculum for masters in general practice – bhutan
36	35986401	0.45	cutis verticis gyrata and **noonan** syndrome: report of two cases with pathogenetic variant in sos1 gene
37	29670795	0.45	pulmonary vasculitis and a horseshoe kidney in **noonan** syndrome
38	38495807	0.44	best practice recommendations for infertility management
39	19490664	0.44	subtelomeric fish analysis in 76 patients with syndromic developmental delay/intellectual disability
40	32164556	0.44	molecular and clinical studies in 107 **noonan** syndrome affected individuals with ptpn11 mutations
41	35730652	0.44	the map3k7 gene: further delineation of clinical characteristics and genotype/phenotype correlations
42	36091172	0.44	fanconi anemia and dyskeratosis congenita/telomere biology disorders: two inherited bone marrow(...)
43	22815589	0.44	the genus cladosporium
44	34539746	0.44	congenital disorders of deficiency in glycosaminoglycan biosynthesis
45	34405046	0.44	an update on fanconi anemia: clinical, cytogenetic and molecular approaches (review)
46	37576110	0.44	biallelic truncating variants in children with titinopathy represent a recognizable condition(...)
47	28573359	0.44	proceedings of the rami intern section meeting
48	37957608	0.44	occurrence of mosaic trisomy 22 and pericentric inversion of chromosome 9 in a patient with(...)
49	26355968	0.43	guideline for care of patients with the diagnoses of craniosynostosis: working group on craniosynostosis
50	29290338	0.43	genotype-phenotype correlation in **nf1**: evidence for a more severe phenotype associated with missense mutations affecting **nf1** codons 844–848

Table S13: Top 50 most similar abstracts to Costello syndrome. For each document, the rank(R), the PubMed identifier (PMID), the ontological semantic similarity (Sim) and the title of the document are shown. Bold type in the title field indicates that the word is the disease name or any of the four other syndromes tested in the work or a related RASopathy (as Legius or Leopard), the words RASopathy or rare disease, or the nf1 gene. Long titles that do not refer to some of the words highlighted in bold have been trimmed. When a downloaded document lacked the title field, none is shown.

R	PMID	Sim	Title
1	16007634	0.27	**cardiofaciocutaneous** syndrome (**cfc**) with congenital peripheral neuropathy and nonorganic malnutrition: an autopsy study.
2	20503305	0.27	a new ehlers-danlos syndrome with craniofacial characteristics, multiple congenital contractures,(...)
3	12940461	0.23	lymphstasis in a boy with **noonan** syndrome: implication for the development of skeletal features.
4	561886	0.22	[the 18p-syndrome (author's transl)].
5	7151837	0.22	two chromosomal syndromes in the same family: monosomy and trisomy for part of the short arm(...)
6	1200027	0.22	chromosome 3 duplication q21 leads to qter deletion p25 leads to pter syndrome in children of(...)
7	17100198	0.22	partial trisomy 1p (1p36.22–¿pter) and partial monosomy 9p (9p22.2–¿pter) associated with(...)
8	17633324	0.22	[camptomelic dysplasia–a case report].
9	3177454	0.21	a new x-linked dysplasia gigantism syndrome: follow up in the first family and report on a second(...)
10	36935482	0.21	the phenotypic spectrum of terminal 6q deletions based on a large cohort derived from social(...)
11	15793836	0.21	a girl with inverted triplication of chromosome 3q25.3 –¿ q29 and multiple congenital anomalies(...)
12	17506107	0.21	expanding spectrum of congenital disorder of glycosylation ig (cdg-ig): sibs with a unique skeletal(...)
13	12101870	0.21	multiple congenital anomalies in association with supernumerary chromosome 47 inherited from(...)
14	17716964	0.2	cytogenetic investigation of a child with a mosaic isochromosome 18q and ring 18q.
15	20067885	0.2	prenatal evaluation of a fetus with trisomy 18 and additional balanced de novo rob(13;14).
16	29061174	0.2	de novo chromosome 7q36.1q36.2 triplication in a child with developmental delay, growth failure,(...)
17	8145689	0.2	[partial trisomy of the long arm of chromosome 6. a clinical case].
18	24826207	0.2	pallister-killian syndrome.
19	6507477	0.2	a "new" lethal multiple congenital anomaly syndrome: joint contractures, cerebellar hypoplasia,(...)
20	32467733	0.19	report of trisomy 2q34-qter and monosomy 4q35.2-qter in a child with mild dysmorphic syndrome(...)
21	20814946	0.19	tissue-limited mosaicism for monosomy 13.
22	15103720	0.19	proximal chromosome 8q deletion in a boy with femoral bifurcation and other multiple congenital(...)
23	25076975	0.19	a chromosome 1q44 deletion in a 4-month-old girl; the first report in korea.
24	3734522	0.19	partial trisomy for the distal part of the long arm of chromosome 15–a new syndrome?
25	18398855	0.19	schinzel-giedion syndrome: report of splenopancreatic fusion and proposed diagnostic criteria.
26	25804019	0.19	a patient with duplication (7)(p15.3p22.3) and deletion (7)(p22.3pter) characterized by array-cgh.
27	17850629	0.19	identification of proximal 1p36 deletions using array-cgh: a possible new syndrome.
28	15287415	0.19	a review of 35 cases of asymmetric crying facies.
29	25223409	0.19	trisomy 1q41-qter and monosomy 3p26.3-pter in a family with a translocation (1;3): further delineation(...)
30	33334799	0.19	the first korean case of baraitser-winter cerebro-fronto-facial syndrome with a novel mutation(...)
31	26893599	0.19	identification of 1p36 deletion syndrome in patients with facial dysmorphism and developmental(...)
32	19842870	0.18	neuroblastoma in a patient with coffin-siris syndrome.
33	23342975	0.18	bilateral radial agenesis with absent thumbs, complex heart defect, short stature, and facial(...)
34	2063901	0.18	digeorge anomaly in an infant with deletion of chromosome 22 and dup(9p) due to adjacent type(...)
35	10482872	0.18	apparent cyclophosphamide (cytoxan) embryopathy: a distinct phenotype?
36	30442170	0.18	ent and mucopolysaccharidoses.
37	14201252	0.18	the de lange syndrome: report of three cases.
38	22495892	0.18	neonatal lethal **costello** syndrome and unusual dinucleotide deletion/insertion mutations in hras predicting p.gly12val.
39	25345092	0.18	mucopolysaccharidosis type ii, hunter's syndrome.
40	3901750	0.18	deficiency of chromosome 8p21.1—-8pter: case report and review of the literature.
41	30092813	0.18	smith-lemli-opitz syndrome presenting as acute adrenal crisis in a child: a case report.
42	6974530	0.18	ring chromosome 11 [46,xx,r(11) (p15q25)] associated with clinical features of the 11q- syndrome.
43	14564158	0.18	teebi hypertelorism syndrome.
44	15770132	0.18	case report: y;6 translocation with deletion of 6p.
45	17502954	0.18	holt-oram syndrome.
46	9783713	0.18	further evidence for the involvement of human chromosome 6p24 in the aetiology of orofacial(...)
47	26852509	0.18	clinical report of a patient with de novo trisomy 12q23.1q24.33.
48	18505544	0.18	**leopard** syndrome.
49	8599356	0.18	infantile lethal variant of simpson-golabi-behmel syndrome associated with hydrops fetalis.
50	9932584	0.18	the charge association: the role of tracheotomy.

Table S14: Top 50 most similar articles to Costello syndrome. For each document, the rank(R), the PubMed identifier (PMID), the ontological semantic similarity (Sim) and the title of the document are shown. Bold type in the title field indicates that the word is the disease name or any of the four other syndromes tested in the work or a related RASopathy (as Legius or Leopard), the words RASopathy or rare disease, or the nf1 gene. Long titles that do not refer to some of the words highlighted in bold have been trimmed. When a downloaded document lacked the title field, none is shown.

R	PMID	Sim	Title
1	27933191	0.57	genetics of inherited cardiocutaneous syndromes: a review
2	25061003	0.56	question sets and answers
3	35677617	0.54	multidisciplinary management of **costello** syndrome: current perspectives
4	35096710	0.54	diagnostic approach to macrocephaly in children
5	19706204	0.54	encyclopaedia of tumour-associated familial disorders. part i: from aimah to chime syndrome
6	35200700	0.52	cardiomyopathies in children and systemic disorders when is it useful to look beyond the heart?
7	35205257	0.51	expanding the clinical phenotype of 19q interstitial deletions: a new case with 19q13.32-q13.33(...)
8	34073526	0.51	pallister–killian syndrome versus trisomy 12p—a clinical study of 5 new cases and a literature(...)
9	28573359	0.5	proceedings of the rami intern section meeting
10	25395808	0.5	chromosomal and multifactorial genetic disorders with oral manifestations
11	34352903	0.49	**rare disease**s of the orbit
12	34564083	0.49	the role of rna-binding proteins in vertebrate neural crest and craniofacial development
13	34712008	0.49	international harmonization of nomenclature and diagnostic criteria (inhand): non-proliferative(...)
14	35053723	0.47	microcephaly in neurometabolic diseases
15	30429698	0.46	craniofacial and cutaneous findings in **noonan**, **costello** and **leopard** syndromes
16	38540405	0.46	trisomy 22 mosaicism from prenatal to postnatal findings: a case series and systematic review(...)
17	30123105	0.46	mutational landscapes and phenotypic spectrum of swi/snf-related intellectual disability disorders
18	29469822	0.46	autosomal recessive **noonan** syndrome associated with biallelic lztr1 variants
19	24764755	0.46	new microdeletion and microduplication syndromes: a comprehensive review
20	34322401	0.46	curriculum for masters in general practice – bhutan
21	32736638	0.45	multisystemic manifestations in a cohort of 75 classical ehlers-danlos syndrome patients: natural(...)
22	29311971	0.45	update on 13 syndromes affecting craniofacial and dental structures
23	34682127	0.44	evaluation and risk assessment of congenital anomalies in neonates
24	37576110	0.44	biallelic truncating variants in children with titinopathy represent a recognizable condition(...)
25	37443678	0.44	marfan syndrome: enhanced diagnostic tools and follow-up management strategies
26	34544639	0.44	skin manifestations associated with systemic diseases – part ii
27	33805950	0.44	kabuki syndrome—clinical review with molecular aspects
28	22815589	0.44	the genus cladosporium
29	34202860	0.43	rubinstein-taybi syndrome: a model of epigenetic disorder
30	29654333	0.43	level 3 guideline on the treatment of patients with severe/multiple injuries
31	35892827	0.43	genetic disorders with predisposition to paediatric haematopoietic malignancies—a review
32	33194904	0.43	overgrowth syndromes—evaluation, diagnosis, and management
33	28895939	0.42	sleep disorders in childhood neurogenetic disorders
34	33827624	0.42	a framework for the evaluation of patients with congenital facial weakness
35	26355968	0.42	guideline for care of patients with the diagnoses of craniosynostosis: working group on craniosynostosis
36	32282878	0.42	bi-allelic gad1 variants cause a neonatal onset syndromic developmental and epileptic encephalopathy
37	38671997	0.42	behavioral and psychiatric disorders in syndromic autism
38	38136934	0.42	the **cardiofaciocutaneous** syndrome: from genetics to prognostic–therapeutic implications
39	37496887	0.42	pathology competencies in medical education and educational cases: update 2023
40	34815299	0.42	clinical and molecular features of 66 patients with musculocontractural ehlers-danlos syndrome(...)
41	35986401	0.41	cutis verticis gyrata and **noonan** syndrome: report of two cases with pathogenetic variant in sos1 gene
42	38566929	0.41	craniofacial disorders and dysplasias: molecular, clinical, and management perspectives
43	38673696	0.41	advancing the clinical and molecular understanding of cornelia de lange syndrome: a multidisciplinary(...)
44	19490664	0.41	subtelomeric fish analysis in 76 patients with syndromic developmental delay/intellectual disability
45	30394212	0.41	tall stature: a challenge for clinicians
46	17958891	0.41	mowat-wilson syndrome
47	37947243	0.41	2023 acvim forum research abstract program
48	37488489	0.4	fatal leukodystrophy in **costello** syndrome: a case report
49	28658095	0.4	a clinical review on megalencephaly
50	38495807	0.4	best practice recommendations for infertility management

Table S15: Top 50 most similar abstracts to CFC syndrome. For each document, the rank(R), the PubMed identifier (PMID), the ontological semantic similarity (Sim) and the title of the document are shown. Bold type in the title field indicates that the word is the disease name or any of the four other syndromes tested in the work or a related RASopathy (as Legius or Leopard), the words RASopathy or rare disease, or the nf1 gene. Long titles that do not refer to some of the words highlighted in bold have been trimmed. When a downloaded document lacked the title field, none is shown.

R	PMID	Sim	Title
1	16007634	0.29	**cardiofaciocutaneous** syndrome (**cfc**) with congenital peripheral neuropathy and nonorganic malnutrition: an autopsy study.
2	32467733	0.25	report of trisomy 2q34-qter and monosomy 4q35.2-qter in a child with mild dysmorphic syndrome(...)
3	20503305	0.24	a new ehlers-danlos syndrome with craniofacial characteristics, multiple congenital contractures,(...)
4	17100198	0.24	partial trisomy 1p (1p36.22–¿pter) and partial monosomy 9p (9p22.2–¿pter) associated with(...)
5	561886	0.23	[the 18p-syndrome (author's transl)].
6	1200027	0.23	chromosome 3 duplication q21 leads to qter deletion p25 leads to pter syndrome in children of(...)
7	14564158	0.22	teebi hypertelorism syndrome.
8	21416592	0.22	how many entities exist for the spectrum of disorders associated with brachydactyly, syndactyly,(...)
9	15036797	0.22	down syndrome: clinical profile from india.
10	751383	0.22	delineation of syndromes due to partial 6q imbalances. trisomy 6q21 leads to qter and monosomy(...)
11	7315873	0.22	the dup(3q) syndrome: report of eight cases and review of the literature.
12	16825433	0.21	the **cardiofaciocutaneous** syndrome.
13	21344634	0.21	a girl with metopic synostosis and trisomy 13 mosaicism: case report and review of the literature.
14	3177454	0.21	a new x-linked dysplasia gigantism syndrome: follow up in the first family and report on a second(...)
15	26264232	0.21	wac loss-of-function mutations cause a recognisable syndrome characterised by dysmorphic features,(...)
16	29061174	0.21	de novo chromosome 7q36.1q36.2 triplication in a child with developmental delay, growth failure,(...)
17	16353244	0.21	familial complex 3q;10q rearrangement unraveled by subtelomeric fish analysis.
18	25223409	0.21	trisomy 1q41-qter and monosomy 3p26.3-pter in a family with a translocation (1;3): further delineation(...)
19	12872809	0.21	partial monosomy 11q and trisomy 12q: variable expression in two siblings.
20	26086840	0.21	a homozygous b3gat3 mutation causes a severe syndrome with multiple fractures, expanding the(...)
21	20814946	0.21	tissue-limited mosaicism for monosomy 13.
22	5173168	0.21	unusual facies, joint hypermobility, genital anomaly and short stature: a new dysmorphic syndrome.
23	8779319	0.21	new syndrome?: mca/mr syndrome with multiple circumferential skin creases.
24	8145689	0.2	[partial trisomy of the long arm of chromosome 6. a clinical case].
25	6199974	0.2	terminal and interstitial deletions of the long arm of chromosome 7: a review with five new(...)
26	17716964	0.2	cytogenetic investigation of a child with a mosaic isochromosome 18q and ring 18q.
27	36935482	0.2	the phenotypic spectrum of terminal 6q deletions based on a large cohort derived from social(...)
28	19842870	0.2	neuroblastoma in a patient with coffin-siris syndrome.
29	33334799	0.2	the first korean case of baraitser-winter cerebro-fronto-facial syndrome with a novel mutation(...)
30	16691600	0.2	the fg syndrome: report of a large italian series.
31	7943044	0.2	new x-linked mental retardation (xlmr) syndrome with distinct facial appearance and growth retardation.
32	29142765	0.2	duplication of 19p13.3 in 11-year-old male patient with dysmorphic features and intellectual(...)
33	23342975	0.2	bilateral radial agenesis with absent thumbs, complex heart defect, short stature, and facial(...)
34	9249870	0.2	prenatal diagnosis of partial trisomy 12 and partial trisomy 21 due to a 3:1 segregation of(...)
35	26852509	0.2	clinical report of a patient with de novo trisomy 12q23.1q24.33.
36	15793836	0.2	a girl with inverted triplication of chromosome 3q25.3 –¿ q29 and multiple congenital anomalies(...)
37	19182162	0.19	multiple congenital anomalies/mental retardation syndrome with multiple circumferential skin(...)
38	27192892	0.19	a molecularly characterized interstitial deletion encompassing the 11q14.1-q23.3 region in a(...)
39	7151837	0.19	two chromosomal syndromes in the same family: monosomy and trisomy for part of the short arm(...)
40	12558112	0.19	small terminal 10q26 deletion in a male patient with **noonan**-like stigmata: diagnosis by cytogenetic and fish analysis.
41	17633324	0.19	[camptomelic dysplasia–a case report].
42	1819957	0.19	[fetal alcohol syndrome–case report].
43	14201252	0.19	the de lange syndrome: report of three cases.
44	9783713	0.19	further evidence for the involvement of human chromosome 6p24 in the aetiology of orofacial(...)
45	2178418	0.19	loss of the 3p25.3 band is critical in the manifestation of del(3p) syndrome: karyotype-phenotype(...)
46	9321771	0.19	interstitial duplication of the short arm of chromosome 2: report of a new case and review.
47	24767651	0.19	a 1.37-mb 12p11.22-p11.21 deletion coincident with a 367-kb 22q11.2 duplication detected by(...)
48	27617128	0.19	pitt-hopkins syndrome and differential diagnosis: a molecular and clinical challenge.
49	12364941	0.19	king-denborough syndrome: report of two brazilian cases.
50	16158441	0.19	ehlers-danlos syndrome type vib with characteristic facies, decreased curvatures of the spinal(...)

Table S16: Top 50 most similar articles to CFC syndrome. For each document, the rank(R), the PubMed identifier (PMID), the ontological semantic similarity (Sim) and the title of the document are shown. Bold type in the title field indicates that the word is the disease name or any of the four other syndromes tested in the work or a related RASopathy (as Legius or Leopard), the words RASopathy or rare disease, or the nf1 gene. Long titles that do not refer to some of the words highlighted in bold have been trimmed. When a downloaded document lacked the title field, none is shown.

R	PMID	Sim	Title
1	34352903	0.6	**rare disease**s of the orbit
2	27933191	0.57	genetics of inherited cardiocutaneous syndromes: a review
3	34564083	0.54	the role of rna-binding proteins in vertebrate neural crest and craniofacial development
4	25395808	0.54	chromosomal and multifactorial genetic disorders with oral manifestations
5	35205257	0.52	expanding the clinical phenotype of 19q interstitial deletions: a new case with 19q13.32-q13.33(...)
6	19706204	0.52	encyclopaedia of tumour-associated familial disorders. part i: from aimah to chime syndrome
7	34073526	0.51	pallister–killian syndrome versus trisomy 12p—a clinical study of 5 new cases and a literature(...)
8	32736638	0.51	multisystemic manifestations in a cohort of 75 classical ehlers-danlos syndrome patients: natural(...)
9	38136934	0.51	the **cardiofaciocutaneous** syndrome: from genetics to prognostic–therapeutic implications
10	35053723	0.5	microcephaly in neurometabolic diseases
11	25061003	0.5	question sets and answers
12	35200700	0.48	cardiomyopathies in children and systemic disorders when is it useful to look beyond the heart?
13	38540405	0.47	trisomy 22 mosaicism from prenatal to postnatal findings: a case series and systematic review(...)
14	38673696	0.47	advancing the clinical and molecular understanding of cornelia de lange syndrome: a multidisciplinary(...)
15	35096710	0.47	diagnostic approach to macrocephaly in children
16	24764755	0.46	new microdeletion and microduplication syndromes: a comprehensive review
17	30123105	0.46	mutational landscapes and phenotypic spectrum of swi/snf-related intellectual disability disorders
18	34712008	0.46	international harmonization of nomenclature and diagnostic criteria (inhand): non-proliferative(...)
19	22121397	0.46	dubowitz syndrome: a review and implications for cognitive, behavioral, and psychological features
20	38566929	0.46	craniofacial disorders and dysplasias: molecular, clinical, and management perspectives
21	30429698	0.45	craniofacial and cutaneous findings in **noonan**, **costello** and **leopard** syndromes
22	19267933	0.45	jacobsen syndrome
23	38711916	0.45	cytogenomic characterization of small supernumerary marker chromosomes in patients(...)
24	37443678	0.45	marfan syndrome: enhanced diagnostic tools and follow-up management strategies
25	35677617	0.45	multidisciplinary management of **costello** syndrome: current perspectives
26	30394212	0.45	tall stature: a challenge for clinicians
27	31557424	0.44	clinical and genetic analysis of zttk syndrome caused by son heterozygous mutation c.394c¿t
28	29311971	0.44	update on 13 syndromes affecting craniofacial and dental structures
29	32282878	0.44	bi-allelic gad1 variants cause a neonatal onset syndromic developmental and epileptic encephalopathy
30	37576110	0.44	biallelic truncating variants in children with titinopathy represent a recognizable condition(...)
31	22188645	0.44	mechanisms of ring chromosome formation, ring instability and clinical consequences
32	33982229	0.44	clinical characteristics of polish patients with molecularly confirmed mowat-wilson syndrome
33	34393548	0.43	manual of north american agromyzidae (diptera , schizophora), with revision of the fauna(...)
34	17958891	0.43	mowat-wilson syndrome
35	33827624	0.43	a framework for the evaluation of patients with congenital facial weakness
36	34202860	0.43	rubinstein-taybi syndrome: a model of epigenetic disorder
37	35892827	0.43	genetic disorders with predisposition to paediatric haematopoietic malignancies—a review
38	35716097	0.43	genotype–phenotype spectrum and correlations in xia-gibbs syndrome: report of five novel cases(...)
39	22815589	0.43	the genus cladosporium
40	34539746	0.43	congenital disorders of deficiency in glycosaminoglycan biosynthesis
41	27730037	0.43	**cardiofacio-cutaneous** syndrome: classical presentation of a rare genodermatoses
42	38671997	0.43	behavioral and psychiatric disorders in syndromic autism
43	35024745	0.43	**cardiofaciocutaneous** syndrome – a longitudinal study of a case over 33 years: case report and review of the literature
44	38673496	0.43	clinical and genetic correlation in neurocristopathies: bridging a precision medicine gap
45	30065609	0.42	x-linked sensorineural hearing loss: a literature review
46	32705777	0.42	phenotypic expansion in zhu-tokita-takenouchi-kim syndrome caused by de novo variants in the(...)
47	26242548	0.42	congenital generalized hypertrichosis: the skin as a clue to complex malformation syndromes
48	19490664	0.42	subtelomeric fish analysis in 76 patients with syndromic developmental delay/intellectual disability
49	30158741	0.42	nonproliferative and proliferative lesions of the ratand mouse special sense organs(ocular [eye(...)
50	24637303	0.42	syndromic disorders with short stature

Table S17: Ontological and textual similarity scores for the top 50 abstracts annotated by stEngine bi-encoder ranked for NF1

Phenotypes	3145091	32393377	37993698	10629698	37968860	18183640	11320736	6417723	18505544	20687928	2518324	35749292	6412555	21909069	31703719	17401183	18282435	12397273	23244495	26432421	11952554	18663272	26564071	34199217	12094380	18214791	8491109	18000987	34624850	8212399	37477762	1770531	23463485	35005728	15635820	16821075	9668168	11259705	20027112	15287415	16828412	35093157	28951274	23591128	15150783	19452429	27709959	28230061	28296747	1795416
Spinal neurofibroma	1.0	0.8	0.8	1.0	0.8	0.8	1.0	0.9	0.8	0.8	0.8	0.8	0.8	0.8	0.9	0.8	0.8	0.7	1.0	0.9	0.9	0.8	0.8	1.0	0.8	0.9	1.0	0.8	0.7	0.8	0.8	0.9	0.8	0.8	0.8	0.9	0.8	0.6	0.8	0.4	0.8	1.0	0.6		0.8	0.8	0.8	1.0	0.7	0.8
	0.8	0.8	0.7	0.9	0.8	0.8	0.9	0.7	0.8	0.9	0.8	0.7	0.9	0.8	0.7	0.9	0.9	0.8	0.8	0.7	0.7	0.8	0.8	0.7	0.8	0.8	1.0	0.8	0.7	0.7	0.9	0.8	0.8	0.8	0.7	0.8	0.8	0.9	0.8	0.8	0.8	0.8	0.8		0.8	0.8	0.8	0.9	0.7	0.8
Lisch nodules	1.0	1.0	1.0	1.0	1.0	0.6	1.0	0.5	0.4		1.0	1.0		1.0	0.3	1.0	1.0	0.3	1.0	1.0	1.0	1.0	1.0	1.0	0.5	1.0	0.7		0.4		0.8			1.0	1.0		1.0	0.8		0.6	1.0	0.5	0.5		1.0	0.8	0.5		0.5	1.0
	0.7	0.8	0.8	0.8	1.0	0.7	1.0	0.8	0.7		1.0	1.0		0.8	1.0	0.8	0.8	1.0	1.0	0.8	0.8	0.9	1.0	1.0	1.0	1.0	0.8		0.7		0.8			0.8	1.0		0.8	0.8		0.7	1.0	0.8	0.7		1.0	0.7	0.9		0.7	0.8
Plexiform neurofibroma	1.0	1.0	1.0	0.7	1.0	0.7	0.7	0.7	0.0	1.0	0.7	1.0	0.7	0.7		0.7	1.0		1.0	0.7	0.6	0.7	0.7	0.7	0.6	0.7	0.6				0.7		1.0	0.7	0.7	0.2		0.6	1.0	0.3	0.7	1.0	0.6		0.6	0.5	0.6	1.0	0.3	1.0
	0.9	0.9	0.9	0.8	1.0	0.8	0.7	0.8	0.7	0.8	0.7	0.9	0.9	0.8		0.8	1.0		0.9	0.8	0.8	0.8	0.8	0.8	1.0	0.8	0.8				0.7		1.0	0.8	0.8	0.7		0.8	0.9	0.8	0.8	0.9	0.8		0.8	0.7	1.0	0.8	0.8	0.8
Optic nerve glioma	1.0	1.0	0.5		1.0	1.0	1.0	1.0		0.5	0.8	1.0		1.0		1.0	1.0		1.0	0.8		0.8	0.8		0.8	1.0	0.5				0.6		1.0	1.0	1.0	0.8	1.0	0.8	1.0	0.3	0.5	1.0	0.7	0.8		0.5	0.8	1.0		0.8
	1.0	0.8	0.7		1.0	0.8	1.0	0.8		0.8	1.0	0.9		1.0		0.7	0.9		1.0	0.9		0.8	0.8		0.9	0.9	0.7				0.8		0.9	0.9	0.9	0.7	1.0	1.0	0.9	0.8	0.7	0.9	0.7	0.7		0.8	1.0	0.8		0.8
Neurofibrosarcoma	0.6	1.0	1.0	1.0	1.0		1.0			1.0	1.0	1.0	1.0			1.0	1.0		1.0	1.0	1.0		1.0	1.0	1.0	1.0	0.6			1.0	1.0			1.0			1.0	1.0	1.0	0.0	1.0	1.0	0.6		1.0		1.0	1.0		
	0.7	0.9	0.9	0.9	0.8		0.8			0.9	1.0	0.7	0.8			0.8	0.8		0.9	0.8	0.7		0.9	0.9	0.8	0.8	0.8			0.7	1.0			0.8			0.7	0.8	1.0	0.8	0.7	1.0	1.0		0.7		0.8	0.8		
Pheochromocytoma	0.8	0.6	0.6		0.6	0.9	0.8	0.8	0.4	0.7	0.9	0.9	0.6	0.9		0.6	0.6		0.9	0.9	0.8	0.6	0.9		0.8	0.6	0.8	0.3			0.7		0.6	0.6	0.6	0.2		1.0	0.7	0.8	0.9	0.7	0.7				0.8	0.6		0.4
	0.7	0.8	0.8		0.8	0.8	0.8	0.7	0.8	0.8	0.8	1.0	0.8	0.8		0.8	0.8		1.0	1.0	0.8	0.8	1.0		0.8	0.7	0.7	0.7			0.7		0.8	0.8	0.7	0.7		0.7	0.8	1.0	1.0	0.8	0.7				0.7	0.7		0.7
Few cafe-au-lait spots			0.9	1.0	1.0	1.0	0.6		1.0	1.0			1.0	1.0	1.0				1.0	1.0	1.0	0.9			0.8	1.0	0.8			0.9	0.8	0.9			1.0	0.5	1.0	0.8	0.6	0.5	0.4		0.8		1.0		0.8		0.3	0.9
			0.7	0.8	0.9	0.8	0.8		0.7	0.9			0.9	0.9	0.9				0.7	0.9	0.7	0.7			0.9	0.7	0.7			0.7	0.8	0.7			0.8	0.8	0.8	1.0	0.7	1.0	1.0		0.7		0.8		1.0		0.7	0.7
Axillary freckling	1.0	1.0		1.0	1.0	1.0	1.0		0.0		1.0	1.0		1.0	1.0	1.0	1.0				1.0	0.4			0.3	1.0	0.6	0.6	0.4	1.0	0.4			1.0	1.0		1.0		1.0	0.0	0.9			0.6	1.0	0.6			0.3	0.6
	0.8	1.0		1.0	0.8	0.7	0.7		1.0		1.0	0.9		1.0	1.0	1.0	0.9				0.8	0.7			0.9	0.8	0.7	0.8	0.7	0.7	0.7			1.0	0.8		1.0		1.0	1.0	0.7			0.7	1.0	0.8			0.8	0.8
Embryonal rhabdomyosarcoma	0.6	0.6	0.6	0.3	0.6	0.6	1.0	0.6	0.4	0.6	1.0	0.8		0.6	0.3	0.6	0.6		1.0	1.0	0.5		0.5	0.5	0.9		1.0				1.0			0.6		0.4	0.6	0.6	0.6	0.4	0.6	0.6	1.0		0.5	0.9	0.7			0.5
	0.8	0.7	0.7	0.8	0.8	0.8	0.8	0.8	1.0	0.8	0.8	0.7		0.8	0.9	0.8	0.8		0.9	0.9	0.8		0.8	0.8	0.7		0.9				0.9			0.8		0.8	0.7	0.8	0.9	0.8	0.8	0.7	0.9		0.8	0.7	0.9			0.7
Inguinal freckling	1.0	1.0		1.0	1.0	1.0	1.0				1.0	1.0		1.0	1.0	1.0	1.0					1.0	1.0	1.0		1.0	0.3				0.3			1.0	1.0		1.0		1.0	0.0	0.3				1.0				0.3	0.6
	0.7	0.9		0.9	0.8	0.8	0.8				0.8	0.9		1.0	0.8	0.9	0.9					0.7	0.8	0.8		0.8	0.7				0.8			0.9	0.8		0.8		0.9	0.7	0.7				0.8				0.8	0.7
Meningioma	0.9		0.8			0.7	0.4	0.9	0.0	0.8	0.9	0.9	0.8			0.9	0.6		0.9	0.6		0.9	0.7		0.6		0.4	0.8			0.7		0.2			0.2		0.7	0.6	0.7	0.6	0.7	0.4	0.8	0.8	0.7	0.6			0.9
	0.7		0.7			0.7	1.0	0.8	0.8	0.7	0.8	0.8	0.8			0.8	0.8		0.7	0.8		0.9	0.9		0.8		0.8	0.8			0.7		0.7			0.8		1.0	0.8	0.8	0.8	0.9	0.8	0.8	0.7	0.8	0.8			0.8
Scoliosis	0.9	0.9	0.9	0.9	0.2	0.9	0.4	0.9	0.2		0.2	0.9	0.9	0.0	0.9	0.9	0.2	0.0	0.2	0.2		0.9	0.9		0.4	0.2	0.6	0.7		0.9	0.2	0.0	0.9	0.9				0.2	0.9	0.4	0.4	0.0	0.9			0.2	0.0	0.9	0.0	0.4
	0.8	0.8	0.8	0.7	0.8	0.8	0.7	0.8	0.8		0.8	0.8	0.7	0.8	0.8	0.7	0.7	1.0	0.8	0.8		0.8	0.8		0.7	0.7	0.8	0.8		0.7	0.8	1.0	0.8	0.7				0.8	0.8	1.0	0.8	0.8	0.7			0.7	0.7	0.7	0.7	0.9
Cerebellar glioma	0.8	0.8			0.8		0.8	0.8		0.5	0.7	0.8		0.8		0.8	0.8		0.8	0.8					0.8	0.8	0.0				0.5		0.8	0.8	0.7		0.8	0.7	0.7	0.3	0.5	0.8	0.5				0.8	0.8		
	0.7	0.7			0.8		0.7	1.0		0.9	0.7	0.8		0.8		0.9	0.7		0.7	0.8					0.8	0.7	0.7				0.8		0.7	0.7	0.8		0.8	0.7	0.8	1.0	0.8	0.7	0.7				0.8	0.7		
Intellectual disability, mild	1.0			0.4			0.8		1.0	0.7	1.0					0.4	0.4	1.0						1.0			0.9			0.7	0.0	0.9	1.0			1.0		0.2		0.6	0.2	0.7		1.0		1.0		1.0		
	0.8			0.7			0.8		0.8	1.0	0.8					0.7	0.8	1.0						0.8			1.0			0.7	0.7	0.8	1.0			0.8		1.0		0.8	0.8	1.0		0.7		1.0		0.8		
Pilocytic astrocytoma	0.7	0.7			0.8	0.9	0.7	0.6			0.9	0.8		0.7		0.8	0.8		0.8	0.7					0.7	0.7	0.0				0.5		0.7				0.8	0.7	0.5	0.3	0.5	0.8	0.3				0.5			
	0.7	0.7			0.8	0.8	0.8	0.8			0.8	0.8		0.8		0.8	0.7		0.7	0.8					0.8	0.7	0.7				0.7		0.7				0.8	0.8	0.7	0.8	1.0	0.8	0.7				0.8			
Kyphosis	0.8	0.8	0.8	0.8	0.2	0.8		0.8	0.2		0.2	0.8	0.2	0.0	0.9	0.8	0.2		0.2	0.2		0.8	0.8		0.0		0.5			0.8	0.2		0.8	0.8				0.2	0.8	0.4	0.4		0.5			0.0	0.0	0.8	0.0	
	0.8	0.8	0.7	0.7	0.7	0.8		0.8	1.0		0.9	0.8	0.8	0.7	0.9	0.7	0.7		0.8	0.8		0.8	0.8		0.7		0.8			0.7	0.7		0.8	0.7				1.0	0.8	0.9	0.7		0.7			0.8	0.8	0.7	0.7	
Multiple cafe-au-lait spots				1.0	1.0		0.3		0.8	1.0			1.0	1.0	1.0					1.0	0.8					0.8	0.5				0.3				1.0		1.0	0.5		0.5	0.3				1.0				0.3	0.7
				0.8	0.8		0.7		0.9	0.8			0.8	0.8	0.8					0.8	0.7					0.7	0.7				0.7				0.8		0.7	1.0		1.0	0.7				0.7				0.8	0.7
Macrocephaly	0.4	0.5	0.9	0.9	0.3	0.9	0.3	0.5	0.5		0.2	0.4		0.3	0.5	0.4	0.9	0.3				0.4	0.4	0.4	0.5		0.5		0.8	0.4	0.2		1.0	0.4			0.3	0.5	0.4	0.5	0.5		0.4							
	0.8	0.9	0.8	1.0	0.7	0.8	0.7	0.8	0.7		0.8	0.8		0.7	0.8	0.7	0.7	0.8				0.8	0.8	0.8	0.7		0.8		0.8	0.7	0.7		1.0	0.7			0.7	0.7	0.8	0.8	0.8		0.7							
Lipoma	0.4	0.4	0.2	0.2			0.4	0.4	0.2		0.4	0.4		0.0	0.2	0.4			0.4	0.4		0.4		0.5	1.0		0.5	0.9			0.9			0.2				1.0	0.5	0.5	0.5	0.4	0.5		0.8		0.5		0.6	0.4
	0.7	0.7	0.7	0.7			0.7	1.0	1.0		0.8	0.7		0.8	0.8	0.8			0.8	0.8		0.7		0.7	0.8		0.7	0.8			0.8			0.7				1.0	0.7	0.8	0.7	0.9	0.8		0.8		1.0		0.7	0.7
Specific learning disability	0.7	0.3					0.3	0.0	0.7	0.7	1.0					1.0	0.3	0.7				0.3		1.0			0.3		0.8	1.0	0.0		0.7			0.7		0.2		0.8	0.3	0.7	0.0			0.7		1.0		
	0.9	1.0					0.8	0.8	0.8	0.8	0.9					0.7	0.8	1.0				0.8		0.9			0.7		0.7	0.8	0.9		1.0			0.8		0.7		0.9	0.7	1.0	0.8			1.0		0.9		
Seizure	0.8	0.6	0.0	0.6	0.0	1.0	0.6	0.0	0.6	0.0	0.4	0.0	0.0	0.4	0.0	0.7	0.6	0.4				0.5		0.4	0.0		0.4		0.4		0.4	0.0	0.7			0.6		0.3		0.7	0.5	0.6	0.4		0.0	0.4	0.0	0.4	0.7	
	0.8	1.0	0.7	1.0	0.8	0.7	1.0	0.7	0.7	0.7	0.8	0.7	0.9	0.8	0.8	0.7	1.0	1.0				0.7		0.8	0.8		0.7		0.7		0.8	1.0	0.8			0.7		0.8		0.8	0.7	0.9	0.7		0.7	1.0	0.8	0.8	0.7	
Tibial pseudarthrosis	1.0	1.0	1.0	1.0		0.2		0.2		1.0					0.4		1.0				0.6						0.2			1.0	0.1			1.0					0.6	0.3	0.2							1.0		
	0.8	0.7	1.0	0.7		0.8		0.7		0.9					0.8		0.9				0.7						0.7			0.8	0.9			1.0					0.7	0.8	1.0							0.8		
Hypertelorism	0.3		0.3		0.3	0.3	0.3	0.3	1.0		0.3	0.3		0.3	0.3	0.3	0.3		0.3				0.3	0.3		0.3	0.8				0.0				0.3		0.3		0.3	1.0	0.3	0.4		0.3	0.3	0.4				0.3
	0.7		0.8		0.8	0.7	0.8	0.8	1.0		0.7	0.7		0.8	0.8	0.8	0.8		0.7				0.8	0.7		0.7	1.0				0.8				0.8		0.8		0.7	1.0	0.7	1.0		0.7	0.7	0.7				0.7
Hypopigmented macule		0.4			0.6		0.5		0.0			0.3		0.4				0.9	0.3			0.5			0.3		0.6	0.7	0.6		0.5					0.5		0.6		0.6	0.5				0.4	0.7	0.3		0.4	
		0.7			0.7		0.8		0.7			0.7		0.7				1.0	0.8			0.7			0.8		0.7	0.8	0.7		0.7					0.9		0.8		0.8	0.7				0.8	0.8	0.7		0.7	
Downslanted palpebral fissures		0.6	0.6	0.3	0.5		0.5	0.5	0.3		0.3	0.3		0.6	0.3	0.3	0.6	0.3	0.5				0.3		0.3		0.0				0.3			0.3			0.6	0.4	0.3	0.4	0.3				0.3				0.6	
		0.8	0.7	0.7	1.0		0.8	0.8	0.7		0.7	0.7		0.7	1.0	0.7	0.7	1.0	0.7				0.7		0.8		0.7				1.0			0.7			0.7	0.8	0.7	0.8	0.9				0.7				0.8	
Breast carcinoma	0.4						0.4	0.4	0.0		0.4	0.4				0.4			0.4	1.0				1.0	0.7		0.6				0.4							0.4		0.4	0.9	0.4	1.0				1.0			
	0.7						0.7	0.9	1.0		0.8	0.7				0.8			0.8	0.7				0.7	0.8		1.0				0.7							0.9		0.7	0.8	0.8	0.7				1.0			
Medullary thyroid carcinoma	0.4					0.6	0.4	0.3	0.0	0.4	0.6	0.6	0.6	0.6		0.3			0.6	0.6			0.6		0.4		0.3				0.4							0.6		0.4	1.0		0.4				0.4			
	0.8					0.8	0.9	0.8	0.8	0.8	0.7	0.8	1.0	0.8		0.8			1.0	1.0			1.0		0.8		0.9				0.9							0.8		0.8	0.8		0.8				1.0			
Colon cancer								0.6	0.0	0.3	0.3	0.3			0.5			0.9	0.6	0.6					0.9		0.0				0.6							0.4		0.5	0.4		0.6	0.4	0.6		1.0		0.3	
								0.7	0.7	0.8	0.8	0.8			0.8			0.7	1.0	0.7					0.8		1.0				0.7							0.8		0.7	1.0		0.8	0.7	0.8		0.9		0.9	
Ventricular septal defect	0.4	0.0	0.0		0.9		0.0	0.7	0.7		0.5	0.0		0.0	0.0			0.0	0.5	0.5					0.0		0.5		0.7		0.4		0.5			1.0		0.0		0.5	0.0	0.7	0.5			0.5	0.0		0.0	
	0.8	0.7	1.0		0.8		0.8	0.7	0.8		0.8	0.7		0.8	0.8			1.0	0.8	0.8					0.8		0.8		0.8		0.8		0.8			0.8		0.7		0.8	0.7	0.9	0.8			0.9	0.7		0.7	
Hydrocephalus	0.2		0.3	0.4				0.0	0.0			0.3	0.4			0.3			0.4	0.3				0.6	0.0		0.0	0.4	0.9		0.4		0.9			0.8		0.7		0.5	0.4	0.2	0.0			0.4		0.2	0.0	
	0.8		0.7	0.9				0.7	0.7			0.8	0.9			0.8			0.7	0.7				0.8	0.7		0.8	0.8	0.8		0.8		0.9			0.7		0.8		0.9	0.8	0.9	0.8			0.7		1.0	0.7	
Spina bifida	0.2		0.7					0.7	0.0							0.2								0.2			0.0	0.7	0.3		0.3		0.2			0.3		0.6	1.0	0.9	0.3	0.6	0.7	0.8						
	0.8		0.9					0.7	0.7							0.8								0.8			0.8	0.7	0.7		0.9		0.8			0.8		0.8	0.7	0.9	0.9	0.7	0.9	0.7						
Coarctation of aorta	0.5	0.7						0.5	0.5					0.7									0.5		0.0		0.0	0.7	0.4		0.7		0.6					0.7		0.4	0.4		0.5			0.5			0.4	
	0.7	0.8						0.8	0.9					0.7									0.7		0.8		0.7	0.8	0.9		0.7		0.8					0.8		1.0	0.7		0.7			0.7			0.8	
Short stature	0.8	0.9	0.9	0.0	0.0		0.0	0.0	1.0		0.0	0.0	0.8	0.0	0.0	0.0		0.8							0.0		0.0				0.0	0.9	0.0		0.8			0.0		0.0	0.7	0.0	0.0	0.8		0.0	0.0		0.0	
	0.9	1.0	1.0	0.7	0.7		0.7	1.0	0.8		0.9	0.8	1.0	0.8	0.8	0.7		1.0							0.8		1.0				0.8	1.0	1.0		1.0			0.8		0.7	1.0	0.8	0.8	0.7		0.8	0.8		0.8	
Renal artery stenosis	1.0	0.2		0.2	0.2				0.3		0.2	0.2		0.4	0.2	0.2									0.2	0.2	0.0		0.2		0.4			0.2	0.2		0.2	0.5	0.2	0.4	0.2		0.4		0.4	0.4	0.2		0.3	
	1.0	0.7		0.7	0.8				0.9		0.7	0.7		0.7	0.7	0.7									0.8	0.8	0.9		0.7		0.8			0.7	0.8		0.7	0.8	0.7	0.9	0.8		0.8		0.8	0.7	1.0		0.7	
Hypertension	0.0	0.6	0.0	0.9	0.0			0.7	0.5					0.9	0.0										0.0		0.0	0.6	0.5		0.3	0.4	0.0			0.5		0.0		0.7	0.4		0.4		0.4	0.6			0.0	
	0.8	0.8	0.8	0.7	0.9			0.7	0.8					0.8	0.7										0.7		0.8	0.8	0.9		0.9	0.7	1.0			0.8		1.0		0.7	0.7		0.8		1.0	0.8			0.8	

Table S18: Ontological and textual similarity scores for the top 50 articles annotated by stEngine bi-encoder ranked for NF1

Phenotypes	29290338	26178382	25061003	19706204	38680880	35096710	34199217	34352903	17636453	38533410	32014052	35069816	23793209	33919865	27933191	35892827	36831560	28573359	34322401	26110128	34712008	37496887	25276129	30190611	34544639	35885913	34159396	34351021	32393377	37073110	34202860	32147744	36803953	29618358	34168676	31703719	35200700	37239385	35382488	36091172	29552546	19032739	24767283	37191267	31752929	29311971	34405046	36339399	37576110	30158741
Lisch nodules	1.0	1.0	0.8	1.0	1.0	1.0	1.0	0.8	1.0	1.0	1.0	1.0	1.0	1.0	0.6	1.0	1.0	0.5	0.8	1.0	0.8	0.7	1.0	1.0	1.0	1.0	1.0	0.6	1.0	1.0	0.6	1.0	1.0	0.9	1.0	1.0	0.6	1.0	1.0	0.5	0.6	0.7	1.0	0.7	1.0	1.0	0.4	0.4	0.5	0.8
	1.0	1.0	0.8	1.0	1.0	1.0	1.0	0.8	1.0	0.9	1.0	0.9	0.9	1.0	0.8	1.0	0.9	0.7	0.7	1.0	0.8	0.7	1.0	1.0	0.8	1.0	0.9	0.7	1.0	1.0	1.0	0.8	0.8	0.7	1.0	0.8	1.0	1.0	0.8	1.0	0.9	0.9	0.8	0.8	0.8	0.8	1.0	0.8	0.8	0.8
Optic nerve glioma	1.0	1.0	0.8	0.8	1.0	1.0	1.0	1.0	1.0	1.0	1.0	1.0	1.0	1.0	0.4	1.0	1.0	0.8	0.5	0.7	1.0	0.8	1.0	1.0	0.6	1.0	1.0	0.8	1.0	1.0	0.8	1.0	1.0	1.0	1.0	1.0	0.3	1.0	1.0	0.7	1.0	0.8	1.0	0.8	0.3	0.5	0.8	0.8	0.2	1.0
	0.9	1.0	1.0	1.0	0.9	0.8	0.8	0.9	0.9	1.0	0.9	1.0	1.0	0.8	1.0	0.8	0.8	0.9	0.9	0.8	0.7	0.8	1.0	0.8	0.7	1.0	1.0	0.7	1.0	1.0	1.0	0.8	1.0	0.8	0.8	1.0	0.9	0.9	1.0	0.9	1.0	0.8	1.0	1.0	0.9	0.7	0.8	0.7	0.9	0.8
Intellectual disability, mild	1.0	1.0	0.9	1.0	0.8	1.0	1.0	1.0	1.0	1.0	0.7	1.0	1.0	1.0	1.0	1.0	1.0	0.4	1.0	1.0	0.8	0.7	1.0	1.0	0.4	1.0	0.4	1.0	1.0	1.0	0.6	1.0	0.8	0.7	0.7	1.0	1.0	1.0	0.4	0.4	1.0	0.4	0.7	1.0	1.0	1.0	0.3	1.0	0.8	0.7
	1.0	0.8	0.8	0.8	0.8	0.8	0.8	0.8	1.0	1.0	0.8	1.0	1.0	0.8	1.0	1.0	0.8	0.8	0.8	1.0	1.0	0.7	1.0	0.8	1.0	0.8	0.8	1.0	0.8	1.0	0.7	0.9	0.7	0.8	1.0	0.8	1.0	1.0	0.7	1.0	1.0	0.7	0.8	1.0	1.0	1.0	0.8	0.8	0.8	1.0
Short stature	1.0	0.9	0.7	0.9	0.9	0.9	1.0	0.8	0.8	0.9	0.8	1.0	0.8	1.0	0.8	0.9	0.9	0.9	0.9	0.8	0.8	0.8	1.0	1.0	0.9	1.0	1.0	0.8	1.0	1.0	0.8	0.8	0.6	0.8	0.7	0.7	0.8	0.8	1.0	0.8	0.8	0.0	1.0	0.7	1.0	0.9	0.9	0.8	0.8	0.8
	1.0	0.8	0.8	0.8	0.7	0.8	0.7	1.0	0.8	0.7	0.9	0.8	0.8	1.0	0.8	0.9	0.8	0.7	0.7	0.8	1.0	0.7	1.0	1.0	0.7	1.0	1.0	1.0	1.0	1.0	0.9	0.8	0.8	0.7	0.8	0.8	1.0	0.8	1.0	0.8	0.7	0.7	1.0	0.8	0.8	0.7	0.7	0.8	0.8	0.7
Spinal neurofibroma	1.0	1.0	1.0	1.0	0.8	0.8	1.0	0.8	1.0	0.8	1.0	0.8	1.0	1.0	0.7	0.8	1.0	0.7	0.7	0.8	0.7	0.8	0.8	1.0	1.0	1.0	0.8	0.8	0.8	0.8	0.6	1.0	0.8	0.8	1.0	1.0	0.8	0.8	1.0	0.8	1.0	0.8	0.9	0.6	0.8	1.0	0.8	0.8	0.7	1.0
	0.8	0.8	0.8	0.8	0.8	0.7	0.8	0.8	0.8	0.9	0.8	0.8	0.7	0.9	0.7	0.8	0.7	0.8	1.0	0.8	1.0	0.7	0.8	0.8	0.8	0.7	0.8	0.9	0.8	0.8	0.8	0.8	0.8	0.8	0.8	0.7	0.7	0.8	0.7	0.7	0.7	0.8	0.8	0.8	0.7	0.8	0.7	0.8	0.7	1.0
Few cafe-au-lait spots	0.8	0.9	0.8	1.0	1.0	1.0	0.9	0.7	1.0	1.0	0.9	1.0	1.0	0.6	1.0	1.0	0.9	0.8	0.9	1.0	0.7	0.8	0.3	0.6	0.9	1.0	0.9	0.8	0.9	1.0	0.3	1.0	1.0	1.0	1.0	1.0	0.4	0.7	0.7	0.9	1.0	0.8	0.9	0.8	0.7	1.0	1.0	0.8	0.3	0.7
	0.9	0.8	1.0	0.9	0.7	0.9	0.8	0.8	0.9	0.7	0.7	0.8	0.9	0.8	0.9	0.8	0.8	1.0	0.8	0.8	0.9	0.7	1.0	0.7	0.7	0.8	0.7	0.7	0.8	0.8	0.8	0.7	1.0	0.8	0.7	0.8	0.8	0.7	0.9	0.8	0.9	1.0	0.9	1.0	0.7	0.9	0.9	0.8	1.0	0.9
Neurofibrosarcoma	1.0	1.0	0.6	1.0	1.0	1.0	1.0	1.0	1.0	1.0	1.0	1.0	1.0	1.0	0.3	0.6	1.0	1.0	0.6	1.0	0.6	0.6	1.0	1.0	0.5	1.0	1.0	0.4	1.0	1.0	0.6	1.0	1.0	1.0	1.0	1.0	0.3	1.0	1.0	0.4	1.0	1.0	1.0	0.6	1.0	1.0	0.0	0.3	0.0	1.0
	0.9	0.9	0.7	0.9	0.9	0.8	0.9	0.9	1.0	0.9	0.9	1.0	0.9	0.8	0.8	0.8	0.8	0.8	0.7	0.9	0.8	0.8	0.9	0.9	0.7	0.8	0.8	0.7	0.9	0.9	0.9	0.8	0.8	0.8	0.9	0.8	0.7	0.7	0.8	1.0	0.9	0.9	0.7	1.0	0.8	0.7	0.7	0.8	0.7	0.9
Specific learning disability	1.0	0.9	0.3	0.7	0.7	0.8	1.0	0.7	1.0	0.9	0.9	1.0	0.7	1.0	0.8	1.0	1.0	0.8	0.7	0.8	0.7	0.7	0.8	1.0	0.3	1.0	0.3	0.9	1.0	1.0	0.7	1.0	1.0	1.0	1.0	0.7	0.7	0.7	0.7	0.7	0.8	0.8	1.0	0.7	1.0	0.7	0.8	1.0	0.7	0.9
	0.8	0.8	0.7	1.0	1.0	0.7	0.9	0.8	0.7	0.7	0.8	0.9	1.0	0.9	0.8	0.8	0.9	0.7	0.9	0.9	1.0	0.7	0.8	0.9	0.9	0.9	0.7	1.0	0.9	0.8	0.7	0.8	0.9	0.8	0.7	0.9	0.7	1.0	0.7	0.8	0.8	0.7	0.8	1.0	0.7	0.8	0.8	0.7	0.9	0.8
Plexiform neurofibroma	1.0	1.0	0.7	0.7	1.0	1.0	1.0	1.0	1.0	1.0	1.0	1.0	1.0	0.7	0.6	0.6	0.7	0.6	0.6	1.0	0.7	0.7	0.7	1.0	0.7	1.0	1.0	0.7	1.0	1.0	0.6	1.0	1.0	1.0	1.0	0.7	0.6	1.0	1.0	0.6	0.7	0.7	1.0	0.6	0.7	1.0	0.4	0.5	0.3	0.7
	1.0	0.9	0.8	0.8	0.9	0.9	0.8	1.0	1.0	0.9	0.9	0.8	1.0	0.8	0.8	0.8	0.8	0.7	0.7	1.0	0.8	0.8	0.8	0.9	0.9	1.0	1.0	0.8	1.0	0.8	0.8	0.9	0.8	0.9	0.8	0.9	0.7	0.9	0.8	0.7	0.8	1.0	0.8	0.8	0.8	0.7	0.7	0.9	0.9	0.7
Pheochromocytoma	0.6	0.9	0.9	0.9	0.9	0.6	0.6	0.8	0.9	0.9	0.9	0.9	0.9	0.9	0.8	0.6	0.9	0.9	0.9	0.9	0.9	0.9	0.9	0.9	0.9	0.9	0.8	0.8	0.6	0.7	0.9	0.9	0.9	0.6	0.7	0.6	0.5	0.9	0.6	0.9	0.7	0.7	0.6	0.8	0.6	0.7	0.8	0.6	0.4	0.8
	0.8	0.9	1.0	1.0	0.9	0.8	0.8	0.7	1.0	0.7	1.0	0.7	0.7	0.8	0.7	0.9	0.7	0.8	0.9	1.0	0.9	0.8	1.0	1.0	1.0	1.0	0.7	1.0	0.8	0.7	1.0	0.8	0.9	0.7	0.7	0.8	0.8	1.0	0.9	1.0	0.8	0.7	0.8	0.7	0.7	0.8	1.0	0.7	0.8	0.7
Axillary freckling	1.0	1.0	0.6	1.0	1.0	1.0	1.0	0.6	1.0	1.0	1.0	1.0	1.0	1.0	0.6	1.0	1.0	0.4	0.6	0.6	0.6	0.6	1.0	1.0	0.6	1.0	1.0	0.3	1.0	1.0	0.3	1.0	1.0	1.0	1.0	1.0	0.6	1.0	1.0	1.0	1.0	0.6	1.0	0.4	0.3	0.7	0.6	0.6	0.4	0.6
	1.0	0.8	0.8	1.0	1.0	0.9	0.9	0.7	0.8	0.8	1.0	1.0	1.0	0.8	1.0	0.8	0.8	0.7	0.8	0.7	0.7	0.8	1.0	0.8	0.8	1.0	0.9	0.7	1.0	1.0	0.9	1.0	0.9	0.7	0.9	0.8	0.7	0.8	1.0	0.7	0.8	0.8	0.9	0.8	0.8	0.7	0.7	0.8	1.0	0.8
Scoliosis	0.9	0.9	0.9	0.9	0.9	0.9	0.9	0.9	0.9	0.9	0.9	0.9	0.9	0.9	0.9	0.7	0.9	0.9	0.9	0.5	0.5	0.5	0.9	0.9	0.5	0.9	0.9	0.5	0.9	0.9	0.9	0.4	0.9	0.9	0.9	0.9	0.9	0.5	0.9	0.9	0.9	0.9	0.6	0.5	0.4	0.9	0.9	0.9	0.9	0.5
	0.8	0.7	0.7	0.8	0.8	0.8	0.8	0.8	0.8	0.8	0.7	0.8	0.8	0.8	0.8	0.9	0.7	0.7	0.8	1.0	0.7	0.7	0.8	0.8	0.7	0.8	0.8	0.7	0.8	0.8	0.7	1.0	0.7	0.7	0.8	0.8	0.8	0.9	0.8	0.8	0.8	0.7	0.7	0.8	0.8	0.8	0.7	0.7	0.8	0.8
Macrocephaly	1.0	0.9	1.0	0.9	0.9	0.9	0.9	0.9	0.9	0.9	0.5	0.9	0.9	0.9	0.9	0.5	0.9	0.5	0.9	1.0	0.8	0.5	0.8	0.9	0.5	0.4	0.5	0.9	0.9	0.9	0.9	0.9	0.5	0.5	0.9	0.5	0.9	0.9	0.9	0.9	0.5	0.9	0.9	0.5	0.9	0.9	0.8	0.4	0.9	1.0
	0.8	0.7	0.9	1.0	0.8	1.0	0.7	1.0	1.0	0.8	0.8	1.0	0.8	0.8	0.8	0.9	1.0	0.7	0.8	1.0	0.7	0.8	0.7	1.0	1.0	0.8	0.7	0.7	1.0	1.0	0.9	1.0	0.7	0.8	0.7	0.7	0.9	1.0	1.0	0.9	0.8	0.8	1.0	0.7	0.8	0.9	0.7	0.8	0.8	0.8
Embryonal rhabdomyosarcoma	1.0	0.6	0.7	1.0	1.0	1.0	1.0	1.0	1.0	0.7	0.6	0.6	0.8	0.9	1.0	1.0	0.6	0.7	0.7	0.9	1.0	1.0	0.8	1.0	0.8	0.6	1.0	0.8	0.6	0.6	1.0	0.6	1.0	0.6	0.6	0.6	0.4	1.0	0.8	0.7	1.0	1.0	0.6	1.0	0.4	0.6	0.4	0.6	0.4	0.9
	0.8	0.8	0.9	0.9	0.8	0.9	0.8	0.9	0.7	0.7	0.9	0.8	0.7	0.7	0.7	1.0	0.8	0.9	0.9	0.7	0.7	0.8	0.7	0.8	0.7	0.8	0.7	0.7	0.8	0.8	0.9	0.8	0.8	0.8	0.9	0.8	0.8	0.9	0.7	1.0	0.9	0.8	1.0	0.9	0.7	0.8	0.7	0.8	0.8	0.7
Inguinal freckling	1.0	1.0	0.6	1.0	1.0	1.0	1.0	0.6	1.0	1.0	1.0	1.0	1.0	1.0	0.6	0.6	1.0	0.4	0.4	1.0	0.6	0.4	1.0	1.0	0.6	1.0	1.0	0.3	1.0	1.0	0.3	1.0	1.0	1.0	1.0	1.0	0.3	1.0	1.0	0.6	1.0	0.4	1.0	0.4	0.3	0.3	0.6	0.4	0.3	0.6
	0.9	0.8	1.0	0.8	0.9	0.8	0.9	0.7	0.8	0.8	0.8	0.8	0.9	0.8	0.9	0.8	0.8	0.7	0.8	0.7	0.9	0.7	0.9	0.9	0.9	0.9	0.9	0.7	0.9	0.9	0.8	0.8	0.8	0.8	0.8	0.8	0.7	0.8	0.9	0.8	0.7	0.7	0.9	0.8	0.9	0.8	0.8	0.7	0.8	0.8
Hypertelorism	1.0	1.0	0.4	1.0	0.4	1.0	1.0	1.0	0.4	0.8	0.8	0.4	1.0	0.3	1.0	1.0	1.0	0.4	0.8	0.4	0.8	0.8	1.0	1.0	1.0	0.3	0.4	0.4	0.3	1.0	0.4	1.0	1.0	0.8	0.4	1.0	1.0	0.3	1.0	1.0	1.0	1.0	1.0	0.8	1.0	1.0	0.8	0.8	0.8	0.4
	1.0	1.0	0.7	1.0	0.8	1.0	1.0	1.0	0.8	1.0	0.8	0.7	1.0	0.8	1.0	1.0	1.0	0.7	0.7	0.8	0.7	0.7	1.0	1.0	1.0	0.7	0.7	1.0	0.7	1.0	0.8	1.0	1.0	1.0	0.8	1.0	1.0	0.9	1.0	0.9	1.0	0.7	1.0	0.7	0.8	1.0	1.0	0.7	1.0	0.8
Kyphosis	0.8	0.8	0.9	0.8	0.9	0.8	0.8	0.6	0.9	0.8	0.8	0.9	0.9	0.8	0.9	0.6	0.8	0.8	0.8	0.9	0.5	0.5	0.9	0.6	1.0	0.9	0.8	0.5	0.9	0.8	1.0	0.4	0.8	0.9	0.8	0.9	0.8	0.4	0.8	0.9	0.8	0.5	0.4	0.5	1.0	0.8	0.8	0.6	0.9	0.5
	0.8	0.7	0.8	0.8	0.8	0.8	0.8	0.7	0.8	0.8	0.8	0.9	0.8	0.8	0.9	0.8	0.7	0.7	0.7	0.7	0.9	0.7	0.9	0.8	0.8	0.8	0.8	0.7	0.9	0.8	0.8	1.0	0.7	0.8	0.8	0.7	0.8	1.0	0.8	0.8	0.8	0.8	1.0	0.7	0.8	0.8	0.7	0.7	0.9	0.8
Meningioma	0.7	0.7	0.9	0.9	0.7	0.9	0.7	0.9	0.7	0.8	0.9	0.9	0.7	0.9	0.8	0.9	0.7	0.7	0.7	0.7	0.9	0.7	0.7	0.8	0.9	0.9	0.9	0.4	0.6	0.7	0.9	0.7	0.7	0.6	0.6	0.7	0.8	0.9	0.7	0.7	0.6	0.9	0.4	0.9	0.4	0.6	0.7	0.9	0.3	0.9
	0.8	0.7	0.8	0.8	0.7	0.8	0.7	1.0	0.8	0.8	0.7	0.8	0.8	0.8	0.7	0.8	0.9	0.8	0.8	0.9	0.7	0.7	0.7	0.8	0.8	0.9	0.8	0.8	0.8	0.7	0.8	0.9	0.8	0.7	0.7	0.8	0.7	0.8	0.8	0.8	0.7	0.8	0.7	0.8	0.7	0.8	0.8	0.8	0.9	0.8
Pilocytic astrocytoma	0.7	0.9	1.0	1.0	1.0	0.9	0.5	1.0	1.0	0.7	1.0	0.7	0.9	1.0	0.3	1.0	0.8	0.7	0.5	0.6	1.0	0.7	0.8	1.0	0.5	1.0	1.0	0.5	0.8	0.8	0.7	0.7	1.0	0.3	0.5	0.7	0.3	1.0	0.8	0.4	0.8	0.9	0.8	0.8	0.8	0.3	0.3	0.6	0.2	1.0
	0.8	0.7	0.8	0.8	0.9	0.8	0.8	0.8	0.8	0.8	0.8	0.8	0.7	1.0	0.8	1.0	0.8	0.8	0.8	0.7	0.8	0.8	0.7	0.8	0.7	0.8	0.8	0.8	0.8	0.8	0.7	0.8	0.9	0.7	0.8	0.7	0.8	0.8	0.8	0.8	0.7	0.7	0.8	0.8	0.8	0.7	0.8	0.8	0.7	0.8
Downslanted palpebral fissures	1.0	1.0	0.7	0.7	1.0	1.0	1.0	1.0	0.5	0.6	0.5	0.6	0.7	0.6	1.0	1.0	1.0	0.5	0.5	0.4	0.7	0.5	0.3	1.0	0.7	0.3	0.5	0.5	0.3	0.5	1.0	0.6	1.0	0.6	0.6	0.6	1.0	0.5	1.0	0.8	0.7	0.5	1.0	0.7	1.0	1.0	0.6	1.0	1.0	1.0
	1.0	1.0	0.9	1.0	0.8	1.0	1.0	0.7	0.7	0.7	0.7	0.8	1.0	0.7	1.0	1.0	0.8	0.8	0.7	0.8	1.0	0.8	0.7	1.0	0.7	0.8	0.8	0.7	1.0	0.8	1.0	1.0	1.0	0.7	0.7	0.7	1.0	0.8	1.0	0.9	1.0	0.7	1.0	0.7	0.8	0.9	0.9	0.8	1.0	0.7
Pulmonic stenosis	0.9	1.0	0.9	1.0	0.7	0.5	0.9	0.9	1.0	0.9	0.5	0.4	0.5	0.9	0.9	1.0	0.9	0.7	0.5	0.4	0.5	0.9	0.5	0.9	0.9	1.0	0.4	1.0	0.4	1.0	0.4	0.9	0.4	0.0	0.9	0.9	0.9	0.5	0.0	0.9	0.9	0.3	0.9	0.5	0.9	0.7	0.3	0.9	1.0	0.5
	0.9	0.8	0.9	0.9	0.8	0.7	0.9	0.7	0.7	0.8	0.7	0.8	0.8	0.9	0.8	0.7	0.7	0.8	0.8	0.8	0.8	0.8	0.8	0.8	0.9	0.8	0.8	0.7	0.7	0.7	0.7	0.7	0.7	0.7	0.9	0.9	0.9	0.7	0.7	0.7	0.9	0.7	0.9	0.8	0.8	0.8	0.7	0.8	0.9	0.8
Ventricular septal defect	0.9	1.0	0.9	0.5	0.9	1.0	0.9	0.9	0.6	0.4	0.7	0.7	0.5	0.6	0.9	0.5	0.5	0.8	0.9	0.9	0.8	0.7	0.5	0.7	0.7	0.7	0.5	0.7	0.4	0.0	0.9	0.9	0.5	0.6	0.9	0.7	1.0	0.5	0.7	0.9	0.5	1.0	0.7	0.7	0.9	0.6	1.0	0.8	0.9	0.5
	1.0	0.9	0.7	0.8	0.8	0.8	0.9	1.0	0.7	0.8	0.7	0.7	0.8	0.7	0.8	0.8	0.8	0.7	0.8	0.7	0.8	0.7	0.7	0.7	0.7	0.9	1.0	0.8	0.7	0.8	1.0	0.8	0.8	0.8	1.0	0.9	1.0	0.7	1.0	0.7	0.8	0.7	0.7	1.0	0.8	0.8	0.9	0.8	0.8	0.8
Low-set ears	1.0	1.0	0.8	0.8	1.0	0.6	1.0	0.8	0.0	0.0	0.0	0.0	0.5	0.5	1.0	1.0	1.0	0.5	0.8	0.8	0.8	0.8	0.5	1.0	0.8	0.5	0.8	0.5	0.5	1.0	1.0	1.0	1.0	0.6	0.5	0.8	1.0	0.5	1.0	0.6	0.4	0.5	1.0	0.8	1.0	1.0	1.0	0.5	1.0	0.8
	0.8	0.7	0.8	1.0	0.7	0.7	0.9	1.0	0.8	0.7	1.0	0.7	0.8	0.7	0.8	0.9	0.8	1.0	0.9	0.8	1.0	1.0	0.7	0.8	0.8	0.8	0.8	0.8	1.0	0.7	0.8	0.9	1.0	0.7	0.8	0.7	0.8	0.8	1.0	0.8	1.0	1.0	0.8	0.8	0.9	0.8	0.8	1.0	0.8	1.0
Overgrowth	0.9	0.9	0.9	1.0	1.0	1.0	0.7	0.6	0.5	0.7	0.8	0.6	0.6	0.6	0.8	0.7	0.6	0.6	0.6	0.6	0.6	0.5	0.9	0.6	0.6	0.6	0.6	0.9	0.6	0.6	0.9	0.5	0.0	0.6	1.0	1.0	0.8	0.5	0.6	0.6	0.8	0.9	0.6	0.8	0.6	0.6	0.6	0.9	0.5	0.9
	0.7	0.7	0.8	0.8	1.0	0.8	0.7	0.7	0.7	0.7	0.7	0.8	0.8	1.0	1.0	0.8	1.0	0.8	0.8	0.7	1.0	0.7	0.7	1.0	0.8	1.0	1.0	0.7	1.0	1.0	0.7	0.8	0.7	0.7	1.0	1.0	0.9	0.9	1.0	0.8	0.7	0.7	1.0	0.7	0.8	0.7	0.8	0.8	0.8	0.7
Multiple cafe-au-lait spots	0.6	0.6	0.6	1.0	0.8	1.0	0.6	0.6	1.0	0.6	0.7	1.0	1.0	0.6	1.0	1.0	0.6	0.5	0.6	1.0	0.7	0.6	0.3	0.6	0.7	1.0	0.7	0.5	0.7	1.0	0.3	0.6	1.0	1.0	0.6	1.0	0.3	0.6	0.6	0.7	1.0	0.6	0.6	0.5	0.6	1.0	1.0	0.7	0.3	0.7
	0.7	0.8	0.7	1.0	0.7	0.8	0.8	0.7	1.0	0.8	0.8	0.8	0.8	0.8	0.8	0.7	0.8	0.7	0.7	0.8	0.8	0.7	0.8	0.7	0.9	0.8	0.7	0.7	0.7	0.8	0.9	0.7	0.9	0.7	0.8	0.7	0.8	0.7	0.9	0.8	0.8	1.0	0.7	0.7	0.7	0.8	0.8	0.7	0.8	0.8
Lipoma	1.0	1.0	1.0	0.9	0.5	0.9	0.4	0.9	0.6	0.4	0.4	0.9	0.5	0.9	0.7	0.5	0.4	0.9	1.0	0.5	0.9	0.7	0.4	1.0	0.7	0.5	0.9	0.8	1.0	0.4	0.9	0.9	0.5	0.5	0.7	0.5	0.6	1.0	0.6	0.7	1.0	0.8	0.5	1.0	0.8	0.5	0.4	0.4	0.7	1.0
	0.8	0.8	0.7	0.8	0.7	1.0	0.8	0.7	0.8	0.7	0.8	0.7	0.7	0.7	0.7	0.7	0.7	0.7	0.8	0.8	0.9	1.0	0.7	0.8	1.0	0.7	1.0	0.7	0.8	0.8	0.9	0.8	0.7	0.7	0.8	0.7	0.7	0.9	0.7	0.7	0.8	0.9	0.8	0.8	0.7	0.7	0.8	0.8	0.7	0.7
Cerebellar glioma	0.8	0.8	0.8	1.0	0.8	0.7	0.5	0.8	1.0	0.8	1.0	0.8	0.8	1.0	0.3	0.8	0.8	0.8	0.5	0.7	0.8	0.7	1.0	0.8	0.5	0.8	0.8	0.5	0.8	0.8	0.7	0.8	0.8	0.8	0.5	0.8	0.3	0.8	0.8	0.5	0.8	0.7	0.8	1.0	0.3	0.5	0.7	0.6	0.2	0.8
	0.8	0.7	0.7	0.8	0.9	0.7	0.7	0.7	0.8	0.8	0.7	0.8	0.8	0.7	0.8	0.9	1.0	0.7	0.7	0.8	0.8	0.7	0.8	0.9	0.8	0.7	0.7	0.7	0.8	0.8	0.7	0.8	0.8	0.7	0.8	0.8	0.8	0.9	0.8	0.8	0.7	0.8	0.8	0.7	0.8	0.8	0.8	1.0	0.8	0.8
Hypertension	0.4	1.0	0.9	0.5	0.9	0.6	0.4	1.0	0.9	0.9	0.9	0.9	0.9	0.9	0.5	0.3	0.9	0.9	1.0	0.9	0.5	0.9	0.9	0.5	0.9	0.9	0.6	1.0	0.9	0.0	0.5	0.5	1.0	0.3	0.4	0.4	0.5	0.9	0.9	0.5	0.9	0.4	0.6	0.9	0.6	0.4	0.3	0.9	0.8	0.9
	0.9	0.7	0.8	0.7	0.8	0.8	0.8	0.8	0.9	0.7	0.7	0.7	0.7	0.8	0.8	0.7	0.8	0.7	0.8	0.8	0.7	0.7	0.8	0.7	0.8	0.7	0.8	1.0	0.9	0.7	0.7	0.8	0.8	0.7	1.0	0.8	0.8	0.9	0.7	0.7	0.8	0.8	0.7	0.7	0.7	0.8	0.8	0.7	0.8	0.7
Pectus excavatum	0.5	1.0	0.7	1.0	0.5	0.5	0.5	0.6	0.5	0.5	1.0	1.0	1.0	1.0	1.0	1.0	0.5	0.5	0.5	0.5	0.7	0.5	1.0	0.6	0.5	0.5	0.3	0.5	0.8	1.0	0.8	0.3	1.0	1.0	1.0	1.0	1.0	0.3	1.0	0.5	0.3	0.8	0.5	0.5	0.4	0.8	0.5	1.0	1.0	0.5
	0.8	0.8	0.8	0.9	0.8	0.8	0.8	0.7	0.7	0.8	0.8	0.9	0.9	0.9	0.7	0.9	0.8	0.7	0.7	0.8	0.8	0.8	0.8	0.8	0.7	0.8	0.8	0.7	0.9	0.7	0.7	0.8	0.7	0.8	0.8	0.9	0.9	1.0	0.9	0.8	0.8	0.7	0.7	0.7	0.8	0.9	0.8	0.7	0.9	1.0
Breast carcinoma	0.7	1.0	1.0	1.0	1.0	0.4	1.0	1.0	1.0	0.4	0.7	0.4	0.4	1.0	0.8	0.4	0.4	1.0	0.9	1.0	1.0	1.0	1.0	0.4	0.8	1.0	0.8	0.4	0.4	0.4	0.7	0.7	0.4	0.4	0.4	0.6	0.4	1.0	0.4	0.4	0.4	0.6	0.4	1.0	0.0	0.6	0.9	0.6	0.0	1.0
	0.8	0.7	0.7	1.0	1.0	0.8	0.7	0.7	1.0	0.8	0.8	0.8	0.7	1.0	0.8	0.8	0.7	1.0	0.7	0.9	0.8	0.7	0.8	0.8	0.8	1.0	0.8	0.8	0.8	0.7	0.7	0.7	0.8	0.8	1.0	1.0	0.8	1.0	0.7	0.8	0.7	0.8	0.8	1.0	0.7	0.9	0.7	0.8	0.7	0.8
Renal artery stenosis	0.4	1.0	1.0	0.8	1.0	0.4	0.4	0.8	0.5	1.0	1.0	1.0	0.4	1.0	0.8	0.5	1.0	0.8	0.8	0.4	0.8	1.0	0.4	0.4	0.8	0.5	0.4	0.8	1.0	0.2	0.8	0.3	0.4	0.5	0.8	0.3	0.8	0.8	0.3	0.5	1.0	0.8	0.4	0.8	0.4	0.4	0.4	0.8	0.4	0.8
	0.7	0.9	0.7	0.8	0.8	1.0	0.8	0.9	0.8	0.8	1.0	0.9	0.9	1.0	0.7	0.7	0.8	0.7	0.9	0.7	0.9	0.8	0.7	0.8	0.8	0.7	0.7	0.9	0.7	0.8	0.8	0.8	0.7	0.7	0.8	0.8	0.8	0.7	0.9	0.9	0.8	0.7	0.8	0.9	0.8	0.7	0.9	0.7	0.8	0.8
Hydrocephalus	0.4	0.5	0.7	0.7	0.4	0.9	0.5	0.9	0.9	1.0	0.4	0.9	0.4	0.4	0.5	0.7	0.9	0.7	0.9	0.7	1.0	1.0	0.9	0.4	0.5	0.4	0.7	0.8	0.4	0.4	0.7	0.4	0.3	0.9	0.4	0.8	0.4	0.7	0.5	0.9	0.4	0.9	0.4	1.0	0.9	0.5	0.9	0.4	0.8	0.7
	0.8	0.7	0.7	1.0	0.7	0.9	0.7	0.9	0.8	0.7	0.7	0.7	0.9	0.8	0.8	0.7	0.8	0.8	0.8	0.7	0.8	0.8	0.9	0.9	0.8	0.8	1.0	0.9	0.9	0.9	0.8	0.9	0.8	0.7	0.7	0.7	0.9	0.8	0.7	0.8	0.8	1.0	0.9	0.8	0.9	0.8	0.8	0.7	0.7	0.7
Colon cancer	1.0	0.4	1.0	1.0	0.6	0.7	0.6	0.6	0.6	0.6	0.6	0.4	0.6	0.6	0.6	1.0	0.6	1.0	0.6	0.9	1.0	1.0	0.6	0.6	0.9	0.6	1.0	1.0	0.4	0.6	0.6	1.0	0.6	0.4	0.6	0.3	0.6	1.0	0.6	0.7	0.6	0.6	0.0	1.0	0.4	0.6	0.6	0.5	0.0	0.9
	0.8	0.7	1.0	1.0	0.7	0.8	0.7	0.8	0.9	0.8	0.7	0.7	0.8	0.9	0.7	0.8	0.7	1.0	0.8	0.8	0.7	1.0	0.8	0.9	0.9	0.8	0.7	0.9	0.8	0.8	0.8	0.7	0.8	0.7	0.7	0.8	0.7	0.7	0.7	0.7	0.8	0.8	0.7	1.0	0.8	0.7	0.8	0.7	0.7	0.8
Coarctation of aorta	0.5	0.5	0.9	0.6	0.9	0.7	0.7	0.7	0.9	0.9	0.9	0.9	0.7	0.5	0.9	0.6	0.9	0.9	0.9	0.4	0.7	0.7	0.5	0.6	1.0	0.9	0.5	0.5	0.9	0.0	0.9	0.5	0.0	0.5	0.6	0.3	0.9	0.6	0.0	1.0	0.5	0.5	0.5	0.5	0.5	0.5	0.9	0.9	0.9	0.7
	0.9	0.7	0.9	0.8	0.9	0.8	0.8	0.8	0.7	0.9	0.8	0.7	0.8	0.9	0.8	0.7	0.9	0.7	0.9	0.7	0.7	0.8	0.7	0.8	0.7	0.8	0.7	0.7	0.8	0.9	0.9	0.7	0.7	0.7	0.7	0.7	0.8	0.8	0.8	0.8	0.9	0.8	0.9	0.8	0.9	0.8	0.9	0.8	0.8	0.8
Hypertrophic cardiomyopathy	0.9	0.9	0.9	0.4	0.9	0.8	0.9	0.7	0.9	0.9	0.4	0.3	0.0	0.4	0.9	0.5	0.4	1.0	1.0	0.7	0.9	0.9	0.8	0.4	0.9	0.4	0.4	0.9	0.3	0.0	0.5	0.9	0.0	0.3	0.6	0.0	0.9	0.3	0.5	0.5	0.9	0.7	0.9	1.0	0.9	0.5	0.5	0.8	1.0	0.8
	0.7	1.0	0.8	0.9	1.0	0.8	0.9	0.8	0.7	0.7	0.7	0.8	0.7	0.8	1.0	0.8	0.8	0.8	0.8	0.8	0.7	0.7	0.7	0.8	0.8	0.8	0.8	0.7	0.7	0.8	0.8	0.7	0.7	0.7	0.8	0.8	0.9	0.8	0.8	0.7	0.8	0.7	0.7	1.0	1.0	0.8	0.8	0.7	0.7	0.8

Table S19: Ontological and textual similarity scores for the top 50 abstracts annotated by stEngine bi-encoder ranked for Noonan syndrome

Phenotypes	12940461	1200027	34636477	12558112	18505544	7151837	8779319	561886	26852509	29061174	32467733	21416592	26043504	7450776	20503305	2264484	3177454	27192892	34481514	7506965	17716964	15770132	32949109	14564158	17100198	24826207	12101870	30092813	34999713	12364941	32006683	26518681	7943044	14564163	20814946	3734522	3276203	23565479	7124793	34956087	18348260	21857505	3856414	19842870	25076975	11883867	10665663	1887854	9783713	25230004
Low-set ears	1.0	1.0	0.6	0.0	0.4	1.0	0.8	1.0	1.0	1.0	1.0	0.6		0.5	1.0	1.0	0.9	1.0	0.8	0.6	0.0	0.5	0.6	1.0	1.0	1.0	1.0	1.0	0.6	1.0	0.6		0.5	1.0	1.0	1.0	0.7	1.0	1.0	1.0		0.6	0.5	1.0	1.0	1.0	0.9		1.0	0.6
	0.8	0.8	0.7	0.8	0.7	0.8	0.7	0.8	0.9	1.0	0.8	0.8		0.8	0.9	0.8	0.9	0.8	0.7	0.7	0.7	0.9	0.7	0.8	0.8	0.8	1.0	1.0	0.9	0.8	0.8		0.8	0.7	0.7	0.7	0.9	0.7	0.8	0.8		0.8	0.9	0.9	0.8	0.9	0.9		0.7	0.7
Micrognathia	0.9	0.9	0.9	0.9	0.6	0.9	0.9	0.9	0.9	0.9	0.9	0.7	0.4	1.0	1.0	0.4	0.7	0.9	0.7	0.9	0.5	0.5	0.9	0.9	0.9	0.5	0.9	0.3	0.5	0.9	0.7	0.4	0.6	0.4	0.9	0.9	0.5	0.3	0.4	1.0	0.5	0.9	0.3	0.9	0.9	0.3	0.4	0.3	0.9	0.9
	0.8	0.8	0.8	0.9	0.7	0.8	0.8	0.9	0.8	1.0	0.8	1.0	1.0	0.8	0.8	0.7	0.8	0.9	0.7	0.8	1.0	1.0	0.8	0.8	0.8	0.8	0.8	0.8	0.7	0.8	0.8	0.8	0.8	0.7	0.8	0.7	0.8	0.7	0.7	0.9	1.0	0.8	0.9	0.8	0.8	0.8	1.0	0.8	1.0	0.8
Downslanted palpebral fissures		1.0	1.0	0.3	0.0	1.0	0.8	0.5	0.5	1.0	1.0	1.0		1.0	1.0		0.3	0.3	1.0	0.8	1.0	1.0	1.0	1.0	1.0	0.3	0.7	1.0	0.9	1.0	1.0		0.8		0.5	1.0	0.8	0.3	1.0			0.7	1.0	1.0	0.3	0.6	0.8	1.0	1.0	1.0
		1.0	1.0	1.0	0.8	0.8	1.0	0.9	0.9	1.0	1.0	0.9		0.9	1.0		0.8	0.8	0.9	0.9	0.9	1.0	0.8	1.0	1.0	0.9	1.0	0.8	0.7	1.0	1.0		1.0		0.8	0.9	0.8	0.8	1.0			1.0	0.7	0.9	0.8	0.8	0.9	1.0	0.9	1.0
Intellectual disability	0.9	0.4	0.5	0.8	0.9	0.7	0.3	0.9	0.8	0.8	0.9	0.7	0.0	0.9	0.4	0.4	0.8	0.8	0.9	0.8	0.8	0.8	0.4	0.7	0.9	0.9	0.2		0.0	0.8	0.0	0.8	0.9	0.3	0.7	0.9	0.3	0.9	0.3	0.9	0.8	0.8	0.9	0.8	0.9	0.0		0.9	0.8	0.9
	1.0	0.7	0.8	0.9	0.8	0.8	0.8	1.0	0.8	0.7	0.7	0.7	0.9	0.8	0.7	0.7	0.8	0.9	0.7	1.0	0.7	0.9	0.7	0.8	0.7	0.7	0.8		0.9	0.9	0.9	0.7	0.7	0.8	0.9	0.8	1.0	1.0	0.8	1.0	1.0	0.9	0.8	0.8	0.9	0.8		1.0	1.0	0.8
High, narrow palate	1.0	0.4	0.3	1.0		1.0	0.4	0.3	0.3	1.0	1.0	1.0		1.0	1.0	1.0	1.0	1.0	1.0		0.3		1.0	0.4	0.4	1.0	1.0	1.0	1.0	1.0	1.0		0.4	1.0	0.0	1.0	0.5	0.0	1.0	1.0		1.0	0.6	1.0	0.4	0.4			0.4	0.6
	1.0	1.0	0.8	1.0		0.9	1.0	0.8	0.8	0.8	1.0	0.9		1.0	0.9	1.0	1.0	1.0	1.0		1.0		1.0	0.9	0.7	0.8	1.0	1.0	1.0	1.0	1.0		0.8	0.8	0.9	1.0	0.8	0.7	1.0	1.0		0.9	0.7	1.0	0.9	1.0			1.0	1.0
Hypertelorism	0.3	1.0	0.3	1.0	1.0	0.3	0.4	1.0	1.0	0.3	1.0	0.8		1.0	1.0	1.0	0.0	0.8	0.0	0.3	1.0	1.0	1.0	1.0	1.0	0.3	1.0		0.8	1.0	0.3		1.0	0.8	1.0		0.3	0.3	1.0	0.0		1.0	1.0	0.3	0.3			1.0	1.0	0.3
	0.7	1.0	0.7	0.8	1.0	0.7	1.0	1.0	1.0	0.9	1.0	0.8		1.0	1.0	1.0	0.7	1.0	0.8	0.7	1.0	1.0	1.0	1.0	1.0	0.7	0.8		0.8	1.0	0.9		0.8	1.0	1.0		0.8	1.0	0.8	1.0		0.8	0.8	0.7	0.7			1.0	1.0	0.7
Epicanthus	1.0	0.6	0.9	0.3	0.0	0.9	1.0	0.9	0.9	0.6	0.6	0.6	0.3	0.6	0.6	1.0	0.3	0.3	0.6	0.6	0.6	0.3	0.6	0.6	1.0	0.3	0.6	1.0	0.3	1.0	0.6	0.3	0.9	0.3	1.0	0.6	0.4	0.5	1.0	0.0		0.6	1.0	0.6	1.0	0.6	0.6	0.5	0.3	0.3
	1.0	0.9	0.8	0.8	0.7	0.9	1.0	0.9	0.9	0.7	1.0	1.0	0.8	0.7	0.9	0.9	0.9	0.9	1.0	0.7	1.0	0.7	0.7	0.9	0.9	1.0	1.0	1.0	1.0	1.0	1.0	0.8	1.0	0.9	1.0	1.0	0.8	0.8	1.0	0.8		1.0	1.0	0.9	1.0	0.8	0.8	0.8	1.0	0.9
Short neck		0.3	0.3	1.0	0.1	0.3	0.4	1.0	1.0	0.3	0.3	0.4	1.0	0.4	0.4		1.0	1.0	0.7	0.4	1.0	0.4	0.3	1.0	1.0	0.3	1.0	1.0	0.3	0.3	0.3	1.0	0.3	0.7	0.3	1.0	0.3	1.0	0.4		0.3	1.0		1.0	0.3	1.0		1.0	1.0	0.4
		0.8	0.8	0.8	0.7	0.7	0.8	1.0	1.0	0.8	0.9	0.8	0.8	0.8	0.7		0.7	0.8	0.8	0.8	0.9	0.8	0.9	0.9	0.9	0.7	1.0	0.8	0.7	1.0	0.8	1.0	0.7	0.8	0.9	1.0	0.8	1.0	0.8		0.7	1.0		1.0	1.0	1.0		0.9	1.0	0.8
Cleft palate	0.3	1.0	0.8	0.3	0.4	1.0	0.9	0.4	0.4	0.7	0.4	0.5	0.3	0.4	0.4	0.3	0.6	0.6	0.5	0.9	0.8	0.9	0.8	0.5	0.4	0.5	0.3	0.4	0.4	0.3	0.4	0.4	0.4	0.3	0.4	0.4	0.8	0.3	1.0	0.3	0.7	0.5	0.9	0.5	0.4	0.6	0.3	0.3	0.8	0.8
	0.9	0.7	0.9	0.7	0.7	0.8	0.9	0.9	1.0	1.0	1.0	0.8	0.9	0.9	1.0	0.9	0.8	0.8	0.9	0.9	0.9	0.9	0.9	0.7	0.9	0.7	1.0	0.8	0.7	0.7	0.7	0.7	0.8	1.0	0.8	1.0	0.8	0.8	0.8	0.8	0.8	0.8	0.9	0.8	0.7	0.7	0.8	0.9	0.8	0.8
Postnatal growth retardation	0.0	0.0	0.9	1.0	0.9	1.0	0.9	0.7	1.0	0.9	0.0	0.7		0.9	0.0		1.0	0.0	0.9	0.9	0.7	0.9	0.0	0.0	0.0	0.6	0.7	0.6	1.0	0.0	0.6	0.7	0.9	0.7	0.0	0.9		0.9	0.7	0.7	0.9		0.9	0.0	1.0	0.9			0.9	0.0
	0.9	0.7	1.0	0.8	0.8	0.8	0.8	1.0	0.8	0.9	0.8	1.0		0.7	0.7		0.8	0.8	0.9	1.0	0.8	0.9	0.7	0.7	0.7	0.8	1.0	0.8	0.7	0.8	0.7	1.0	0.9	0.7	0.9	0.8		0.8	0.8	0.8	0.7		0.8	0.9	0.7	0.7			0.9	0.8
Short stature	0.8	0.8	0.8	0.8	1.0	0.8	0.9	0.9	1.0	0.9	1.0	0.8	1.0	1.0	0.0	0.0	0.8	0.0	0.0	0.8	1.0	0.8	0.0	0.0	0.0	0.9	0.9	0.9	0.9	0.0	0.8	0.9	0.0	0.8	0.0	0.0	0.0	0.0	0.9	0.8	0.8	0.0	0.0	0.0	0.0	0.9	0.6	0.0	0.8	0.0
	0.9	0.7	0.9	0.8	0.8	0.8	0.8	1.0	0.8	1.0	0.7	1.0	0.8	0.7	0.8	0.7	0.8	1.0	1.0	1.0	0.7	0.8	0.8	0.8	0.8	0.8	1.0	0.7	0.7	0.7	0.7	1.0	0.8	0.8	0.8	0.8	1.0	0.9	0.8	0.7	0.8	1.0	0.8	1.0	0.9	0.7	0.7	1.0	0.8	0.8
Ptosis	0.0	0.4	1.0	0.5	1.0	0.9	0.9	0.9	0.9	0.9	0.3	0.5	0.0	0.3	0.5	0.9	0.0	0.5	0.0	1.0	0.4	0.0	0.9	0.4	0.3	0.0	0.9	0.9	0.0	0.9	0.9	0.0	1.0	0.9	0.9	0.0	1.0	0.5	0.9	0.0	0.0	0.3	0.4	0.0	0.0	0.0	1.0	0.9	0.9	0.9
	0.9	0.9	1.0	0.9	0.7	1.0	0.7	1.0	0.7	1.0	0.7	0.8	0.7	1.0	0.9	1.0	0.7	0.9	0.7	0.8	0.9	0.8	1.0	0.8	0.7	0.7	1.0	0.8	0.7	1.0	1.0	0.7	0.9	1.0	1.0	0.8	0.9	1.0	1.0	0.7	0.8	0.7	0.8	0.7	0.8	0.8	1.0	0.9	0.9	0.7
Triangular face		0.7	0.7	1.0	0.8	0.3	0.7	0.7	0.8	0.3	0.8	0.3		0.8	0.8		0.7	1.0	0.7		0.7	0.7	0.8	0.3	0.7	0.7	0.3	0.3	0.7	0.8	1.0	0.8	1.0		0.0	0.3		0.7	0.7		0.8	0.7	0.3	0.7	0.3	0.8		0.3	0.3	0.7
		0.7	1.0	0.8	0.7	0.8	0.8	0.7	0.8	1.0	0.8	1.0		0.9	0.7		0.8	0.9	0.8		0.8	0.8	0.9	1.0	0.8	0.9	0.9	1.0	0.7	0.8	0.8	0.8	0.8		0.8	1.0		0.7	0.8		0.7	0.8	0.9	0.7	0.9	0.7		0.8	1.0	0.8
Sensorineural hearing impairment	0.5	0.5	0.8	0.0	0.8	0.5	0.5	0.5	0.5	0.8	0.5	0.9	0.0	0.9	0.0		0.0	0.8	0.0	0.9	0.0	0.8	0.8	0.5	0.5	0.5	0.5	0.0	0.5	0.5	0.8	0.0	0.0	0.5	0.5	0.5	0.8	0.8	0.5	0.5	0.0	0.9	0.8	0.5	0.5	0.0	0.0	0.0	0.8	0.9
	0.8	0.7	1.0	0.7	0.9	0.8	0.7	0.9	0.8	1.0	0.7	0.9	0.7	0.8	0.7		0.8	1.0	1.0	0.8	0.8	0.9	0.8	0.8	0.7	0.8	0.7	0.7	0.9	0.7	0.7	0.8	1.0	0.9	0.7	0.8	1.0	0.7	0.8	0.8	1.0	0.8	0.9	0.7	0.8	0.8	0.8	1.0	1.0	0.8
Webbed neck	1.0	0.3	0.3	1.0	0.0	0.3	0.3	1.0	0.6	0.3	0.3	0.3	1.0	0.3	0.3	1.0	0.3	0.6	0.3		0.6		0.3	0.6	0.6	0.6	0.6		0.3		0.3	0.6	0.3	1.0	0.0	0.6	1.0	0.6	0.3	1.0	1.0	0.6		0.6	0.3	0.6	1.0	0.6	0.6	0.0
	1.0	0.9	0.8	0.9	0.8	1.0	0.8	1.0	0.8	0.8	0.9	0.7	0.9	0.8	0.8	1.0	0.8	0.8	0.9		0.8		0.7	0.8	0.8	0.7	0.8		0.8		0.8	0.8	0.7	0.9	0.8	0.8	1.0	0.8	0.8	1.0	1.0	0.8		0.8	0.8	0.8	1.0	0.8	0.8	0.8
Kyphoscoliosis	0.0	0.4	0.5	0.9	0.2	0.4	0.8	0.4	0.0	0.6	0.4	0.5		0.4	0.9		0.8	0.8	0.9	0.9	0.8	0.9	0.4	0.8	0.8	0.4	0.5			0.7	0.4		0.2	1.0	0.4	0.4	0.5	0.0	0.8	0.4	0.4	0.4		0.9	0.6	0.2		0.8	0.4	0.9
	0.8	0.8	1.0	0.7	1.0	1.0	0.8	0.8	0.9	0.7	0.7	0.8		0.8	0.7		1.0	0.8	0.8	0.9	0.8	0.9	1.0	0.8	0.8	0.7	0.8			0.7	1.0		0.8	0.8	0.8	0.8	0.7	0.8	0.8	0.8	0.7	0.7		0.7	0.7	0.8		0.8	0.7	0.9
Broad forehead	0.7	0.4	0.3	0.3	0.0	0.7	0.8	1.0	0.3	0.3	1.0	0.7	1.0	0.7	0.3	0.7	0.3	1.0	0.3		0.7		0.3	0.3	0.3	0.3	0.3	1.0	0.3	0.3	0.7	1.0	0.7		0.0	0.7		0.3	0.7			1.0	0.3	0.3	0.3	0.7	0.7		0.7	0.3
	0.8	0.7	0.9	0.8	0.9	1.0	0.8	0.8	0.8	1.0	0.9	1.0	0.8	0.8	0.9	0.8	0.8	0.9	0.8		1.0		0.9	0.9	1.0	0.9	0.8	0.9	1.0	0.7	1.0	0.8	0.8		0.8	0.8		0.8	0.8			0.8	1.0	0.7	0.9	0.9	0.8		1.0	0.9
Hypospadias	0.6	0.5	0.0	0.9	0.6	0.5	0.9	0.0	0.6	0.9	0.6	0.5	0.5	0.6	0.6		0.0	0.0	0.5	0.9	0.5	0.5	0.8	0.5	0.5	0.6	0.6		0.3	0.0	0.6	0.6	0.0		0.6	0.0	0.9	0.7	0.6	0.6	0.0	0.0	0.0	0.7	0.6	0.5		0.7	0.0	0.9
	0.9	0.7	0.8	0.8	0.9	0.7	0.8	1.0	0.7	0.8	0.8	0.7	0.7	0.8	0.7		0.8	0.8	0.8	0.8	0.7	0.7	0.8	0.7	1.0	1.0	1.0		0.8	0.8	0.8	1.0	1.0		0.7	1.0	0.8	1.0	0.7	0.7	0.9	0.9	1.0	0.8	0.8	0.7		1.0	0.9	0.8
Pectus excavatum of inferior sternum	1.0	0.2	0.3	1.0	0.0		1.0	0.5	1.0	0.2	0.2	1.0	0.5	0.5	1.0	0.5	1.0	0.5	0.4	1.0	0.3	0.3	0.2	1.0	0.2	0.2	0.3		1.0	1.0	1.0	0.5	1.0	0.3	0.3		0.1	0.0	0.2		1.0			0.5	0.3					0.2
	0.9	0.8	1.0	0.7	0.7		0.9	0.8	0.9	0.8	0.9	0.8	0.8	0.8	0.7	0.8	0.7	0.8	0.8	0.7	0.7	0.8	0.8	0.9	0.8	0.8	0.8		0.8	0.9	0.9	0.8	0.8	0.8	1.0		0.8	0.8	0.7		0.9			0.9	0.9					0.9
Cystic hygroma	0.7	0.7	0.3	0.7	0.0	0.3	0.7	0.7	0.7	0.3	0.3	0.3	0.7	0.3	0.3		0.7	0.7	0.3	0.3	0.7		0.3	0.7	0.7	0.7	0.7	0.7	0.3		0.3	0.7	0.3	0.7	0.3	0.7		0.7	0.7	0.0		0.7		0.7	0.3	0.7		0.7	0.7	0.3
	0.8	0.7	0.8	0.8	0.7	0.8	0.8	0.9	0.9	0.9	1.0	0.8	0.8	0.8	0.9		0.7	0.9	1.0	0.8	0.8		0.8	0.8	0.8	0.9	0.9	0.7	0.8		0.8	0.9	0.7	0.7	1.0	0.9		0.9	0.7	0.7		0.9		0.9	0.8	0.9		0.9	0.9	0.7
Shield chest	1.0	0.2	0.3	0.5	0.6		0.3	1.0		0.3	0.2	0.3	1.0	0.6	0.5	1.0	0.3	0.5	0.4	0.5	0.3	0.5	0.3	0.2	0.3	0.5	0.4		1.0	0.5	0.2	1.0	0.1	0.5	0.3	0.3	0.3	0.0	0.3		0.3	0.3		0.8	0.3		1.0			0.5
	1.0	0.8	1.0	0.8	0.9		0.8	1.0		0.8	0.8	0.7	1.0	0.8	0.8	0.8	0.8	0.8	0.7	0.8	0.9	0.8	0.9	0.8	1.0	0.7	0.8		1.0	0.9	0.8	1.0	0.8	0.8	0.8	0.7	0.8	0.8	0.8		0.9	0.8		0.8	0.9		1.0			0.8
Ventricular septal defect	0.7	0.9	0.7	0.0	0.7	0.7	0.3	0.0	0.0	0.5	0.5	0.7	0.7	0.0	0.6		0.0	0.0	0.5	0.0	0.9	0.7	0.7	0.0	0.0	0.7	0.0		0.0	0.0	0.0	0.5	0.0	0.7	0.0	0.7	0.7	1.0	0.5	0.0	1.0	0.5	0.7	0.0	0.7	0.0	0.0	0.0	0.0	0.7
	0.9	0.7	1.0	0.7	0.8	0.8	0.7	1.0	0.7	0.8	0.8	0.9	0.7	0.8	0.9		0.7	0.8	0.8	0.8	0.7	0.7	0.7	0.7	0.7	0.8	1.0		0.7	0.7	0.7	0.8	0.8	0.7	0.8	0.9	1.0	0.7	1.0	0.8	0.9	0.7	0.7	0.8	0.8	0.8	0.7	0.7	1.0	0.9
Low posterior hairline	1.0	0.7	0.3	0.3		0.3	0.5	0.6	0.3	0.3	0.5	0.3	1.0	1.0	0.3	1.0	0.3	0.3	0.3		0.3		0.3	0.3	0.3	0.3	0.3	0.3	0.3		0.3	0.5	1.0		0.7	0.3		0.5	0.3			0.3		0.7	0.3	1.0	1.0		0.3	0.0
	1.0	0.9	0.8	0.9		1.0	0.9	0.8	1.0	0.8	0.8	0.8	1.0	0.8	0.8	1.0	0.8	0.8	0.9		0.8		0.8	0.9	1.0	0.9	0.8	0.9	0.8		1.0	0.7	0.8		0.8	0.9		0.8	1.0			1.0		0.8	0.8	1.0	0.8		0.9	0.7
Dental malocclusion	0.0	0.4	0.3	0.3	0.0	0.3	0.5	0.7	0.4	0.4	0.4	0.4	0.3	0.5	0.4		0.9	0.7	0.5		0.3	0.3	0.4	0.9	0.4	0.5	0.3	0.3	0.5	0.3	0.3		0.4		0.3	0.4	0.9	0.3	0.3	0.0		0.4	0.4	0.4	0.4	1.0		0.3	0.5	0.4
	0.8	1.0	0.8	0.8	0.7	0.9	0.9	0.9	0.9	0.9	0.9	0.8	0.9	0.8	0.8		0.9	1.0	0.8		0.7	0.8	1.0	0.7	0.8	0.7	1.0	1.0	1.0	0.8	0.8		0.8		0.8	0.9	0.8	0.8	1.0	1.0		1.0	1.0	0.8	0.9	0.8		0.8	0.8	0.8
Clinodactyly	0.3	1.0	0.9	0.3	0.2	0.5	0.3	0.2	0.2	0.2	0.8	0.9	0.3	0.3	0.3	0.2	0.3	0.2	0.5	0.3	0.3	0.5	0.2	0.8	0.3	0.3	0.2	0.0	0.2	1.0	0.3	0.0	0.2	0.0	0.9	0.5	0.5	0.3	0.3	0.0	0.2	0.0	0.4	0.2	0.4	0.9	0.2	0.2	0.2	0.3
	0.9	0.7	1.0	0.8	1.0	0.8	0.8	0.8	0.7	0.8	0.9	1.0	1.0	0.8	0.8	0.8	0.8	0.7	0.8	0.7	1.0	0.9	0.9	0.9	0.7	0.8	1.0	0.7	0.7	0.7	0.7	0.7	0.7	0.8	1.0	0.8	0.8	0.7	1.0	0.8	0.9	1.0	0.7	1.0	0.8	0.9	0.9	0.8	0.8	0.8
Cryptorchidism	0.9	1.0	0.0	0.9	0.9	0.0	0.5	0.0	0.9	0.9	0.9	0.0	0.4	0.9	0.0		0.0	0.0	0.0		0.0		0.0	0.0	0.5	1.0	0.9		0.3	0.0	0.0	0.9	0.0		0.9			1.0	0.0	0.0		0.9		0.3	0.0	0.5		1.0	0.0	0.0
	1.0	0.8	0.8	0.8	1.0	0.8	1.0	0.9	0.8	1.0	0.8	0.8	0.8	0.8	0.7		0.8	0.8	0.8		0.8		0.7	0.7	0.9	0.9	1.0		0.7	0.8	1.0	1.0	0.9		0.7			0.8	0.8	0.8		0.7		0.8	0.9	0.7		0.7	1.0	0.8
Cubitus valgus	1.0	0.4	0.6	0.3			0.2	0.5		0.2	0.3	0.5	1.0	0.4	0.5	0.4	0.4	0.0	0.2	0.5	0.5		0.2	0.0	0.5	0.5	0.4			0.2	0.3		0.0	1.0	0.3		0.4	0.0	0.5	1.0	0.4			0.1	0.5	0.5	1.0	0.4		0.2
	1.0	0.8	0.8	0.8			0.7	0.8		1.0	0.8	0.9	1.0	0.7	1.0	0.7	1.0	0.9	1.0	0.7	0.8		0.8	0.9	0.8	0.9	0.8			0.8	0.7		0.8	1.0	0.8		0.7	0.9	0.8	0.7	0.7			0.7	0.7	0.9	1.0	0.8		0.9
Myopia	0.0	0.4	0.9	0.7	0.7	0.4	0.4	0.4	0.3	0.9	0.0	0.9		0.0	0.4	0.4	0.0	0.9	0.0	0.0	0.4	0.0	0.4	0.9	0.0	0.0	0.4	0.5	0.0	0.4	1.0		0.4	0.4	0.4	0.0	0.3	0.3	0.4	0.0		0.0	0.3	0.0	0.0	0.0		0.4	0.3	0.4
	0.8	0.9	1.0	0.9	0.7	0.9	0.8	0.9	0.7	0.8	0.7	0.8		0.8	0.8	0.9	0.8	0.8	0.9	0.9	0.9	0.7	0.9	0.7	0.8	0.7	0.8	0.7	0.7	0.9	0.7		0.8	0.8	0.9	0.8	0.8	0.8	0.9	0.7		0.8	0.8	0.7	0.8	0.8		0.9	1.0	1.0
Radial deviation of finger	0.3	0.2	0.7	0.4	0.2	0.5	0.4	0.4	0.0	0.3	0.7	0.7	0.4	0.3	0.4		0.4	0.0	0.2	0.3	0.7		0.4	0.7	0.4	0.4	0.4			0.3	0.3		0.2		0.7	0.4	0.3	0.0	0.4	0.3	0.2	0.0		0.2	0.8	0.7	0.4		0.3	0.3
	0.9	1.0	0.9	0.8	1.0	0.9	0.8	1.0	0.9	0.7	0.8	0.9	0.9	0.8	0.8		0.8	0.8	0.7	1.0	0.9		0.7	0.9	0.9	0.7	1.0			0.7	1.0		0.8		0.9	0.8	0.7	1.0	0.8	0.9	0.9	0.7		0.8	0.8	0.9	0.9		1.0	0.9
Feeding difficulties in infancy	0.3	1.0	0.3	0.7	0.0	0.3	0.3	0.0	0.3	1.0	0.5	0.3		0.0	0.6		0.3	0.0	0.0	0.3	0.3		0.3	0.3	0.9	0.0	0.3	1.0		0.3	0.6	0.6	0.0		0.3	0.0	0.3	0.3	0.5	0.6		0.3		0.3	0.3	0.0			0.0	0.3
	0.7	0.7	0.7	0.7	0.8	0.7	0.7	0.9	0.7	0.8	0.7	1.0		0.7	0.9		0.9	1.0	0.8	0.7	1.0		0.7	0.7	0.7	0.9	0.7	0.7		0.7	0.8	0.7	0.9		0.8	0.9	1.0	0.8	0.7	0.7		0.7		0.7	0.7	0.8			0.9	0.7
Brachydactyly	0.2	0.3	0.3	0.8	0.2	0.0	0.8	0.7	0.2	0.2	0.3	0.8	0.2	0.3	0.3		0.8	0.0	0.2	1.0	0.3	0.2	0.8	0.3	0.8	0.2	0.2		0.0	0.3	1.0	0.0	0.2	0.0	0.7	0.0	0.2	0.0	0.3	0.0	0.2	0.0	0.0	0.2	0.3	0.3	0.2	0.0	0.0	0.3
	0.7	0.7	0.9	0.8	0.8	0.9	0.8	0.7	0.8	0.7	0.8	0.9	0.9	0.8	0.7		0.8	0.8	0.7	0.7	0.9	0.8	0.7	0.9	0.7	0.9	0.8		0.9	0.7	0.7	0.7	0.8	0.9	0.8	0.7	0.9	0.7	0.8	0.9	0.9	1.0	0.7	0.9	0.9	0.8	0.9	1.0	1.0	0.8
Wide intermamillary distance		0.0		0.0		1.0	0.0		1.0	0.0	0.0	0.7	1.0	0.0	0.0	0.7	0.0	0.0	1.0		1.0			0.0	0.0	1.0	0.0		1.0			1.0	0.0		0.0			0.6	0.0	1.0				1.0			1.0			1.0
		0.7		0.8		1.0	1.0		0.8	0.8	0.7	0.8	0.7	0.8	0.7	0.7	0.8	0.9	1.0		1.0			0.9	1.0	1.0	0.8		0.9			1.0	0.8		0.7			1.0	0.7	1.0				0.9			1.0			1.0
Cafe-au-lait spot	0.4	0.5	0.0	0.0	0.9	0.4	0.4	0.4	0.0	0.0	0.3	0.0	0.6	0.0	0.5		0.4	0.0	0.0		0.0		0.0	0.5	0.6	0.4	0.4	0.7	0.4	0.4	0.0	0.4	0.0		0.6		0.5	0.0	0.4	0.0		0.8	0.5	0.4	0.0	0.8	0.6	0.8	0.0	0.0
	0.7	0.8	0.8	0.8	0.7	0.8	0.7	0.7	0.9	0.8	0.8	0.7	0.7	0.8	0.7		0.7	0.8	0.8		0.8		0.7	1.0	0.7	1.0	1.0	0.8	0.9	0.8	0.8	1.0	0.8		0.7		0.8	0.8	0.9	0.7		0.7	1.0	0.9	0.9	0.7	1.0	0.9	0.7	0.8
Hypogonadism	0.7	0.3	0.8	0.4	0.8	0.0	0.0	0.0	0.0	0.4	0.3	0.0	0.7	0.0	0.0		0.0	0.5	0.0	0.0	0.0		0.4	0.3	0.4	0.9	0.4	0.4	0.8	0.0	0.0		0.0	0.5	0.4	0.0	0.3	0.9	0.0	0.4		0.0		0.4	0.0	0.7			0.0	0.0
	0.8	0.8	0.8	0.8	0.8	0.8	0.8	0.7	0.8	1.0	0.7	0.8	0.9	0.7	0.8		0.8	0.8	0.8	0.7	1.0		0.7	0.8	0.8	0.9	0.9	0.7	0.8	0.7	0.8		0.8	0.8	0.7	0.9	0.8	0.9	0.9	0.8		1.0		0.7	0.8	0.7			1.0	0.8
Failure to thrive in infancy		0.0	0.5	0.5	0.6		0.5	0.5	0.5	0.5	0.0	0.0		0.0	0.0		0.0	0.0	0.0	0.8	0.8	0.5		0.0	0.0		0.5	1.0	0.8		0.0	0.5	0.0		0.0			0.0	0.5		1.0			0.0	0.0			1.0		0.0
		0.7	0.8	0.8	1.0		0.8	1.0	0.8	0.8	0.8	0.7		0.9	0.7		0.8	0.9	0.7	0.8	1.0	0.8		0.9	0.9		1.0	0.7	0.8		0.8	1.0	0.9		0.9			0.8	0.8		0.8			0.7	0.7			0.7		0.7

Table S20: Ontological and textual similarity scores for the top 50 articles annotated by stEngine bi-encoder ranked for Noonan syndrome

Phenotypes	27933191	35200700	17222357	38540405	24083159	34352903	32736638	35205257	35892827	24637303	25061003	36339399	34202860	27125300	22121397	25395808	24754368	34073526	38673696	37443678	32973676	34682127	30429698	38136934	19706204
Downslanted palpebral fissures	1.0	1.0	1.0	1.0	1.0	1.0	1.0	1.0	1.0	1.0	0.7	1.0	1.0	1.0	0.8	1.0	1.0	1.0	1.0	1.0	1.0	0.7	1.0	1.0	0.7
	1.0	1.0	0.8	0.9	0.8	0.7	0.9	1.0	1.0	1.0	0.9	0.8	1.0	0.8	0.8	1.0	1.0	1.0	1.0	1.0	0.8	0.7	0.8	1.0	1.0
Hypertelorism	1.0	1.0	1.0	1.0	1.0	1.0	1.0	1.0	1.0	1.0	0.4	0.8	0.4	1.0	1.0	1.0	1.0	1.0	1.0	1.0	0.4	1.0	1.0	1.0	1.0
	1.0	1.0	1.0	1.0	1.0	1.0	0.8	1.0	1.0	1.0	0.7	0.7	0.8	0.8	0.8	1.0	1.0	1.0	1.0	1.0	1.0	1.0	1.0	1.0	1.0
Ventricular septal defect	0.9	1.0	0.9	1.0	0.9	0.9	0.9	0.9	0.5	0.7	0.9	0.8	0.9	0.7	0.9	0.5	0.9	0.7	0.9	0.9	0.9	0.9	0.5	0.9	0.5
	0.8	1.0	0.7	1.0	0.8	1.0	0.8	0.8	0.8	1.0	0.7	0.8	1.0	0.8	0.8	0.8	0.8	0.9	0.9	1.0	0.7	1.0	0.8	0.8	0.8
Low-set ears	1.0	1.0	1.0	1.0	1.0	0.8	1.0	1.0	1.0	1.0	0.8	0.5	1.0	1.0	1.0	1.0	1.0	1.0	1.0	0.5	1.0	1.0	1.0	0.8	0.8
	0.8	0.8	1.0	0.8	0.9	1.0	0.8	1.0	0.9	1.0	0.8	1.0	0.8	1.0	0.9	0.9	1.0	0.8	0.8	0.8	0.9	0.8	1.0	0.8	1.0
Postnatal growth retardation	0.9	0.9	1.0	0.9	0.9	0.6	0.6	0.9	0.9	0.9	0.6	0.9	0.9	0.9	0.9	0.9	0.7	0.9	0.9	0.7	0.9	0.7	0.9	0.9	0.9
	0.9	0.8	0.7	0.8	0.8	0.7	0.8	0.9	0.8	0.8	1.0	0.8	0.9	0.8	0.9	0.7	0.7	0.8	0.9	0.7	0.9	0.8	0.9	0.8	0.9
Short stature	0.8	0.8	0.8	0.9	1.0	0.8	0.8	0.8	0.9	0.9	0.9	0.9	0.9	0.9	1.0	1.0	0.9	0.8	0.9	0.9	0.9	0.8	0.0	0.9	0.9
	0.8	1.0	1.0	0.7	1.0	1.0	0.8	0.7	0.9	0.8	0.8	0.8	0.7	0.7	1.0	1.0	0.7	0.8	0.7	0.7	0.7	1.0	0.7	0.7	0.8
Intellectual disability	0.9	0.9	0.9	0.7	0.8	0.9	0.8	0.9	0.9	0.9	1.0	0.9	0.7	0.9	0.9	0.9	1.0	0.9	0.9	0.9	0.8	0.9	0.8	0.9	0.9
	1.0	1.0	0.7	0.8	0.8	0.8	0.9	1.0	1.0	1.0	0.8	0.8	0.7	0.9	0.8	1.0	0.7	0.9	1.0	1.0	0.8	1.0	0.7	0.8	0.8
Micrognathia	0.7	0.7	0.9	0.9	0.9	0.9	0.8	0.9	1.0	1.0	0.7	0.8	0.9	0.9	0.9	0.9	0.9	0.9	0.9	0.9	0.5	0.9	0.9	0.6	0.9
	0.8	0.8	0.8	0.7	0.8	0.8	0.8	0.9	0.9	1.0	0.7	0.7	0.8	0.8	0.8	0.9	0.8	1.0	0.8	0.8	0.7	0.8	0.8	0.7	0.8
Pulmonic stenosis	0.9	0.9	0.9	0.9	0.9	0.9	0.9	0.7	1.0	0.9	0.9	0.9	0.7	0.9	0.8	0.4	0.9	1.0	0.8	0.9	0.7	0.9	0.9	0.9	1.0
	0.8	0.9	0.9	0.8	0.9	0.7	0.7	0.8	0.7	0.9	0.9	0.8	0.8	0.8	0.8	0.8	0.8	0.9	0.7	0.7	0.8	0.8	0.8	0.7	0.9
Atrial septal defect	0.9	1.0	0.9	0.9	0.9	0.7	0.9	0.9	1.0	0.9	0.9	0.5	0.9	0.9	0.9	0.5	0.7	0.9	0.9	0.9	0.7	0.9	0.5	0.9	0.5
	0.9	0.9	0.8	0.8	0.8	0.8	0.8	1.0	0.7	1.0	0.7	0.8	0.7	0.9	1.0	0.8	0.8	0.9	0.8	0.7	0.8	1.0	0.8	0.9	0.7
Cryptorchidism	0.9	0.9	0.9	0.9	1.0	0.9	0.9	0.9	0.9	0.9	0.9	0.9	0.9	0.9	0.9	1.0	0.9	0.9	0.9	0.5	0.9	0.9	0.9	0.9	0.7
	1.0	0.8	0.9	1.0	0.8	1.0	1.0	1.0	0.7	1.0	0.9	0.9	0.8	1.0	1.0	1.0	0.7	0.7	1.0	0.7	1.0	1.0	0.9	0.7	0.7
Ptosis	0.9	0.5	0.5	0.9	0.5	0.9	0.9	0.9	0.5	0.9	0.9	0.9	0.9	0.9	0.9	0.9	0.9	0.9	1.0	0.5	0.9	0.9	0.9	0.9	0.5
	1.0	0.8	0.7	1.0	0.7	0.8	0.8	0.8	0.9	1.0	0.8	0.8	1.0	1.0	0.9	1.0	1.0	1.0	0.8	0.8	1.0	1.0	1.0	1.0	0.9
Kyphoscoliosis	0.9	0.8	0.9	0.9	0.4	0.9	0.9	0.9	0.6	0.9	1.0	0.9	0.9	0.8	0.9	1.0	0.8	0.5	0.9	0.9	0.9	1.0	0.4	1.0	0.8
	0.9	0.8	0.7	0.8	0.7	0.8	1.0	0.9	0.9	0.8	0.8	0.7	0.8	0.8	0.7	0.8	0.8	0.8	0.8	0.9	0.7	0.8	0.7	0.8	0.8
High, narrow palate	1.0	0.4	1.0	1.0	1.0	1.0	1.0	1.0	0.4	0.4	0.6	0.4	1.0	1.0	1.0	1.0	0.3	0.4	0.6	1.0	0.3	1.0	1.0	1.0	1.0
	0.8	0.9	1.0	0.8	1.0	1.0	1.0	1.0	0.8	0.8	0.7	0.8	1.0	1.0	0.8	1.0	1.0	0.9	0.9	1.0	0.7	1.0	0.9	0.8	0.7
Hypertrophic cardiomyopathy	0.9	0.9	0.9	0.8	0.9	0.7	1.0	1.0	0.5	0.9	0.9	0.8	0.5	0.9	0.5	0.3	0.9	0.5	0.5	0.9	0.9	0.5	0.4	0.9	0.4
	1.0	0.9	0.8	0.7	0.8	0.8	0.7	0.8	0.8	1.0	0.8	0.7	0.8	1.0	0.8	0.8	0.8	0.8	0.8	1.0	0.8	0.7	0.8	0.8	0.9
Sensorineural hearing impairment	0.8	0.8	0.9	0.6	0.8	0.8	1.0	0.8	0.8	0.8	0.8	0.8	0.7	0.5	0.9	0.6	0.5	0.8	0.8	1.0	0.8	0.7	0.5	0.6	0.8
	0.9	1.0	0.8	0.9	0.7	0.8	0.9	0.9	0.9	0.8	0.7	1.0	0.8	0.7	0.8	0.7	0.7	0.8	1.0	0.7	1.0	1.0	0.7	0.7	0.8
Pectus excavatum of inferior sternum	1.0	1.0	0.7	1.0	0.7	0.5	1.0	1.0	1.0	1.0	0.7	1.0	0.8	1.0	0.3	0.5	1.0	0.4	1.0	1.0	0.3	1.0	0.3	1.0	1.0
	0.7	0.9	0.7	0.8	0.7	0.7	0.8	0.9	0.9	0.8	0.8	0.7	0.7	0.9	0.8	0.8	0.9	0.7	0.9	0.7	0.9	0.8	0.8	0.9	0.9
Epicanthus	1.0	1.0	0.6	1.0	0.6	0.6	1.0	0.9	1.0	0.6	0.6	0.6	1.0	0.6	1.0	0.9	1.0	1.0	0.6	0.6	0.6	0.6	1.0	1.0	0.6
	0.8	1.0	0.8	1.0	0.8	0.7	1.0	0.8	0.8	1.0	0.7	0.8	1.0	0.7	0.8	0.9	1.0	1.0	0.7	1.0	0.8	0.8	0.7	1.0	1.0
Triangular face	1.0	1.0	0.7	0.7	0.7	1.0	0.3	1.0	0.8	1.0	0.3	1.0	0.8	0.8	1.0	0.7	1.0	0.7	0.7	0.7	0.7	0.7	1.0	1.0	1.0
	1.0	1.0	0.8	0.8	0.8	0.8	0.9	0.8	1.0	1.0	0.8	0.7	1.0	0.8	0.7	0.8	0.7	0.7	0.8	0.7	0.7	0.8	0.8	0.7	0.8
Short neck	0.7	0.4	0.4	0.7	0.3	0.7	0.7	0.7	0.8	0.4	0.7	0.6	0.7	0.4	0.4	1.0	1.0	1.0	0.8	0.7	1.0	1.0	1.0	0.7	0.8
	0.7	0.8	0.8	0.8	0.7	0.7	0.9	0.8	0.8	0.8	0.7	1.0	0.8	0.8	1.0	0.8	0.8	1.0	0.8	0.8	0.8	0.8	0.8	0.8	0.9
Failure to thrive in infancy	1.0	0.9	1.0	0.9	1.0	0.8	0.5	1.0	0.6	1.0	0.9	1.0	0.7	0.7	1.0	1.0	0.0	0.7	1.0	0.9	0.6	1.0	0.0	1.0	0.8
	0.7	0.7	0.7	0.7	0.7	1.0	0.8	0.7	0.8	0.8	0.8	0.7	0.7	0.7	0.8	0.8	0.7	1.0	0.8	0.8	1.0	0.7	0.7	0.7	0.9
Cafe-au-lait spot	0.9	0.6	1.0	0.7	0.9	0.7	0.6	0.5	0.9	0.9	0.8	0.8	0.9	0.0	0.6	0.9	0.6	0.9	0.7	0.6	0.6	0.8	0.9	0.6	0.9
	0.9	0.7	0.7	0.8	0.8	0.8	0.7	0.8	0.8	0.9	1.0	0.8	0.7	0.7	0.8	0.8	0.7	0.7	0.7	0.8	0.8	0.9	0.9	0.8	0.9
Webbed neck	1.0	1.0	0.6	1.0	0.6	0.3	0.3	0.3	1.0	0.3	0.7	1.0	0.3	1.0	0.3	1.0	1.0	0.6	0.6	0.7	1.0	1.0	1.0	0.3	0.3
	0.8	1.0	0.8	1.0	0.8	0.7	1.0	0.7	1.0	0.8	0.7	0.8	0.7	0.9	0.9	1.0	1.0	0.9	1.0	0.8	0.9	1.0	0.7	0.7	0.7
Broad forehead	1.0	1.0	0.7	0.7	1.0	1.0	0.7	0.7	0.7	1.0	0.3	0.3	1.0	0.3	0.3	1.0	0.7	0.7	1.0	1.0	1.0	0.3	1.0	1.0	0.7
	0.8	0.8	0.9	0.8	0.8	0.8	0.8	1.0	0.8	0.8	0.7	0.8	0.7	0.7	0.8	1.0	0.8	0.8	0.8	1.0	0.8	0.8	0.8	0.8	0.8
Hypogonadism	0.9	0.9	0.8	0.8	0.8	0.9	0.6	0.4	0.8	0.9	0.6	0.9	0.8	0.8	0.9	0.9	0.4	0.8	0.8	0.9	0.9	0.7	0.4	0.8	0.9
	1.0	1.0	0.9	0.7	1.0	1.0	0.7	0.8	0.7	1.0	0.7	0.7	0.8	0.8	0.8	0.7	0.7	0.8	0.8	0.7	1.0	0.7	0.8	0.7	1.0
Feeding difficulties in infancy	1.0	0.6	1.0	0.9	1.0	0.6	0.6	0.9	0.9	1.0	0.9	0.9	1.0	1.0	1.0	0.6	0.3	0.6	0.9	0.9	0.6	0.6	0.6	1.0	0.6
	0.8	0.7	0.9	0.7	0.9	0.8	0.9	0.7	0.7	1.0	0.7	0.8	0.7	0.8	0.8	1.0	0.8	0.7	0.7	0.7	0.9	1.0	0.7	0.7	0.7
Hypospadias	0.9	0.6	0.6	0.9	0.6	0.7	0.7	0.9	0.7	0.9	0.7	0.6	0.7	0.6	0.9	0.9	0.6	0.9	0.9	0.6	0.6	0.7	0.6	0.6	0.7
	0.8	0.8	0.9	0.7	0.9	0.7	0.8	0.8	0.8	0.8	0.8	0.9	0.8	0.8	0.8	0.8	0.9	0.8	0.8	0.8	0.7	1.0	0.8	0.7	0.8
Low posterior hairline	0.8	0.8	1.0	1.0	1.0	0.8	0.5	0.7	0.7	0.8	0.7	0.6	1.0	0.0	0.5	0.7	1.0	0.8	0.5	0.3	0.7	0.8	0.7	0.7	0.7
	0.8	0.8	0.8	0.8	0.8	1.0	0.7	0.9	0.9	1.0	0.8	0.9	0.7	0.8	0.7	0.8	0.8	0.8	0.9	0.7	0.8	0.7	0.7	0.8	0.9
Coarctation of aorta	0.9	0.9	0.5	1.0	0.5	0.7	0.7	0.6	0.6	0.5	0.9	0.9	0.9	0.9	0.9	0.5	0.5	0.6	0.4	0.9	0.6	0.9	0.5	0.5	0.6
	0.8	0.8	0.9	0.8	0.8	0.8	0.8	0.8	0.7	0.9	0.9	0.8	0.9	0.8	0.8	0.7	0.9	0.8	0.8	0.9	0.9	0.9	0.8	0.7	0.8
Cleft palate	0.5	0.5	0.4	0.9	0.3	0.9	0.7	0.9	0.4	0.8	0.8	0.4	0.5	1.0	0.8	1.0	0.4	0.5	0.8	0.9	0.9	0.9	0.5	0.4	0.9
	0.7	1.0	0.7	0.8	0.8	0.8	0.9	0.8	0.7	0.8	0.8	0.8	0.7	0.8	0.7	0.7	0.7	0.8	1.0	0.9	0.8	0.9	0.8	1.0	0.9
Myopia	0.9	0.9	0.7	0.9	0.7	0.9	0.9	0.7	0.4	0.7	0.4	0.4	0.4	1.0	0.9	0.7	1.0	0.9	0.4	0.9	0.4	0.4	0.9	0.9	0.4
	1.0	0.8	0.7	0.7	0.7	0.8	0.8	0.9	0.8	0.7	0.8	0.8	0.9	0.8	0.7	0.7	0.8	1.0	0.7	0.8	0.7	0.9	0.7	0.8	0.7
Patent ductus arteriosus	0.9	0.9	0.7	1.0	0.9	0.8	0.5	0.5	0.6	0.4	0.9	0.8	0.9	0.5	0.5	0.3	0.4	0.5	0.4	0.6	0.8	0.9	0.3	0.5	0.5
	0.8	0.8	0.7	1.0	0.7	0.7	0.9	0.8	0.7	0.7	0.8	0.7	0.8	0.8	0.8	0.8	0.8	0.9	0.8	0.8	1.0	0.8	0.7	0.7	1.0
Lymphedema	0.7	0.9	0.9	1.0	0.9	0.7	0.7	0.7	0.7	0.9	0.9	0.9	0.5	0.2	0.2	0.2	0.9	0.9	0.7	0.5	0.5	0.7	0.9	1.0	0.7
	1.0	0.8	0.8	0.7	0.8	0.9	0.7	0.9	1.0	0.8	0.8	0.8	0.7	0.9	0.8	0.7	0.8	0.8	0.8	0.7	1.0	0.7	0.7	0.8	0.8
Cystic hygroma	0.3	0.3	0.9	0.7	0.9	0.3	0.7	0.3	0.7	0.3	0.8	0.9	0.3	0.3	0.8	0.7	0.7	0.7	0.8	0.8	0.7	0.7	0.8	0.3	0.3
	0.7	0.7	0.8	0.9	0.8	0.8	0.7	0.7	0.7	0.7	0.8	0.9	0.8	0.8	0.8	0.9	1.0	1.0	0.7	0.8	0.7	0.8	0.7	0.7	0.8
Juvenile myelomonocytic leukemia	0.4	1.0	1.0	0.4	0.7	0.7	0.3	0.3	1.0	0.4	0.6	1.0	0.7	1.0	0.7	0.4	0.8	0.4	0.3	0.3	1.0	0.4	0.7	0.3	0.9
	0.8	0.9	0.9	0.7	0.8	0.7	0.7	0.9	0.8	0.7	0.8	0.8	0.8	1.0	0.8	0.7	0.8	0.7	0.9	0.7	0.8	0.7	0.8	0.8	1.0

Phenotypes	17177115	29654333	28144274	30054457	29469822	35096710	29311971	17958891	36566191	34322401	35986401	29670795	38495807	19490664	32164556	35730652	36091172	22815589	34539746	34405046	37576110	28573359	37957608	26355968	29290338
Downslanted palpebral fissures	1.0	0.7	1.0	1.0	1.0	1.0	1.0	0.5	1.0	0.5	1.0	1.0	0.5	1.0	1.0	1.0	0.8	0.7	1.0	0.3	1.0	0.3	1.0	0.9	1.0
	1.0	1.0	1.0	1.0	1.0	1.0	0.9	0.9	0.7	0.8	1.0	1.0	0.7	1.0	1.0	1.0	0.9	0.8	0.9	1.0	1.0	0.8	1.0	0.7	1.0
Hypertelorism	0.4	0.8	1.0	1.0	1.0	1.0	1.0	1.0	1.0	0.8	1.0	1.0	0.4	1.0	1.0	1.0	1.0	0.8	1.0	0.8	0.8	0.4	1.0	1.0	1.0
	0.7	0.7	1.0	1.0	1.0	1.0	1.0	1.0	1.0	0.7	1.0	1.0	0.7	1.0	1.0	1.0	0.9	0.7	1.0	1.0	1.0	0.7	1.0	0.8	1.0
Ventricular septal defect	0.7	0.9	0.7	0.9	1.0	1.0	0.6	0.9	0.8	0.9	0.9	0.9	1.0	0.9	0.8	0.9	0.9	0.9	0.9	1.0	0.9	0.8	1.0	1.0	0.9
	0.8	0.8	1.0	1.0	0.8	0.8	0.8	0.8	0.8	0.8	0.8	0.9	0.7	0.8	0.7	0.8	0.7	0.7	0.9	0.9	0.8	0.7	0.7	0.8	1.0
Low-set ears	1.0	0.8	1.0	0.8	1.0	0.6	1.0	0.6	1.0	0.8	1.0	1.0	0.0	1.0	1.0	1.0	0.6	1.0	0.6	1.0	1.0	0.5	1.0	0.5	1.0
	1.0	1.0	0.8	0.8	0.8	0.7	0.8	0.8	0.8	0.9	0.9	0.8	0.7	0.8	0.8	0.8	0.8	0.8	0.8	0.8	0.8	1.0	0.9	0.8	0.8
Postnatal growth retardation	0.9	0.6	0.7	0.9	0.9	1.0	0.9	0.9	0.9	0.9	0.9	0.7	1.0	0.9	0.7	0.7	0.9	0.7	0.9	0.9	0.7	0.6	0.9	1.0	0.7
	0.8	0.8	1.0	1.0	0.8	0.7	0.8	0.7	0.7	0.8	0.7	1.0	0.7	0.9	0.7	1.0	0.7	0.7	0.8	0.7	0.8	1.0	0.9	0.8	1.0
Short stature	0.8	0.7	1.0	0.8	0.8	0.9	0.9	0.8	0.8	0.9	0.8	1.0	0.9	0.8	0.8	1.0	0.8	0.9	0.9	0.9	0.8	0.9	1.0	0.8	1.0
	0.9	0.8	1.0	0.8	1.0	0.8	0.7	0.8	0.8	0.7	0.7	1.0	0.7	1.0	0.7	1.0	0.8	0.9	1.0	0.7	0.8	0.7	0.8	1.0	1.0
Intellectual disability	0.8	0.7	0.9	0.9	0.8	0.9	0.9	0.9	0.7	0.9	0.9	0.9	0.9	0.7	0.4	1.0	0.7	0.7	0.9	0.8	0.9	0.8	0.8	0.9	0.9
	0.8	0.8	0.9	1.0	0.8	0.8	1.0	1.0	0.7	0.8	1.0	1.0	1.0	0.8	0.7	0.7	0.8	0.8	1.0	0.8	0.8	0.7	0.8	1.0	1.0
Micrognathia	0.6	0.8	1.0	0.9	0.7	0.9	0.9	0.7	0.6	0.7	0.7	0.6	0.9	1.0	0.9	0.9	0.9	1.0	0.9	1.0	0.7	1.0	0.9	0.9	0.9
	0.8	0.8	0.8	0.8	0.7	0.9	0.8	0.8	0.8	0.9	0.8	0.8	0.7	0.8	0.8	0.8	0.7	0.7	0.8	0.8	0.7	0.7	0.9	0.8	0.8
Pulmonic stenosis	0.9	0.9	0.4	1.0	0.9	0.5	0.8	0.9	0.9	0.5	1.0	0.9	0.9	1.0	0.9	0.7	0.9	0.9	0.7	0.3	1.0	0.8	0.5	0.7	0.9
	0.9	1.0	0.7	0.9	1.0	0.8	0.8	0.8	0.8	0.8	0.9	0.9	0.8	0.8	0.8	0.9	0.7	0.7	0.8	0.8	0.9	0.7	0.9	0.8	0.9
Atrial septal defect	0.9	0.9	0.9	0.9	1.0	1.0	0.5	0.9	0.9	0.6	0.9	0.9	1.0	0.9	0.9	0.7	0.7	0.9	0.9	0.9	0.9	0.9	1.0	0.9	0.9
	0.8	0.8	1.0	1.0	0.8	0.7	0.8	1.0	0.8	0.9	0.9	1.0	1.0	0.8	0.8	0.8	0.7	0.7	0.9	0.9	0.9	0.8	0.7	0.8	1.0
Cryptorchidism	1.0	0.9	0.9	0.9	0.0	0.6	0.9	0.9	0.9	1.0	0.9	0.9	0.9	0.9	0.9	0.9	0.6	0.9	0.4	0.9	0.0	0.6	0.9	0.7	0.0
	0.8	1.0	0.8	1.0	0.7	0.7	1.0	1.0	1.0	0.9	1.0	1.0	1.0	1.0	0.9	0.8	0.8	0.8	0.8	1.0	0.8	0.7	1.0	0.7	0.7
Ptosis	0.9	0.9	0.9	0.4	0.9	0.9	0.9	0.9	0.9	0.5	0.9	0.9	0.9	0.9	0.9	0.9	0.9	0.4	0.5	0.9	0.9	0.9	0.9	0.9	0.9
	0.7	0.7	1.0	0.9	1.0	0.8	1.0	0.8	1.0	0.7	1.0	1.0	0.8	0.9	1.0	0.8	1.0	0.8	0.8	0.7	0.8	0.8	1.0	0.7	1.0
Kyphoscoliosis	0.4	0.8	0.5	0.8	0.4	0.8	0.8	0.8	0.8	0.9	0.4	0.0	0.9	0.5	0.8	0.8	1.0	0.5	0.9	0.8	0.9	0.9	0.8	0.5	0.8
	0.8	0.8	0.9	0.8	0.8	0.8	0.8	0.8	0.8	0.8	0.8	1.0	0.7	0.7	0.8	0.8	0.8	1.0	0.9	0.7	0.9	0.7	0.8	0.7	0.8
High, narrow palate	1.0	0.4	1.0	1.0	1.0	1.0	1.0	1.0	1.0	0.4	0.4	1.0	0.6	1.0	1.0	0.4	0.4	1.0	0.6	0.3	1.0	1.0	0.3	1.0	0.4
	1.0	0.7	1.0	1.0	0.9	1.0	0.8	0.9	1.0	0.8	0.8	1.0	0.7	1.0	1.0	0.8	0.7	1.0	1.0	0.8	1.0	0.8	0.8	0.7	0.7
Hypertrophic cardiomyopathy	0.9	0.8	0.9	1.0	0.9	0.8	0.5	0.5	0.9	1.0	0.9	0.9	1.0	0.5	0.9	0.9	0.5	1.0	0.5	0.5	1.0	1.0	1.0	0.5	0.9
	0.9	0.9	1.0	0.8	0.7	0.8	0.8	0.7	0.9	0.8	0.8	1.0	1.0	0.8	0.8	0.8	0.7	0.8	0.8	0.8	0.7	0.8	0.8	0.7	0.7
Sensorineural hearing impairment	0.8	0.7	0.6	0.5	0.9	0.8	0.8	0.8	0.9	0.8	0.9	0.5	0.8	0.9	0.8	0.8	0.8	0.7	0.8	0.7	0.7	0.8	0.5	0.8	0.5
	0.8	1.0	0.7	0.7	0.8	1.0	0.7	0.7	0.8	0.8	0.8	0.9	0.8	0.9	0.7	1.0	0.8	0.8	1.0	0.8	0.8	0.7	0.8	0.7	0.7
Pectus excavatum of inferior sternum	1.0	0.7	0.3	1.0	1.0	0.5	0.8	1.0	1.0	0.5	0.5	1.0	0.5	0.8	1.0	1.0	0.5	0.5	1.0	0.5	1.0	0.5	0.5	0.5	0.5
	0.7	1.0	0.8	0.9	0.7	0.8	0.9	0.9	0.7	0.7	0.9	0.7	0.8	0.7	0.9	0.8	0.8	0.8	0.8	0.8	0.9	0.7	0.7	0.7	0.8
Epicanthus	0.6	0.5	1.0	0.6	0.9	0.6	0.6	0.9	0.6	0.6	0.6	0.6	0.5	0.9	0.6	1.0	1.0	0.9	1.0	1.0	1.0	0.3	0.9	0.5	0.6
	0.8	0.8	1.0	0.8	0.9	0.8	0.7	0.9	0.7	0.7	0.9	1.0	0.7	0.9	1.0	1.0	1.0	0.7	0.7	0.9	0.7	0.7	0.9	0.7	0.9
Triangular face	0.8	0.7	0.8	0.7	0.7	1.0	0.7	1.0	1.0	0.3	0.7	0.3	0.7	0.7	0.3	1.0	0.7	0.7	0.7	1.0	0.7	0.7	0.7	0.7	0.7
	0.9	0.8	0.9	0.8	0.7	0.8	0.7	0.7	0.8	0.9	0.7	0.8	0.7	0.8	0.8	0.8	0.7	0.8	0.9	0.8	1.0	0.8	0.8	0.8	1.0
Short neck	1.0	1.0	0.6	1.0	1.0	0.8	0.7	0.4	0.6	0.7	0.6	1.0	1.0	1.0	0.6	1.0	0.7	0.4	0.5	1.0	1.0	0.7	1.0	0.4	1.0
	0.8	0.7	0.8	1.0	1.0	0.7	0.8	0.8	0.8	0.7	0.8	1.0	0.7	1.0	0.7	0.8	0.9	0.8	0.8	0.7	1.0	0.7	1.0	0.7	0.8
Failure to thrive in infancy	0.8	0.8	0.5	0.8	0.5	0.7	0.6	0.8	0.8	0.9	0.7	0.5	0.9	1.0	0.0	1.0	1.0	0.8	0.6	0.8	0.7	0.9	0.9	0.5	0.7
	0.7	0.7	1.0	0.8	1.0	0.7	0.8	0.8	0.7	0.8	1.0	1.0	0.7	0.7	0.7	0.8	0.7	0.8	0.8	0.8	0.7	0.8	0.7	0.8	0.7
Cafe-au-lait spot	0.8	0.6	0.9	0.7	0.5	0.9	0.9	0.5	0.4	1.0	0.8	0.4	0.6	0.9	0.4	0.5	1.0	0.9	0.7	0.9	0.4	0.8	1.0	0.9	0.8
	0.7	0.8	0.7	0.8	1.0	0.9	0.9	0.7	0.7	0.8	0.8	0.8	0.7	0.8	0.7	0.8	0.8	0.8	0.7	0.9	0.8	1.0	0.7	0.8	0.9
Webbed neck	1.0	0.6	1.0	0.6	1.0	0.3	0.7	0.6	1.0	0.3	0.6	1.0	1.0	1.0	1.0	1.0	0.3	1.0	0.3	1.0	1.0	0.6	1.0	0.6	1.0
	0.7	0.7	1.0	0.8	1.0	0.8	0.8	0.9	0.8	0.7	0.7	0.8	1.0	0.8	1.0	0.9	0.7	0.7	0.9	1.0	1.0	0.8	0.9	0.9	1.0
Broad forehead	0.7	0.7	1.0	0.7	1.0	0.7	1.0	0.7	1.0	0.3	1.0	0.3	0.3	0.7	1.0	0.3	0.7	0.7	0.7	0.3	0.7	0.3	0.7	1.0	0.7
	0.7	0.7	0.9	0.8	0.8	1.0	0.9	1.0	0.9	0.7	0.9	0.8	0.8	0.9	1.0	0.8	0.8	0.8	1.0	0.8	1.0	0.7	1.0	0.8	0.8
Hypogonadism	0.7	0.9	0.4	0.4	0.4	0.7	0.9	0.4	0.4	0.9	0.7	0.4	0.9	0.4	0.6	0.4	0.9	0.6	0.9	0.6	0.5	0.9	0.8	0.5	0.4
	0.8	0.8	0.9	0.7	0.7	0.9	1.0	0.8	0.7	0.9	0.7	1.0	1.0	0.7	0.9	0.7	1.0	0.8	0.8	0.8	0.8	0.8	0.8	1.0	0.7
Feeding difficulties in infancy	0.9	0.7	0.0	1.0	0.3	0.7	0.4	0.6	0.5	1.0	0.5	0.5	1.0	0.4	0.3	0.9	0.7	0.5	0.3	0.6	0.0	0.8	0.5	0.8	0.6
	0.7	0.7	0.9	0.8	0.7	0.7	0.7	0.9	0.7	1.0	0.7	1.0	0.7	0.7	0.7	0.8	0.8	0.8	0.7	0.8	0.7	0.8	1.0	0.7	0.7
Hypospadias	0.6	0.9	0.6	0.6	0.0	0.7	0.7	0.9	0.6	0.9	0.9	0.6	0.9	1.0	0.6	0.6	0.9	0.8	0.4	0.9	0.4	0.8	0.6	0.4	0.7
	0.8	0.7	0.8	0.7	0.7	0.9	1.0	0.7	0.8	0.7	0.8	0.8	0.8	1.0	0.8	0.8	0.8	0.7	0.7	0.8	0.7	0.8	0.9	0.7	0.8
Low posterior hairline	0.8	0.7	0.7	0.3	1.0	1.0	0.8	0.5	0.3	0.5	0.7	0.7	0.7	1.0	0.3	0.3	0.5	0.5	0.7	1.0	0.6	0.6	0.6	0.5	1.0
	0.7	0.7	0.7	0.8	0.9	0.8	0.8	0.9	1.0	0.8	0.8	0.7	0.8	0.8	0.9	0.8	0.7	1.0	1.0	0.7	0.8	0.7	0.8	1.0	0.7
Coarctation of aorta	0.5	0.9	0.5	0.9	0.5	0.7	0.5	0.9	0.5	0.9	0.6	0.7	0.5	0.6	0.5	0.7	1.0	0.7	0.6	0.9	0.9	0.9	0.5	0.5	0.5
	0.9	0.7	0.8	0.7	0.9	0.8	0.8	0.8	0.7	0.9	0.8	0.8	0.8	0.8	0.7	0.7	0.8	0.7	0.7	0.9	0.8	0.7	0.7	0.8	0.9
Cleft palate	0.4	0.5	0.4	0.7	0.7	0.9	0.8	0.8	0.3	0.5	0.4	0.4	0.8	0.4	0.3	0.9	0.5	0.9	0.9	0.4	0.8	0.7	0.8	0.8	0.4
	0.7	0.7	0.9	0.9	0.9	0.9	0.8	0.9	0.8	0.7	0.8	1.0	0.7	0.8	0.9	0.9	0.8	0.7	0.9	0.7	0.7	0.9	0.9	0.7	0.7
Myopia	0.0	0.5	0.4	1.0	0.4	0.4	0.4	0.9	1.0	0.4	0.9	0.4	0.5	0.7	0.4	0.4	0.9	0.4	1.0	0.7	0.5	0.4	0.9	0.9	0.5
	0.8	0.7	0.9	0.8	0.9	0.8	0.8	0.8	0.8	0.8	0.8	0.9	0.8	0.7	0.9	0.8	0.8	0.7	0.8	0.9	0.8	0.7	1.0	0.7	1.0
Patent ductus arteriosus	0.5	0.8	0.4	0.9	0.4	0.5	0.3	0.9	0.4	0.8	0.4	1.0	0.4	0.4	1.0	0.5	1.0	1.0	0.9	1.0	0.7	0.5	0.9	0.5	0.4
	0.8	0.8	0.7	0.8	0.7	1.0	0.8	0.7	0.8	0.7	0.9	0.9	0.9	0.8	0.7	1.0	0.8	0.8	0.8	0.7	0.7	0.8	0.8	0.7	0.7
Lymphedema	0.2	0.7	0.7	0.7	0.7	1.0	0.5	0.7	0.9	0.7	0.5	0.2	0.9	0.5	0.7	0.2	0.3	0.7	0.7	0.5	0.7	0.7	0.2	0.7	0.0
	0.7	0.7	0.8	0.8	0.7	0.7	1.0	0.9	0.8	0.9	1.0	0.7	0.7	0.8	0.8	0.7	0.7	0.8	0.7	1.0	0.7	0.8	0.7	0.7	0.7
Cystic hygroma	0.7	0.7	0.9	0.7	0.9	0.3	0.7	0.7	0.9	0.7	0.9	0.9	0.7	0.9	0.9	0.7	0.3	0.3	0.3	0.7	0.7	0.7	0.7	0.7	0.7
	0.8	0.7	0.9	0.9	0.9	0.8	0.8	0.9	0.8	0.7	0.8	0.9	0.8	0.8	0.7	0.7	0.7	0.7	0.8	0.7	0.9	0.8	0.9	0.9	0.7
Juvenile myelomonocytic leukemia	0.4	0.8	1.0	0.4	0.7	1.0	0.6	0.3	1.0	0.8	0.9	1.0	0.8	0.3	0.3	0.3	0.7	0.8	0.6	0.7	0.3	0.7	0.3	0.4	1.0
	0.8	0.8	0.8	0.8	0.8	0.8	0.7	0.9	0.8	0.7	0.7	0.9	0.8	0.8	0.7	0.7	1.0	0.7	0.8	0.8	0.7	0.7	0.7	0.7	0.7
